# Pre-Participation Evaluation of Recreational and Competitive Athletes – A Systematic Review of Guidelines and Consensus Statements

**DOI:** 10.1186/s40798-025-00837-6

**Published:** 2025-04-05

**Authors:** Alina Weise, Nadja Könsgen, Christine Joisten, Fabian Schlumberger, Anja Hirschmüller, Jessica Breuing, Käthe Gooßen

**Affiliations:** 1 Witten/Herdecke University, Institute for Research in Operative Medicine (IFOM), Cologne, Germany; 2 Department for Physical Activity in Public Health, Institute of Movement and Neurosciences, German Sport University Cologne, Cologne, Germany; 3 Medical Center, Departement of Orthopedic Surgery and Traumatology, Albert-Ludwigs-University of Freiburg, Freiburg, Germany; 4 Altius Swiss Sportmed Center, Rheinfelden, Switzerland

**Keywords:** Consensus, Guideline, Physical activity, Pre-participation examination, Pre-participation screening, Recommendations, Sports medical screening

## Abstract

**Background:**

Pre-participation evaluation (PPE) aims to support safe participation in sports. The goal of this systematic review was to aggregate evidence- and consensus-based recommendations for the PPE of recreational or competitive athletes as preparation for developing a German guideline on this subject.

**Methods:**

Five databases, including MEDLINE, were searched in August 2022, complemented by searches on the websites of relevant guideline organisations and specialty medical associations and citation screening. We included guidelines/consensus statements with recommendations for PPE of adult recreational athletes or competitive athletes of any age, excluding those with certain chronic illnesses. We extracted and synthesised data in a structured manner and appraised quality using selected domains of the AGREE-II tool.

**Results:**

From the 6611 records found, we included 35 documents. Overall, the quality of the included documents was low. Seven documents (20%) made recommendations on the entire PPE process, while the remainder focussed on cardiovascular screening (16/35, 45.7%) or other topics. We extracted 305 recommendations. Of these, 11.8% (36/305) applied to recreational athletes and 88.2% (269/305) applied to athletes in organised or competitive sports. A total of 12.8% (39/305) of recommendations were directly linked to evidence from primary studies.

**Conclusion:**

Many recommendations exist for PPE, but only a few are evidence based. The lack of primary studies evaluating the effects of screening on health outcomes may have led to this lack of evidence-based guidelines and contributed to poor rigour in guideline development. Future guidelines/consensus statements require a more robust evidence base, and reporting should improve.

**Registration:**

PROSPERO CRD42022355112.

**Supplementary Information:**

The online version contains supplementary material available at 10.1186/s40798-025-00837-6.

## Key Points

Pre-participation evaluation aims to prevent possible harm during sports and later damage caused by exertion.

High-quality evidence for the effect of pre-participation evaluation on patient-relevant outcomes is lacking.

Recommendations in current guidelines and consensus statements are mostly consensus-based and focus on competitive athletes.

In the absence of clear benefits of certain evaluation components, choosing the best option depends on how individuals value the benefits and risks involved; shared decision-making should be the norm.

**Supplementary Information:**

The online version contains supplementary material available at 10.1186/s40798-025-00837-6.

## Introduction

Regular physical activity and sports can promote health and, in the long term, reduce the risk of premature death [1]. However, the 2019/2020 Current Health in Germany (‘Gesundheit in Deutschland aktuell’, GEDA) study by the Robert Koch Institute showed that only 23.3% of women and 29.4% of men are physically active at the recommended level [2]. Similarly, Bennie and Wiesner found that only 15.0% of adult Europeans met the recommendations for aerobic activity and muscle-strengthening exercises in the years 2013 and 2014 [3]. The COVID-19 pandemic led to a further decrease in time spent on moderate to vigorous physical activity during lockdown [4–6].

Despite the health-promoting effects of regular physical activity, performing sports can be related to health risks like cardiac arrest or arrhythmias. Maron referred to this phenomenon as the ‘paradox of exercise’ [7]. Sports-related health risks can affect athletes at any level. The incidence and prevalence of sports-related cardiac arrest differ among populations and regions [8]. For example, Berdowski et al. reported an incidence of 3.0 and 0.3 cases per 1 million people per year for people aged > 35 years and ≤ 35 years, respectively, in North Holland (Netherlands) [9]. Karam et al. reported an incidence of 7.0 per 1 million inhabitants per year for adults aged 18–75 years in the Greater Paris area (France) [10]. These differences in incidence may be attributed to the varying risk factors across populations. For example, the risk of sports-related cardiovascular incidents increases exponentially in people older than 35 years [9, 11].

Injuries are an additional risk of sports participation. A systematic review (SR) that Al-Qahtani et al. [12] conducted showed that the prevalence of sports-related injuries in adolescent athletes ranges from 34 to 65%. Risk factors for injury include the type of sports and training practices. Prevention of such injuries avoids not only their direct consequences (e.g. required treatment and absence from school or work) but also potential long-term physical constraints or sequelae.

Pre-participation evaluation (PPE) is a preventive health examination used in sports medicine that may help people to start or resume physical activity safely by identifying those at an increased risk of adverse events during exercise. Its aims are to reduce the adverse effects of physical activity and prevent any subsequent damage caused by exertion. According to Whitfield et al., up to 95.5% of Americans > 40 years of age are eligible for PPE [13, 14]. For participants in official sports competitions, such as squad athletes, PPE is often a standard procedure [15]. However, there is a debate over which components (e.g., which diagnostic assessments) should be included in a PPE of competitive and recreational athletes [11]. Limited evidence for the effects of PPE on patient-relevant health outcomes, as well as methodological challenges for conducting high-quality studies, have contributed to this debate [16]. Therefore, current PPE recommendations and clinical practice seem to rely mostly on clinical expertise.

With this SR, we aimed to aggregate and appraise evidence- and consensus-based recommendations for PPE. We used the findings of this SR to develop a new German consensus-based guideline for PPE of recreational athletes [17].

## Methods

We performed this SR according to the methods pre-defined in a protocol registered in PROSPERO (CRD42022355112). We reported the SR according to the *Preferred Reporting Items for Systematic Reviews and Meta-Analyses* (PRISMA) 2020 [18] and the *Implementing PRISMA in Exercise*,* Rehabilitation*,* Sports Medicine and Sports Science* (PERSiST) guidance [19]. We contacted the authors of the included documents only for full text retrieval.

### Population, Intervention, Control and Outcome Questions and Eligibility Criteria

This SR included evidence- or consensus-based guidelines and consensus statements about the PPE of apparently healthy adults and athletes of all ages (both groups with and without disabilities). It did not include guidelines or consensus statements targeting people with chronic diseases, such as cardiovascular disease or diabetes mellitus, due to the specific risks in this population and their need for adjusted PPE. The primary outcomes were the prevention or reduction of fatal events during sports and the possible sequelae of participation in individual sports. We included documents published in English and German. Expired documents and recommendations for countries outside the World Health Organisation (WHO) mortality stratum A [20] were ineligible. All pre-defined eligibility criteria are presented in Table 1.

**Table 1:** Pre-defined eligibility criteria

	Inclusion	Exclusion
Population	Healthy adults with or without disabilities Competitive athletes of any age and level with or without disabilities	Primary population with known non-communicable chronic diseases, such as cardiovascular disease or diabetes mellitus
Intervention	Medical history Anthropometric measurements Cardiometabolic or internal medicine examinations Orthopaedic examinations Additional tests or diagnostic procedures from related disciplines	Diagnostic interventions specific to sports for which there is a separate fitness examination (e.g. diving, flying)
Comparison	Other medical or family history questions or diagnostic parameters	
Outcome	Prevention or reduction of exercise-induced (fatal) events during sports participation Prevention or reduction of possible sequelae of sports or exertion Diagnostic test accuracy measures	
Study type	Evidence-based and/or consensus-based guidelines and recommendations	Expired documents Documents published before 2012
Setting	WHO mortality stratum A countries [20] Published by professional medical societies, guideline organisations, public or government-led organisations (e.g. the military) or expert groups appointed by such societies or organisations	
Language	English, German	
Other		Duplicates Multiple publications without additional information

### Literature Search

We systematically searched for literature on MEDLINE (PubMed), Trip Database, the National Institutes of Health (NIH) Library, the Guidelines International Network’s (GIN) International Guidelines Library, and ECRI Guideline Trust’s guideline repositories. We used automation tools (Word Frequency Analyzer, SearchRefinery [21]) to facilitate the development of the MEDLINE search strategy and adapted the strategy to the syntax of each database. Searches contained index and free text terms for population, intervention and study type, as applicable. We performed database searches in August 2022, with 1 January 2012 as the start date. We chose this start date because around 50% of guidelines are out of date within five years [22, 23]. Therefore, any guideline older than 10 years was presumably outdated. No restrictions on language or publication status were applied at the search stage.

In addition, we performed structured hand searches on the websites of the following specialty medical associations and guideline organisations: American College of Sports Medicine, British Association of Sport & Exercise Medicine, Canadian Academy of Sport and Exercise Medicine, Sports Medicine Australia, European Federation of Sports Medicine Associations, Canadian Medical Association Infobase of Clinical Practice Guidelines, Australian National Health and Medical Research Council, National Institute for Health and Care Excellence, New Zealand Guidelines Group via the Ministry of Health New Zealand and VA/DoD Clinical Practice Guidelines. We performed website searches in August and October 2022.

The full search strategies, including search dates, are provided as Supplementary Information (Supplement I).

### Document Selection

We exported all records found via MEDLINE to Endnote (Endnote, Version: 20 [Software]. Clarivate, Boston, Massachusetts, USA. https://endnote.com/) and removed duplicates. All records found via websites and other databases were exported to Microsoft Excel (2016). We performed document selection according to pre-defined eligibility criteria (Table 1). We then screened the titles and abstracts of all MEDLINE records using the web tool Rayyan [24]. The other records were screened in Excel based on their titles. We obtained the full texts of all records deemed potentially relevant for full text screening. Notably, two independent researchers (AW, KG) performed all screening steps. We discussed differences until a consensus was reached. If a consensus could not be reached via such discussions, we consulted clinical experts (AH, CJ).

In addition, we performed a backward citation search via Scopus, including all eligible documents that were found via the database and website searches and that were available on Scopus. The identification of references was facilitated using Scopus, and deduplication was performed via Endnote. The screening process was identical to that used for the MEDLINE records (AW, NK).

### Quality Appraisal

We appraised the quality of included documents using two selected domains from the Appraisal of Guidelines for Research and Evaluation II (AGREE II) tool [25]. The tool comprises six domains, namely, (1) Scope and Purpose, (2) Stakeholder Involvement, (3) Rigour of Development, (4) Clarity of Presentation, (5) Applicability and (6) Editorial Independence. Due to resource restrictions, we limited the quality appraisal to domains 3 and 6, which were the most relevant for our purposes. Domain 3 consists of eight items, while domain 6 consists of two items. Two independent researchers (AW, KG, NK) rated each item on a seven-point Likert scale (higher scores mean higher quality). Depending on the scores per individual item and rater, each domain could achieve a score of 0–100%.

Two independent researchers (AW, KG) pilot-tested the quality appraisal process using six included documents. Of those, we chose three documents based on heterogeneous characteristics (e.g. content and structure) and three other documents at random. We compared and discussed the appraisal of the pilot sample to agree on specific appraisal criteria, ensure consistency and reduce systematic differences. An additional researcher (NK) joined the team later and performed pilot testing using a sample of four documents.

Two researchers (AW, NK) performed all further ratings independently. Afterwards, they discussed deviations of two or more points per item to identify and correct systematic differences in appraisals, as necessary. We calculated the mean scores per document and per domain. For each quality appraisal item, we calculated median scores across documents and the corresponding ranges.

### Data Extraction and Data Items

We extracted data into a form (Microsoft Excel, 2016) that we developed for this review. We piloted the form during two sessions, each using three of the documents that we previously selected for the pilot quality appraisal. Two researchers (AW, KG) performed both piloting sessions independently then modified and expanded the data extraction form based on the consensus they reached. Two additional researchers (FS, NK) joined the team later and performed pilot testing using samples of three and four documents, respectively.

One of the four researchers (AW, FS, KG, NK) extracted data into the piloted form, and a second verified data extraction. We discussed differences until a consensus was reached, including a third researcher as necessary. Extracted data included information about the guideline or consensus statement; population, intervention, control and outcome (PICO) elements; and recommendations. We extracted recommendations labelled as such and recommendation-like sentences from the main document text. A list of all data extraction items can be found on PROSPERO (CRD42022355112).

### Levels of Evidence and Strength of Recommendations

For recommendations that were directly linked to the literature in the original publications, we extracted the corresponding references and obtained their full texts. We assigned a level of evidence (LoE) for primary studies or SRs based on their full texts, according to the *Oxford 2011 Levels of Evidence* [26]. Two researchers (NK, KG) independently assessed the first 20 references then sought a consensus. One researcher (NK) completed all further assignments. When doubts arose, a second researcher (KG) was consulted. We did not assign LoEs to narrative review, commentary or guideline (without SR) references.

We extracted the grade or strength of the recommendation when available. For recommendations labelled as such but that did not have an assigned strength of recommendation or linked evidence, we assigned a level C according to the strength of recommendation taxonomy (SORT), which uses a scale from A (strongest) to C (weakest) [27]. For recommendation-like sentences that were extracted from the text, we complemented the SORT with an additional level (–) (Table 2).

**Table 2:** Expanded strength of recommendation taxonomy [27]

Strength of recommendation	Definition
A	Recommendation based on consistent and good-quality patient-oriented evidence^a^
B	Recommendation based on inconsistent or limited-quality patient-oriented evidence^a^
C	Recommendation based on consensus, usual practice, opinion, disease-oriented evidence* or case series for studies of diagnosis, treatment, prevention or screening
–	Statement in the text

^a^ Patient-oriented evidence measures outcomes that matter to the patient: morbidity, mortality, symptom improvement, cost reduction and quality of life. Disease-oriented evidence measures intermediate, physiologic or surrogate end points that may or may not reflect improvements in patient outcomes (e.g. blood pressure, blood chemistry, physiologic function, pathologic findings)

### Statistical Analysis

We calculated values and percentages for the nominal data on the characteristics of included documents and recommendations. For calculations, we used Microsoft Excel (2016).

### Synthesis of Documents and Recommendations

We synthesised data using a structured, narrative format. We presented the metadata of the included documents using tabulation. We also grouped extracted recommendations according to their clinical topics (e.g. cardiology, anthropometrics) and provided short summaries for each. We prepared supplementary tables containing all recommendations extracted (including data on the population and type and strength of the recommendation, as well as the LoE of the underlying primary studies).

### Deviations from the Protocol

For the systematic literature search, we planned to conduct forward and backward citation screening. However, due to a high inclusion rate and limited resources, we omitted the forward citation screening.

We pre-defined several outcomes and measures of effect to extract. Due to an unexpectedly high number of recommendations, we decided to focus on recommendations for the type and scope of PPE. We did not systematically extract recommendations for interventions for training, nutrition or other topics, nor for follow-up evaluations.

## Results

**Fig. 1:**
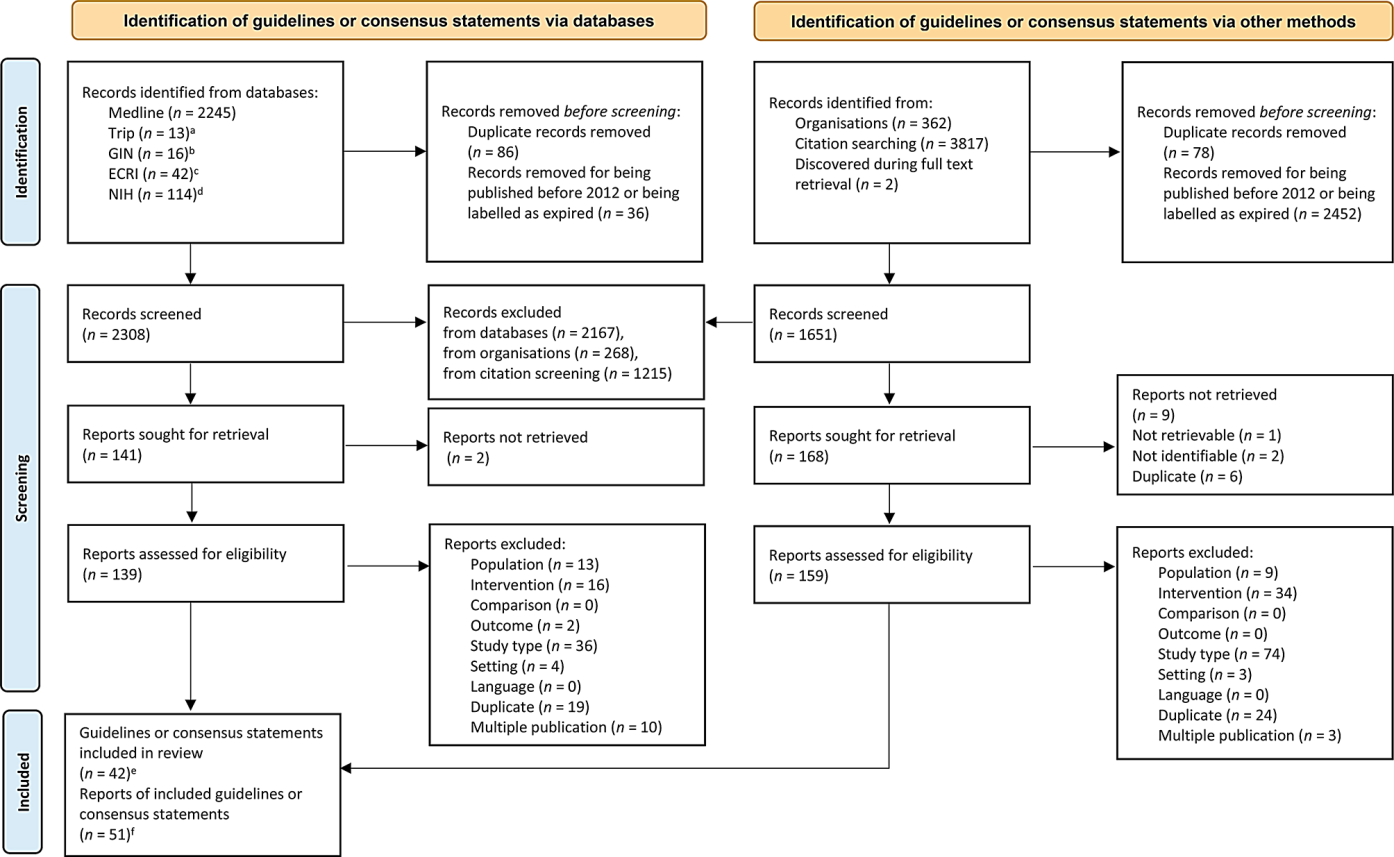
PRISMA flowchart. ^a^TRIP Medical database; ^b^Guidelines International Network’s (GIN) International Guidelines Library; ^c^ECRI Guideline Trust’s guideline repositories; ^d^National Institutes of Health (NIH) Library. ^e^ This included *n* = 7 guidelines or consensus statements about sports following a COVID-19 infection, which were not part of the current manuscript. ^f^ This included *n* = 8 reports on sports following a COVID-19 infection, which were not part of the current manuscript

The systematic searches yielded 6611 records, and the document selection process is depicted in Fig. 1. After removing duplicates, expired documents and documents published before 2012, we screened 3959 titles and abstracts then assessed the eligibility of 298 full texts. Forty-two guidelines and consensus statements^1^ published in 51 reports met our eligibility criteria, of which 7 documents published in eight reports addressed sports following a COVID-19 infection and were therefore not part of the current manuscript. Therefore, we finally included 35 documents published in 43 reports [28–70]. A list of references that were excluded based on the full text, including the primary reason for exclusion, can be found in the Supplementary Information (Supplement II).

### Characteristics of Included Documents

**Fig. 2:**
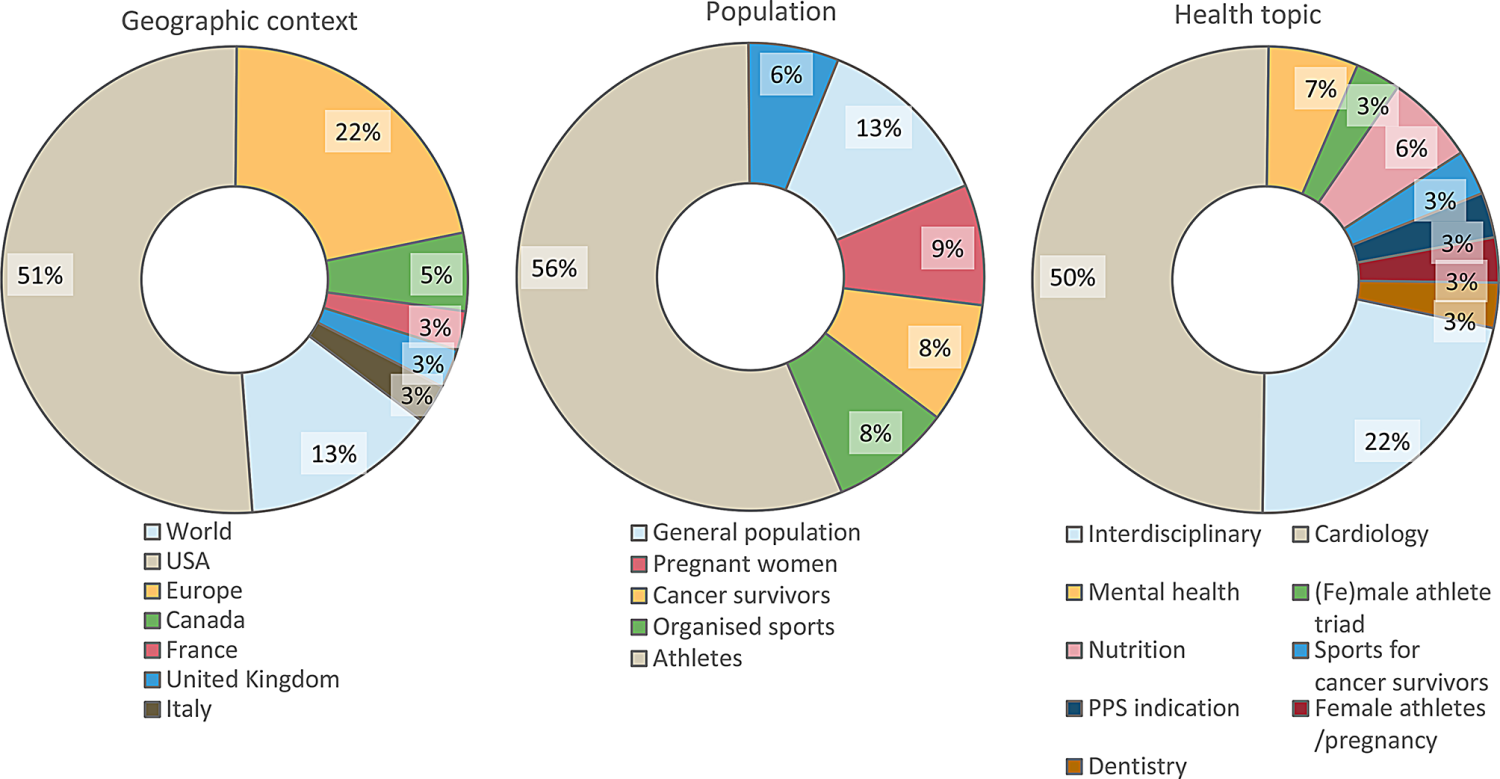
Characteristics of included documents by geography, population and topic (*n* = 35 documents)

Included documents were published between 2012 and 2022. Most were from the USA, but several other geographic contexts were also represented. The target population was mainly composed of athletes, but other levels of sports participation were also addressed. In terms of the health topic, close to half of the documents focussed on cardiology (see Fig. 2). An overview of the characteristics of included documents is provided in Table 3.

**Table 3:** Characteristics and quality appraisal of included documents

ID, ref.	Title Publishing organisation	Country, year	Population
AAFP 2016 [28]	*Selected Issues in Injury and Illness Prevention and the Team Physician: A Consensus Statement* Unclear (several)	USA, 2016	Athletes
AAFP 2017 [29]	*Female Athlete Issues for the Team Physician: A Consensus Statement – 2017 Update* American Academy for Family Physicians	USA, 2017	Female athletes, pregnant athletes
AAP 2019 [31]	*Preparticipation Physical Evaluation*,* 5th Edition* American Academy of Pediatrics	USA, 2019	Athletes in organised sports or vigorous physical activities
ACOG 2020 [30]	*Physical Activity and Exercise During Pregnancy and the Postpartum Period: ACOG Committee Opinion*,* Number 804* American College of Obstetricians and Gynecologists	USA, 2020	Pregnant women
ACPM 2013 [54]	*Screening for Sudden Cardiac Death Before Participation in High School and Collegiate Sports: American College of Preventive Medicine Position Statement on Preventive Practice* American College of Preventive Medicine	USA, 2013	High school and college athletes
ACSM 2019 [38]	*Exercise Guidelines for Cancer Survivors: Consensus Statement from International Multidisciplinary Round Table* American College of Sports Medicine	USA, 2019	Cancer survivors
ACSM 2021 [32]	*ACSM’s Guidelines for Exercise Testing and Prescription*,* 11th Edition* American College of Sports Medicine	USA, 2021	General population (including pregnant women and cancer survivors)
AEPC 2017 [48]	*Cardiovascular Pre-participation evaluation in Young Athletes: Recommendations of the Association of European Paediatric Cardiology* Association of European Paediatric Cardiology	Europe, 2017	Young athletes
AHA ACC 2015 [36, 57, 58]	*Eligibility and Disqualification Recommendations for Competitive Athletes with Cardiovascular Abnormalities: Preamble*,* Principles*,* and General Considerations; Task Force 2: Preparticipation Screening for Cardiovascular Disease in Competitive Athletes; Task Force 6: Hypertension: A Scientific Statement from the American Heart Association and American College of Cardiology* American Heart Association, American College of Cardiology	USA, 2015	General population, participants in organised sports, athletes
AMSSM 2017 [45]	*AMSSM Position Statement on Cardiovascular Preparticipation Screening in Athletes: Current Evidence*,* Knowledge Gaps*,* Recommendations and Future Directions* American Medical Society for Sports Medicine	USA, 2017	Athletes
AMSSM 2017 (ECG) [46]	*International Criteria for Electrocardiographic Interpretation in Athletes: Consensus Statement* Unclear (several)	World, 2017	Athletes
AMSSM 2020 [41]	*Mental Health Issues and Psychological Factors in Athletes: Detection*,* Management*,* Effect on Performance and Prevention: American Medical Society for Sports Medicine Position Statement – Executive Summary* American Medical Society for Sports Medicine	USA, 2020	Athletes
ASE 2020 [33]	*Recommendations on the Use of Multimodality Cardiovascular Imaging in Young Adult Competitive Athletes: A Report from the American Society of Echocardiography in Collaboration with the Society of Cardiovascular Computed Tomography and the Society for Cardiovascular Magnetic Resonance* American Society of Echocardiography	USA, 2020	Young athletes
BSE CRY 2018 [64]	*A Guideline Update for the Practice of Echocardiography in the Cardiac Screening of Sports Participants: A Joint Policy Statement from the British Society of Echocardiography and Cardiac Risk in the Young* British Society of Echocardiography and Cardiac Risk in the Young	UK, 2018	Young athletes
CASEM 2020 [69, 71]	*Physical Activity Prescription: A Critical Opportunity to Address a Modifiable Risk Factor for the Prevention and Management of Chronic Disease: A Position Statement by the Canadian Academy of Sport and Exercise Medicine* Canadian Academy of Sport and Exercise Medicine	World, 2020	General population
CCS CHRS 2019 [52]	*Canadian Cardiovascular Society/Canadian Heart Rhythm Society Joint Position Statement on the Cardiovascular Screening of Competitive Athletes* Canadian Cardiovascular Society, Canadian Heart Rhythm Society	Canada, 2019	Athletes
COCIS 2021 [34, 35, 44]	*Italian Cardiological Guidelines for Sports Eligibility in Athletes with Heart Disease: Part 1; Part 2; Italian Cardiological Guidelines (COCIS) for Competitive Sport Eligibility in Athletes with Heart Disease: Update 2020* Italian Society of Sports Cardiology and the Italian Sports Medicine Federation	Italy, 2021	Athletes
EA4SD 2020 [67]	*The European Association for Sports Dentistry*,* Academy for Sports Dentistry*,* European College of Sports and Exercise Physicians Consensus Statement on Sports Dentistry Integration in Sports Medicine* European Association for Sports Dentistry	Europe/ USA, 2020	Athletes of all levels
EAPC EACVI 2018 [49, 65]	*The Multi-modality Cardiac Imaging Approach to the Athlete’s Heart: An Expert Consensus of the European Association of Cardiovascular Imaging; European Association of Preventive Cardiology (EAPC) and European Association of Cardiovascular Imaging (EACVI) Joint Position Statement: Recommendations for the Indication and Interpretation of Cardiovascular Imaging in the Evaluation of the Athlete’s Heart* European Association of Cardiovascular Imaging	Europe, 2018	Athletes, elite athletes
EFSMA 2015 [53]	*The Pre-participation Examination in Sports: EFSMA Statement on ECG for Pre-participation Examination* European Federation of Sports Medicine Associations	Europe, 2015	Recreational to elite athletes
EFSMA 2021 [51]	*Preparticipation Medical Evaluation for Elite Athletes: EFSMA Recommendations on Standardised Preparticipation Evaluation Form in European Countries* European Federation of Sports Medicine Associations	Europe, 2021	Elite athletes
EHRA EACPR 2017 [59]	*Pre-participation Cardiovascular Evaluation for Athletic Participants to Prevent Sudden Death: Position Paper from the EHRA and the EACPR*,* Branches of the ESC. Endorsed by APHRS*,* HRS*,* and SOLAECE* European Heart Rhythm Association, European Association for Cardiovascular Prevention and Rehabilitation	Europe, 2017	Athletes
ESC 2021 [66]	*2020 ESC Guidelines on Sports Cardiology and Exercise in Patients with Cardiovascular Disease* European Society of Cardiology	Europe, 2021	General population (including cancer survivors), athletes
ESC 2022 [70]	*2022 ESC Guidelines for the Management of Patients with Ventricular Arrhythmias and the Prevention of Sudden Cardiac Death* European Society of Cardiology	Europe, 2022	Middle aged and older individuals, athletes
FATC 2014 [43]	*2014 Female Athlete Triad Coalition Consensus Statement on Treatment and Return to Play of the Female Athlete Triad* Female Athlete Triad Coalition	USA, 2014	Female athletes
FMATC 2021 [47, 62]	*The Male Athlete Triad – A Consensus Statement from the Female and Male Athlete Triad Coalition Part 1: Definition and Scientific Basis; Part II: Diagnosis*,* Treatment*,* and Return-To-Play* Female and Male Athlete Triad Coalition	USA, 2021	Male athletes
FSC 2019 [55, 56]	*French Society of Cardiology Guidelines on Exercise Tests (Part 1): Methods and Interpretation; (Part 2): Indications for Exercise Tests in Cardiac Diseases* French Society of Cardiology	France, 2019	Athletes
IOC 2013 [68]	*How to Minimise the Health Risks to Athletes Who Compete in Weight-sensitive Sports Review and Position Statement on Behalf of the Ad Hoc Research Working Group on Body Composition*,* Health and Performance*,* Under the Auspices of the IOC Medical Commission* International Olympic Committee	World, 2013	Athletes in weight-sensitive sports
IOC 2017 [37]	*Exercise and Pregnancy in Recreational and Elite Athletes: 2016/2017 Evidence Summary from the IOC Expert Group Meeting*,* Lausanne. Part 5. Recommendations for Health Professionals and Active Women* International Olympic Committee	World, 2017	Pregnant and postpartum recreational and elite athletes
IOC 2018 [60, 61]	*The IOC Consensus Statement: Beyond the Female Athlete Triad – Relative Energy Deficiency in Sport (RED-S); International Olympic Committee (IOC) Consensus Statement on Relative Energy Deficiency in Sport (RED-S): 2018 Update* International Olympic Committee	World, 2018	Athletes
NATA 2012 [40]	*National Athletic Trainers’ Association Position Statement: Preventing Sudden Death in Sports* National Athletic Trainers’ Association	USA, 2012	Participants in organised sports
NATA 2013 [39]	*The Inter-association Task Force for Preventing Sudden Death in Secondary School Athletics Programs: Best-practices Recommendations* Unclear (several)	North America, 2013	Secondary school athletes
NATA 2014 [42]	*National Athletic Trainers’ Association Position Statement: Preparticipation Physical Examinations and Disqualifying Conditions* National Athletic Trainers’ Association	USA, 2014	Participants in organised sports
NATA 2015 [63]	*Inter-association Recommendations for Developing a Plan to Recognize and Refer Student-athletes with Psychological Concerns at the Secondary School Level: A Consensus Statement* Unclear (several)	USA, 2015	Secondary school athletes
NCAA 2016 [50]	*Inter-association Consensus Statement on Cardiovascular Care of College Student-athletes* National Collegiate Athletic Association	USA, 2016	College athletes

### Quality of Included Documents

Overall, the quality of the documents was low for both domains selected for appraisal. The quality appraisal for domain 3, ‘Rigour of Development’, resulted in a median score of 13% (range 4–42%). Domain 6, ‘Editorial Independence’, was rated with a median score of 21% (range 0–75%).

**Fig. 3:**
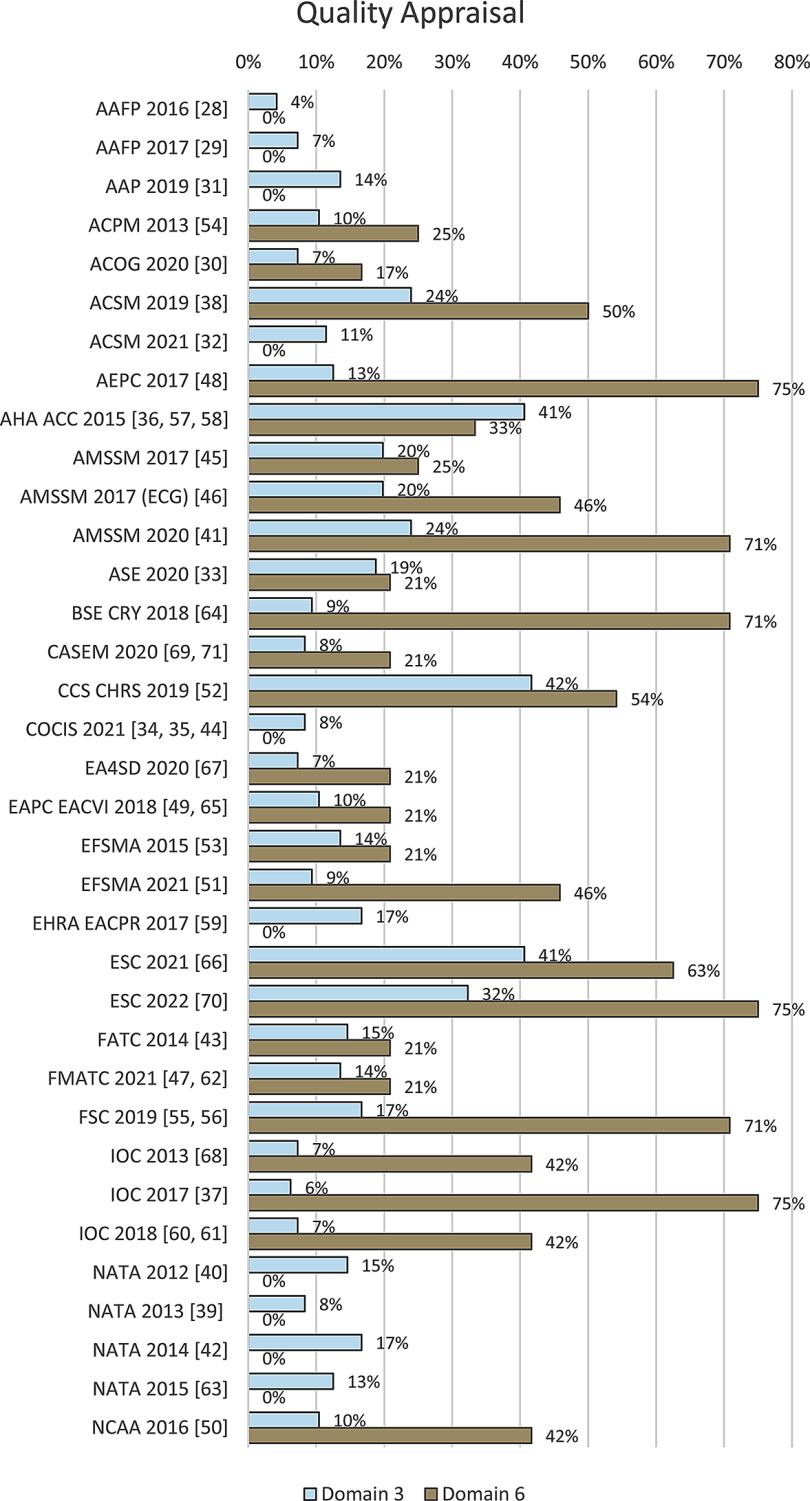
Quality appraisal of included documents (*n* = 35 documents)

Figure 3 depicts the results per document and domain. The detailed quality assessments with reasons are provided as Supplementary Information (Supplement III).

### Key Results

**Fig. 4:**
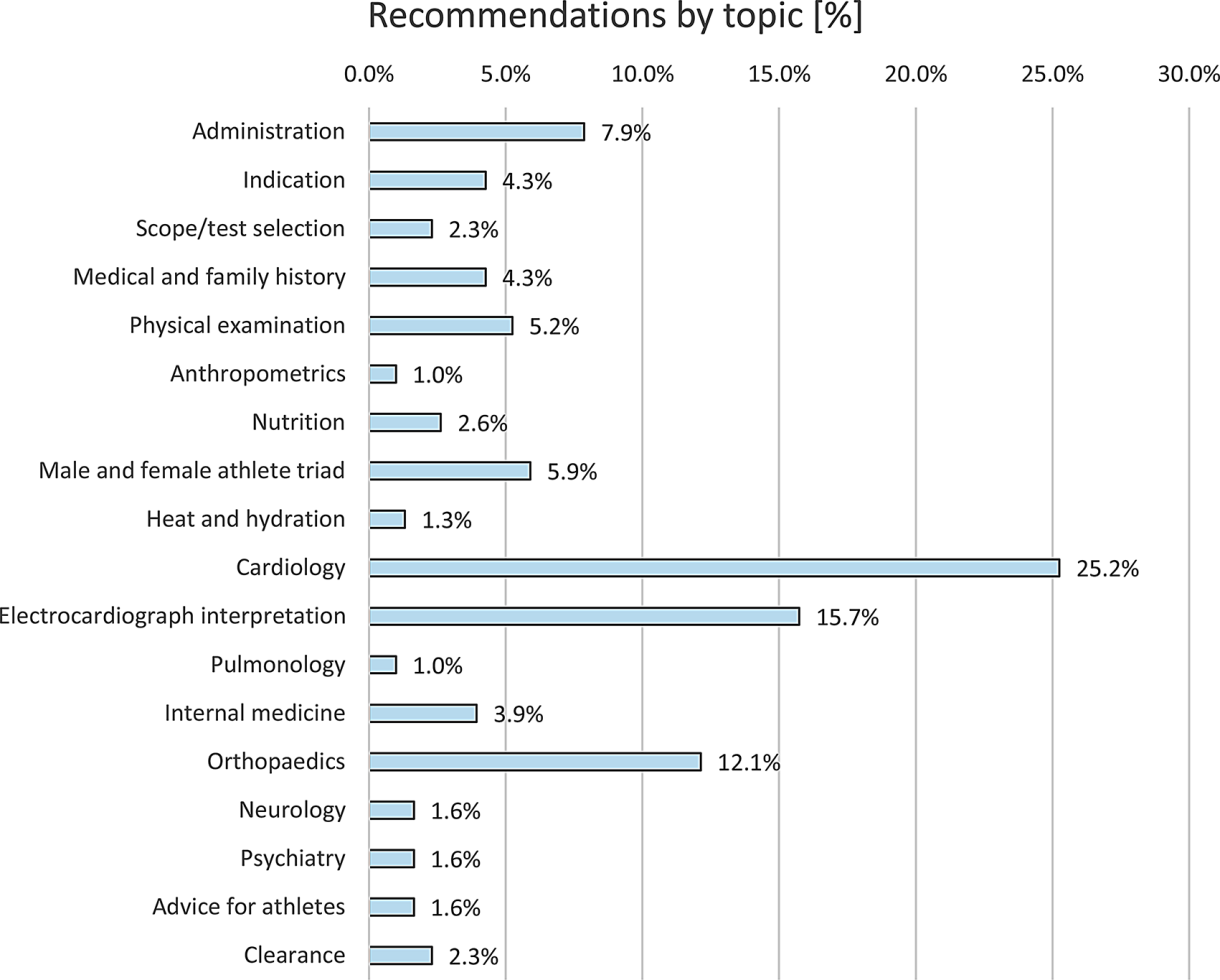
Recommendations by topic (*n* = 305 recommendations)

We extracted 305 recommendations. Of these, 11.8% (36/305) referred to recreational athletes, while 88.2% (269/305) addressed organised sports and/or competitive athletes. The recommendations referred to various topics (Fig. 4). We provide an overview of all recommendations that we extracted, grouped by topic, as Supplementary Information (Supplement IV). Additionally, the full data extraction form is provided as Supplement V.

**Fig. 5:**
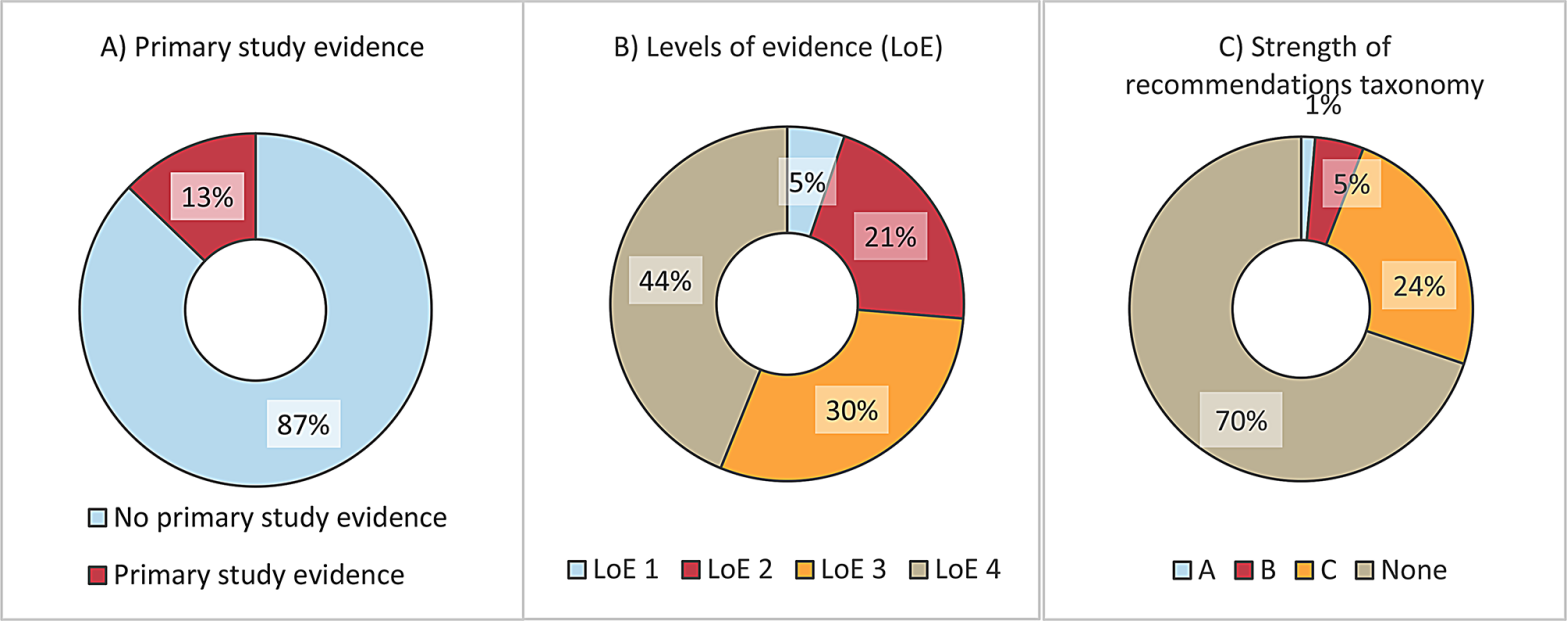
Evidence base and strength of recommendations (**A**) and **C**) *n* = 305 recommendations; **B**) *n* = 39 recommendations with primary study evidence)

A total of 12.8% (39/305) of recommendations were directly linked to evidence from 57 primary studies. The LoEs for those primary studies were distributed as follows: 5.3% (5/57) at LoE1, 21.1% (12/57) at LoE2, 29.8% (17/57) at LoE3 and 43.9% (25/57) at LoE4. In 266 of the 305 recommendations (87.2%), there was no direct link to evidence from primary studies. The strengths of the recommendations according to the SORT were as follows: 1.3% of recommendations (4/305) were rated A, 4.6% (14/305) were rated B and 24.3% (74/305) were rated C. Of the 305 recommendations, 213 (69.8%) were extracted from the text and not explicitly labelled as recommendations by the authors (Fig. 5).

#### Administration

For recreational athletes, we did not identify any recommendations related to PPE administration. Standardisation of PPE was recommended for organised sports and competitive athletes, including (digital) standardised questionnaires and forms [31, 36, 42, 45, 51, 57]. Furthermore, it was recommended that PPE be performed in time to allow for additional evaluations as necessary [31, 42, 45]. Some organisations recommended a complete PPE every two to three years and/or if the level of participation changes, complemented by yearly history taking [31, 42, 48].

In the USA, the American Academy of Pediatrics (AAP) preferred individual examinations [31], while the National Athletic Trainers’ Association (NATA) considered individual and station-based examinations to be equivalent [42]. It was emphasised that previous examination results should be made available to the examiner and that further examinations and specialists need to be accessible [31, 33].

#### Indications for Pre-Participation Evaluation

For recreational athletes, the American College of Sports Medicine (ACSM) recommended in 2021 that the need for PPE should be determined in advance [32] by consulting qualified sports or health professionals or by using the Physical Avidity Readiness Questionnaire Plus (PAR-Q+) [71]. An international collaboration of organisations did not recommend PPE for people who intend to be physically active at light to moderate intensity [69, 72].

In the USA, pregnant women were recommended to undergo PPE to identify possible contraindications to sports during pregnancy [30, 32, 37]. Some organisations also recommended PPE for specific populations of cancer survivors, e.g. those with comorbidities or metastatic disease [32, 38].

#### Scope and Test Selection

For recreational athletes, the ACSM [32] in 2021 recommended laboratory testing ‘depending on individual risk factors, signs, and symptoms’. According to the 2019 AAP recommendations [31], screening tests for organised sports and competitive athletes also depend on findings from the medical history and physical evaluation. In the context of elite athletes, the European Federation of Sports Medicine Associations (EFSMA) [51] recommended thorough PPE, including diverse laboratory and imaging diagnostics, in 2021.

For athletes with disabilities, it was recommended that problems typically related to the disability in question should be monitored [31].

#### Medical and Family History

In the USA and Europe, thorough medical and family history taking was recommended, regardless of the level of participation [32, 42, 51]. This could include past diagnoses or medical procedures, results of physical examinations and laboratory testing, symptoms, illnesses, medications, recreational substance consumption (e.g. alcohol or tobacco), training and work history [32, 51].

There were additional recommendations for specific topics pertinent to athletes with disabilities, e.g. renal problems, devices or assistive equipment, catheterisation, self-care and mobility [31]. For cancer survivors, comorbidities had to be considered, as well as whether the cancer treatment increased the risk of fractures, cardiovascular events, neuropathies or musculoskeletal disorders [32].

#### Physical Examination

Several components of a physical examination can be included in PPE. These components may be related to anthropometrics, internal medicine, neurology, orthopaedics, dermatology, urology, gynaecology, ophthalmology, dentistry, psychiatry or nutrition. Some organisations suggested mandatory physical examinations for the population of interest. These could be supplemented based on the findings of their medical and family history [31, 42].

In pregnant and postpartum women, a supplemental nutritional assessment and an assessment of contraindications were recommended [29]. For athletes with disabilities, it was recommended to examine the skin for harm due to friction, shearing or pressure from a wheelchair or other assistive devices and to check bladder catheters [31].

#### Anthropometrics

All recommendations for anthropometrics applied to organised sports and competitive athletes. In 2019, the AAP [31] recommended measuring height, weight and body mass index (considering the higher muscle mass of some athletes) to diagnose underweight and overweight. In children, growth curves were recommended to be used [31]. For European elite athletes, the EFSMA [51] complemented these measures with somatoscopy and other body measurements, as well as mobility and strength, in their 2021 recommendations.

#### Nutrition

We did not identify any nutrition-related recommendations for recreational athletes. All recommendations addressed organised sports and competitive athletes. These were related to energy-balanced eating, as well as disordered eating and eating disorders (DE/ED). It was recommended to include DE/ED in routine assessments [29, 41, 60, 68]. The International Olympic Committee (IOC) provided a risk assessment model for this context [60]. If DE/ED is suspected, an ‘Anthropometric, Biochemical, Clinical, Dietary and Environmental (ABCDE) Assessment’ was recommended for further investigation [60]. Other screening tools mentioned by the American Academy for Family Physicians (AAFP) in 2017 [29] were SCOFF questions [73], Eating Disorder Inventory (EDI) [74] and Low Energy Availability in Females Questionnaire (LEAF-Q) [75].

For elite athletes, the EFSMA [51] specifically recommended a comprehensive nutritional assessment in 2021.

#### Male and Female Athlete Triads

For female participants in organised sports and competitive athletes, screening for a triad is recommended as part of PPE in the USA [29, 43]. For male athletes, targeted screening questions were also recommended [47, 62]. Screening for triads should begin at school age and should include a medication history (particularly hormones) [43]. Any identified element of the female athlete triad (underweight, amenorrhea or decreased bone density) should prompt further investigation for the presence of the other elements [31, 43]. Further investigation was recommended for abnormal menstruation [42] or underweight [31]. European guidelines and consensus documents did not address the male or female athlete triad, however.

#### Heat and Hydration

History questions about heat illness and risk factors, including fluid intake, training intensity, acclimatisation and screening for the sickle cell trait, were recommended for competitive athletes and in organised sports in the USA [28, 31, 40, 42].

#### Internal Medicine

In general, cardiologic recommendations included a cardiac medical and family history and a physical examination for all athletes. Recommendations for further cardiologic investigations (e.g. electrocardiography [ECG]) varied according to participation level, age and other risk factors, as well as region (e.g. USA vs. Europe). For example, in 2021, the European Society of Cardiology (ESC) [66] recommended stratifying cardiologic assessments for people > 35 years of age according to their individual cardiovascular risk, which should be evaluated using the SCORE2 (Systematic COronary Risk Evaluation 2) instrument [76]. According to the ACSM recommendations of 2019 [38], all cancer survivors should be screened for cardiovascular disease and receive a cardiopulmonary exercise test, if deemed necessary. The ESC [66] recommended in 2021 that cancer survivors who received cardiotoxic therapies should undergo echocardiography before exercising at high intensity.

In the context of organised sports and competitive athletes, recommendations for ECG as a baseline cardiologic examination were inconsistent. In Europe, a 12-lead ECG was generally recommended [44, 48, 53, 64]. In North America, a positive (family) history and/or physical examination was sometimes required [39, 52, 54] and, in some cases, a decision was made depending on resources [33], or a shared decision-making approach was chosen [45].

Further cardiologic recommendations addressed the interpretation of cardiac imaging in athletes and can be found in the Supplementary Information (Supplement IV).

In the context of pneumological screening, no specific recommendations could be identified for recreational athletes. For organised sports in the USA, a thorough history taking and physical examination were recommended when asthma is suspected [40].

Except in the 2021 EFSMA recommendations for elite athletes [51], specific blood and urine tests were not routinely recommended but could be considered depending on risk factors and findings from medical history and physical examinations [31, 42].

#### Orthopaedics

We did not identify any orthopaedic recommendations for recreational athletes. For organised sports and athletes of all levels, it was recommended to start with a history of injuries and surgeries, as well as a physical examination [31, 42, 51]. The results of these examinations could then be used to determine whether further diagnostic assessments were necessary [31, 42, 51].

For female athletes, particular attention should be paid to risks for anterior cruciate ligament injuries, patellofemoral pain and musculoskeletal deficits [29]. Recommendations for athletes with disabilities included an examination of the stability, flexibility and strength of stressed and frequently injured sites [31].

#### Neurology

There were no neurological recommendations for recreational athletes. For organised sports and elite athletes, a thorough neurological examination was recommended for athletes with a history of concussion, seizures, cervical stenosis or spinal cord injury [42, 51]. The AAFP [28] recommended in 2016 that all athletes undergo a neurological and cervical spine examination to prevent cervical spine injuries.

According to the AAP recommendations of 2019 [31], athletes with physical impairments should receive a complete neurological assessment. In the same year, the ACSM [38] recommended an assessment of balance and mobility for older cancer survivors and those who received neurotoxic chemotherapy.

#### Psychiatry

Specific screening recommendations for psychiatric issues were not available for recreational athletes. For participants in organised sports and athletes, it was recommended to supplement medical history taking with questions about mental health [31, 42, 63].

#### Advice To Participants

As part of PPE, some organisations recommended counselling sports participants about health risks and preventive measures [31, 45, 48]. For recreational athletes, the ACSM [32] recommended in 2021 that “pregnant women should be educated on the warning signs for when to stop exercise”.

#### Clearance

In its 2019 recommendations, the AAP [31] provided a list of key questions practitioners should consider (Table 4).

**Table 4:** American academy of pediatrics’ recommendations for sports participation clearance

American Academy of Pediatrics’ recommendations for sports participation clearance [31]	
• Does participation put the athlete at risk for illness or injury above the inherent hazards of the activity?	
• Does participation increase the risk of injury or illness for other participants?	
• Will treatment of the underlying condition allow safe participation (medication, rehabilitation, bracing and padding)?	
• Can limited participation be allowed while treatment or evaluation is completed?	
• If medical eligibility is denied for certain sports because of medical or safety concerns, can the athlete safely participate in other activities or sports?	

Moreover, cardiovascular abnormalities should be further evaluated before starting or continuing high-intensity exercise [31]. Further details were provided in specialist guidelines for cardiovascular disease in athletes, but these were beyond the scope of this review. In the case of athlete triads or relative energy deficiency in sport (REDs), the use of risk assessment tools was recommended to guide clearance decisions [47, 60, 62]. For athletes with disabilities, inclusion should be the primary consideration, along with safety issues [31].

## Discussion

This review identified numerous recommendations for performing PPE in sports medicine. Most of them were directed at participants in organised sports or competitive athletes, while fewer recommendations addressed PPE of recreational athletes. These recommendations were also often limited to specific subgroups, such as pregnant women or cancer survivors, and did not cover important topics that affect injury prevention, such as orthopaedics or nutrition [77, 78]. There was only one guideline that included specific recommendations for people with disabilities [31], despite the specific requirement of this population for recommendations on safely performing sports and already existing standardised evaluation tools [79].

Most organisations agreed that essential components of PPE include thorough medical and family history taking and a physical examination in all populations. Several organisations recommended a stepwise approach to PPE in which follow-up questions and examinations are chosen based on the results of the mandatory history taking and physical evaluations [31, 42].

Varying and sometimes even contradictory recommendations existed for blood and urine testing, exercise stress testing and imaging, especially in the context of cardiovascular PPE. An example is the controversial use of ECG in North American versus European organisations. In North America, the use of ECG screening is more restricted than in Europe and is usually dependent on specific conditions (e.g. risk factors identified by medical or family history) [33, 39, 44, 45, 48, 52–54, 64].

Only a few recommendations were based on evidence; most recommendations seemed to be derived largely from expert experience and consensus. This is not surprising, as robust evidence for the positive effects of PPE on patient-relevant health outcomes is scarce [52, 80]. The lack of evidence and reliance on consensus probably contributed to the heterogeneity among recommendations from different organisations and regions. Methodological and organisational challenges in the design and conduct of screening trials also suggest that prospective high-quality studies will continue to be limited [81–83].

The lack of evidence on which to base recommendations was the main driver for the poor ratings of the included documents in the ‘Rigour of Development’ domain of the AGREE II tool. This is consistent with the findings of Riding et al., who systematically reviewed guidelines for cardiovascular PPE [84]. Riding et al. found that ‘Rigour of Development’ scored lower than any of the other AGREE II domains. According to the authors, the poor-quality appraisal scores of guidelines in preventive sports medicine are attributable more to the limitations in this area of research than to the rigour applied by the guideline groups [84]. In addition to limited underlying evidence, we perceived poor reporting to be another concern. Both AGREE II domains were given lower ratings due to the lack of available information on guideline methodology, funding and conflict of interest management. Therefore, the use of standardised methods and guidance for the development and reporting of guidelines (e.g. the Reporting Items for practice Guidelines in HealThcare) [85] is desirable.

The lack of solid evidence equally applies to the potential harms of PPE [81], which need to be discussed to provide a balanced overview. One such harm was proposed to be psychological distress in athletes caused by true-positive or false-positive results [81]. Hill et al. conducted an SR of the psychological distress of athletes caused by cardiovascular PPE [86]. While their study showed that PPE generally caused only minimal or no psychological distress to athletes and made them feel safer, a few athletes with true-positive findings did experience distress. According to the authors, this may have been related to follow-up evaluations, sports restrictions or disqualifications [86]. However, psychological distress affecting a minority of people screened can be justified if positive health outcomes are likely achieved through PPE and appropriate follow-up measures. Another harm of PPE occurs if it is perceived as a barrier to performing sports and even more people remain sedentary. Therefore, it is important to see PPE as an opportunity to allay potential fears (e.g. ‘Can I do sports with my knees?’) and find good advice ('Which sport is suitable for me and which should I avoid?’), rather than as a duty that prevents people from being active.

The decisions surrounding which components to include in PPE may be seen as health decisions, for which evidence for the superiority of one intervention (to screen) over another (not to screen) is either not available or does not allow for differentiation [87]. In this context, the best choice depends on how individuals value the risks and benefits of the interventions, and shared decision-making about the scope of PPE should be the norm.

Despite the uncertainties associated with its benefits and harms, PPE may be a tool for ensuring that current health problems are managed appropriately and for determining whether a person is medically able to engage in a particular sport [88].

### Implications of the Research

More robust evidence for the effects of PPE on health outcomes is needed. Studies on preventive health examinations that aim to collect patient-relevant outcomes face particular methodological challenges associated with randomisation, large sample sizes and long-term follow-up [89]. Large cluster-randomised trials [90, 91], registry-based studies or national cohorts may be the best approach to obtain robust evidence in this context. This might include population-based registries for fatal events or sports-related injuries, analysis of data from health insurance providers or the prospective collection and evaluation of data on preventive health examinations, including follow-up examinations.

### Limitations

This SR has several limitations. First, we included only documents published in English and German. Additionally, we did not perform forward citation screening due to resource restrictions. Therefore, potentially relevant documents not covered by our search strategy as well as literature published in other languages were not included. Second, many recommendations were extracted directly from the text, as not all documents included recommendations labelled as such. We felt it was important to include these documents and extract recommendations from the text due to their coverage of highly relevant topics and their language suggesting that recommendations were being provided. To mitigate the subjectivity of this process, we performed thorough quality assurance of extractions. Third, due to resource restrictions, we applied only domains 3 and 6 of the AGREE II tool to quality appraisal and were unable to provide information about the other domains. However, domains 3 and 6 contain the most informative items for assessing the underlying methods and evidence used to develop recommendations, as well as the potential effects of funding or conflicts of interest. Therefore, we expect that this omission probably did not substantially affect the results or conclusions of this SR. This is supported by a survey of guideline and AGREE II users, which concluded that domains 3 and 6 had the strongest influence on the overall assessment of guideline quality and recommendations for use [92]. Fourth, we did not include evidence from primary studies in this SR. While this was not the aim of our review, a future review is required to summarise primary research evidence in this field and provide a more solid foundation for future guidelines.

## Conclusion

Our review identified recommendations for most components of PPE, ranging from indications and scope to individual diagnostic tests. They helped to define the scope of and clinical questions for the PPE guideline currently being developed in Germany. Most recommendations identified in this review addressed competitive athletes, so there is a need for a comprehensive set of recommendations for individuals who exercise in a recreational setting and recommendations addressing the specific needs of people with disabilities performing sports.

Recommendations for the components of PPE were heterogeneous across organisations and geographic regions and were rarely based on evidence from comparative studies. Therefore, more robust evidence for the effects of PPE on health outcomes, e.g. from large cluster-randomised trials or cohort and registry studies, is needed.

Reporting should be improved for future guidelines and consensus statements, as well as structured searches and use of primary data evidence for the development of recommendations. In addition, both the potential benefits and harms of PPE should be considered and the preferences of the target population should be taken into account.

## Electronic supplementary material

Below is the link to the electronic supplementary material.

Supplementary Material 1

Supplementary Material 2

Supplementary Material 3

Supplementary Material 4

Supplementary Material 5

## Data Availability

All data are available within the paper or its supplements.

## Abbreviations

ABCDE: Anthropometric, Biochemical, Clinical, Dietary and Environmental (Assessment)
AAFP: American Academy of Family Physicians
AAP: American Academy of Pediatrics
AAOS: American Academy of Orthopaedic Surgeons
AAPMR: American Academy of Physical Medicine and Rehabilitation
AASP: Association of Applied Sport Psychiatry
ACC: American College of Cardiology
ACC SECLC: American College of Cardiology Sports and Exercise Cardiology Leadership Council
ACEP: American College of Emergency Physicians
ACOG: American College of Obstreticians and Gynecologists
ACS: American Cancer Society
ACLM: American College of Lifestyle Medicine
ACRM: American Congress of Rehabilitation Medicine
ACSM: American College of Sports Medicine
ACSEP: Australasian College of Sports and Exercise Physicians
AEPC: Association of European Paediatric Cardiology
AGREE: Appraisal of Guidelines for Research and Evaluation
AHA: American Heart Association
AIS: Australian Institute of Sport
AMSSM: American Medical Society for Sports Medicine
AOASM: American Osteopathic Academy of Sports Medicine
AOSSM: American Orthopaedic Society for Sports Medicine
APA: American Psychological Association
APHRS: Asia Pacific Heart Rhythm Society
APTA: American Physical Therapy Association
ASCA: American School Counselor Association
ASD: Academy for Sports Dentistry
ASE: American Society of Echocardiography
ASSMP: Austrian Society. of Sports Medicine and Prevention
BASEM: British Association for Sports and Exercise Medicine
BSE CRY: British Society of Echocardiography and Cardiac Risk in the Young
CACHN: Community and Athletic Cardiovascular Health Network
CARF: Commission on Accreditation of Rehabilitation Facilities
CASEM: Canadian Academy of Sport and Exercise Medicine
CATA: Canadian Athletic Therapists Association
CATS: College Athletic Trainers’ Society
CCS: Canadian Cardiovascular Society
CDC: Centers for Disease Control
CHRS: Canadian Heart Rhythm Society
CIHR: Canadian Institute of Health Research
COC: Canadian Olympic Committee
COPSI: Canadian Olympic and Paralympic Sport Institute Network
CPC: Canadian Paralympic Committee
CSCCA: Collegiate Strength and Conditioning Coaches Association
CSEP: Canadian Society for Exercise Physiology
CSI: Canadian Sport Institute
DVGS: Deutscher Verband für Gesundheitssport und Sporttherapie
EA4SD: European Association for Sports Dentistry
EACPR: European Association for Cardiovascular Prevention and Rehabilitation
EACVI: European Association of Cardiovascular Imaging
ECG: electrocardiography
ECSEP: European College of Sports and Exercise Physicians
EDI: Eating Disorder Inventory
EFSMA: European Federation of Sports Medicine Associations
EHRA: European Heart Rhythm Association
EAPC: European Association of Preventive Cardiology
ESC: European Society of Cardiology
ESSA: Exercise and Sports Science Australia
FATC: Female Athlete Triad Coalition
FMATC: Female and Male Athlete Triad Coalition
FIFA: Fédération Internationale de Football Association
GIN: Guidelines International Network
HRS: Heart Rhythm Society
HSF: Heart and Stroke Foundation
IAEHC: Italian Association of Extra-hospital Cardiologists
IAIHC: Italian Association of In-hospital Cardiologists
ICISF: International Critical Incident Stress Foundation
IOC: International Olympic Committee
ISC: Italian Society of Cardiology
ISSP: International Society of Sports Psychiatry
LEAF-Q: Low Energy Availability in Females Questionnaire
LoE: Level of Evidence
NAIAAA: National Interscholastic Athletic Administrators Association
NASMPA: Norwegian Association of Sports Medicine and Physical Activity
NATA: National Athletic Trainers’ Association
NCAA: National Collegiate Athletics Association
NCCN: National Comprehensive Cancer Network
NCI: National Cancer Institute
NCSF: National Council on Strength and Fitness
NFHS: National Federation of State High School Associations
NIH: National Institutes of Health
NSCA: National Strength and Conditioning Association
PAR-Q+: Physical Activity Readiness Questionnaire Plus
PERSiST: Implementing Prisma in Exercise, Rehabilitation, Sport medicine and SporTs science
PICO: Population, Intervention, Comparison, Outcome
PRISMA: Preferred Reporting Items for Systematic Reviews and Meta-Analyses
PPE: pre-participation evaluation
RDSPT: Royal Dutch Society for Physical Therapy
REDs: Relative Energy Deficiency in Sports
SASMA: South African Sports Medicine Association
SBC-DERC: Brazilian Society of Cardiology – Department of Exercise and Rehabilitation
SBM: Society for Behavioral Medicine
SCCT: Society of Cardiovascular Computed Tomography
SCG: Sports Cardiology Group
SCMR: Society for Cardiovascular Magnetic Resonance
SCOFF: SCOFF
SCORE2: Systematic COronary Risk Evaluation 2
SDA: Sports Doctors Australia
SGS: Schweizerische Gesellschaft für Sportmedizin
SOLAECE: Sociedad Latino Americana de Estimulacion Cardiaca y Electrofisiologia
SORT: Strength of Recommendations Taxonomy
SR: Systematic Review
SSC: Spanish Society of Cardiology
SSESM: Swedish Society of Exercise and Sports Medicine
WHO: World Health Organization

## Footnotes

1 These are henceforth referred to as ‘documents’.
