# Supplementary material for: Pre-Participation Evaluation of Recreational and Competitive Athletes – A Systematic Review of Guidelines and Consensus Statements
Source: Sports Med Open. 2025 Apr 5;11:33. doi: 10.1186/s40798-025-00837-6 (PMC11972279; doi:10.1186/s40798-025-00837-6)
Supplement: Supplementary file 1 — Supplementary Material 1 [file 40798_2025_837_MOESM1_ESM.pdf]

Cover page

Supplement I: Systematic Search

Article title: Pre-participation evaluation of recreational and competitive athletes – A systematic review of guidelines and consensus statements

Journal: Sports Medicine - Open

Authors: Alina Weise, Nadja Könsgen, Christine Joisten, Fabian Schlumberger, Anja Hirschmüller, Jessica Breuing, Käthe Gooßen

Corresponding author: Dr Alina Weise, Witten/Herdecke University, Institute for Research in Operative Medicine (IFOM), Cologne, Germany. ORCID: https://orcid.org/0000-0003-4563-5782, alina.weise@uni-wh.de

Supplement I: Systematic Search

Database searches

Medline via Pubmed (date: 09-08-2022)

Search number	Query	Search Details	Results
**6**	#4 AND #5	("athlet*"[Title/Abstract] OR "sport*"[Title/Abstract] OR "physical activ*"[Title/Abstract] OR "non athlet*"[Title/Abstract] OR "Sports"[MeSH Terms] OR "Athletes"[MeSH Terms] OR "Exercise"[MeSH Terms]) AND ("pre-participation"[Title/Abstract] OR "pre-participation"[Title/Abstract] OR "preparticipation"[Title/Abstract] OR "exercise test*"[Title/Abstract] OR "physical examination*"[Title/Abstract] OR "clinical examination*"[Title/Abstract] OR "physical evaluation*"[Title/Abstract] OR "clinical evaluation*"[Title/Abstract] OR "diagnostic examination*"[Title/Abstract] OR "diagnostic evaluation*"[Title/Abstract] OR "clinical assessment*"[Title/Abstract] OR "physical assessment*"[Title/Abstract] OR "diagnostic assessment*"[Title/Abstract] OR "prevention"[Title/Abstract] OR "Diagnosis"[MeSH Terms] OR "death, sudden, cardiac/prevention and control"[MeSH Terms] OR "athletic injuries/prevention and control"[MeSH Terms] OR "relative energy deficiency in sport/diagnosis"[MeSH Terms] OR "relative energy deficiency in sport/prevention and control"[MeSH Terms]) AND ("guideline"[Publication Type] OR "Guidelines as Topic"[MeSH Terms] OR "practice guideline"[Publication Type] OR "Consensus"[MeSH Terms] OR "consensus development conference, nih"[Publication Type] OR "Consensus Development Conference"[Publication Type] OR "consensus*"[Title] OR "position statement*"[Title] OR "guideline*"[Title] OR "recommend*"[Title] OR "guidance"[Title]) AND 2012/01/01:3000/12/31[Date - Publication]	2,245
**5**	("2012/01/01"[Date - Publication] : "3000"[Date - Publication])	2012/01/01:3000/12/31[Date - Publication]	12,806,307

**4**	#1 AND #2 AND #3	("athlet*"[Title/Abstract] OR "sport*"[Title/Abstract] OR "physical activ*"[Title/Abstract] OR "non athlet*"[Title/Abstract] OR "Sports"[MeSH Terms] OR "Athletes"[MeSH Terms] OR "Exercise"[MeSH Terms]) AND ("pre-participation"[Title/Abstract] OR "pre-participation"[Title/Abstract] OR "preparticipation"[Title/Abstract] OR "exercise test*"[Title/Abstract] OR "physical examination*"[Title/Abstract] OR "clinical examination*"[Title/Abstract] OR "physical evaluation*"[Title/Abstract] OR "clinical evaluation*"[Title/Abstract] OR "diagnostic examination*"[Title/Abstract] OR "diagnostic evaluation*"[Title/Abstract] OR "clinical assessment*"[Title/Abstract] OR "physical assessment*"[Title/Abstract] OR "diagnostic assessment*"[Title/Abstract] OR "prevention"[Title/Abstract] OR "Diagnosis"[MeSH Terms] OR "death, sudden, cardiac/prevention and control"[MeSH Terms] OR "athletic injuries/prevention and control"[MeSH Terms] OR "relative energy deficiency in sport/diagnosis"[MeSH Terms] OR "relative energy deficiency in sport/prevention and control"[MeSH Terms]) AND ("guideline"[Publication Type] OR "Guidelines as Topic"[MeSH Terms] OR "practice guideline"[Publication Type] OR "Consensus"[MeSH Terms] OR "consensus development conference, nih"[Publication Type] OR "Consensus Development Conference"[Publication Type] OR "consensus*"[Title] OR "position statement*"[Title] OR "guideline*"[Title] OR "recommend*"[Title] OR "guidance"[Title])	3,820
**3**	("guideline"[Publication Type] OR "Guidelines as Topic"[MeSH Terms] OR "practice guideline"[Publication Type] OR "Consensus"[MeSH Terms] OR "consensus development conference, nih"[Publication Type] OR "Consensus Development Conference"[Publication Type] OR "consensus*"[Title] OR "position statement*"[Title] OR "guideline*"[Title] OR "recommend*"[Title] OR "guidance"[Title])	"guideline"[Publication Type] OR "Guidelines as Topic"[MeSH Terms] OR "practice guideline"[Publication Type] OR "Consensus"[MeSH Terms] OR "consensus development conference, nih"[Publication Type] OR "Consensus Development Conference"[Publication Type] OR "consensus*"[Title] OR "position statement*"[Title] OR "guideline*"[Title] OR "recommend*"[Title] OR "guidance"[Title]	330,293

**2**	("pre participation"[Title/Abstract] OR "pre-participation"[Title/Abstract] OR "preparticipation"[Title/Abstract] OR "exercise test*"[Title/Abstract] OR "physical examination*"[Title/Abstract] OR "clinical examination*"[Title/Abstract] OR "physical evaluation*"[Title/Abstract] OR "clinical evaluation*"[Title/Abstract] OR "diagnostic examination*"[Title/Abstract] OR "diagnostic evaluation*"[Title/Abstract] OR "clinical assessment*"[Title/Abstract] OR "physical assessment*"[Title/Abstract] OR "diagnostic assessment*"[Title/Abstract] OR "prevention"[Title/Abstract] OR "Diagnosis"[MeSH Terms] OR "death, sudden, cardiac/prevention and control"[MeSH Terms] OR "athletic injuries/prevention and control"[MeSH Terms] OR "Relative Energy Deficiency in Sport/diagnosis"[Mesh] OR "Relative Energy Deficiency in Sport/prevention and control"[Mesh])	"pre-participation"[Title/Abstract] OR "pre-participation"[Title/Abstract] OR "preparticipation"[Title/Abstract] OR "exercise test*"[Title/Abstract] OR "physical examination*"[Title/Abstract] OR "clinical examination*"[Title/Abstract] OR "physical evaluation*"[Title/Abstract] OR "clinical evaluation*"[Title/Abstract] OR "diagnostic examination*"[Title/Abstract] OR "diagnostic evaluation*"[Title/Abstract] OR "clinical assessment*"[Title/Abstract] OR "physical assessment*"[Title/Abstract] OR "diagnostic assessment*"[Title/Abstract] OR "prevention"[Title/Abstract] OR "Diagnosis"[MeSH Terms] OR "death, sudden, cardiac/prevention and control"[MeSH Terms] OR "athletic injuries/prevention and control"[MeSH Terms] OR "relative energy deficiency in sport/diagnosis"[MeSH Terms] OR "relative energy deficiency in sport/prevention and control"[MeSH Terms]	9,795,115
**1**	("athlet*"[Title/Abstract] OR "sport*"[Title/Abstract] OR "physical activ*"[Title/Abstract] OR "non-athlet*"[Title/Abstract] OR "Sports"[MeSH Terms] OR "Athletes"[MeSH Terms] OR "Exercise"[MesH Terms])	"athlet*"[Title/Abstract] OR "sport*"[Title/Abstract] OR "physical activ*"[Title/Abstract] OR "non athlet*"[Title/Abstract] OR "Sports"[MeSH Terms] OR "Athletes"[MeSH Terms] OR "Exercise"[MeSH Terms]	483,632

ECRI (https://www.ecri.org/): Search date: 11-08-2022, In total: 42 Records

- athlete/athletes + Filter: Guidance: 2 Records
- sport + Filter Guidance: 2 Records
- sports + Filter Guidance: 7 Records
- physically active + Filter Guidance: 26 Records
- preparticipation: 0 Records
- pre-participation + Filter Guidance: 5 Records

GIN (https://guidelines.ebmportal.com/): Search date: 09-08-2022, In total: 16 Records, thereof 4 were published before 2012 and therefore not screened

- athlet*: 1 Record
- sport*: 3 Records
- physically activ*/physical activity: 12 Records

- pre participation/pre-participation/preparticipation: 0 Records

NIH Library (https://www.nihlibrary.nih.gov/): Search date: 10-08-2022, Records: 114, all were duplicates and therefore not screened

- Titel contains: ("athlet*" OR "sport*"OR "physical activ*"OR "non-athlet*") AND ("pre participation" OR "pre-participation"OR "preparticipation" OR "exercise test*" OR "physical examination*" OR "clinical examination*"OR "physical evaluation*" OR "clinical evaluation*" OR "diagnostic examination*"OR "diagnostic evaluation*"OR "clinical assessment*" OR "physical assessment*" OR "diagnostic assessment*" OR "prevention") AND ("consensus*" OR "position statement*" OR "guideline*" OR "recommend*" OR "guidance")

TRIP Database (https://www.tripdatabase.com/): Search date: 09-08-2022, Records: 13 thereof 4 were published before 2012 and therefore not screened

- Population: athlete OR athletes OR athletic OR sport OR sports OR "physically active" OR "physical activity"Intervention: "pre participation" OR preparticipation OR diagnostic OR diagnosis OR "exercise test" OR "clinical examination" OR "physical evaluation" OR "clinical evaluation" OR "physical examination" OR "clinical assessment" OR "physical assessment" OR prevention
  Filter: Guidelines

Website searches

American College of Sports Medicine (https://www.acsm.org/): Search date: 09-08-2022, In total: 28 Records

- ACSM's Resource Library, Filter “Publication” Date range 01/01/2012-09/08/2022: 13 Records
- ACMS’s Books: 15 Records

British Association of Sport & Exercise Medicine (https://basem.co.uk/): Search date: 23-08-2022, In total: 15 Records thereof two were duplicates and therefore mnot screened

- SEM Resources, Covid-19 (guideline, consensus statement): 2 Records
- SEM Resources, Para-sport Classification Lexi (guideline, consensus statement): 0 Records
- SEM Resources, Mental health resources (guideline, consensus statement): 4 Records
- SEM Resources, MSK Resources for junior clinicians (guideline, consensus statement): 0 Records
- SEM Resources, Harassment in sport (guideline, consensus statement): 3 Records
- SEM Resources, Return to play (guideline, consensus statement): 2 Records
- SEM Resources, BJSM (guideline, consensus statement): 0 Records
- SEM Resources, BASEM today (guideline, consensus statement): 0 Records
- SEM Resources, RED-S (guideline, consensus statement): 1 Record
- SEM Resources, anti-doping (guideline, consensus statement): 1 Record
- SEM Resources, female athlete health (guideline, consensus statement): 2 Records
- SEM Resources, orthoevidence (guideline, consensus statement): 0 Records

Canadian Academy of Sport and Excercise Medicine (https://casem-acmse.org/): Search date: 10-08-2022, In total: 81 Records thereof 10 were marked as outdated, explicitly marked as podcast or evaluation too, or published before 2012 and therefore not screened

- Position statements: 20 Records
- Concussion resources: 27 Records
- Resident/Fellow SEM Resources, text books: 6 Records
- Resident/Fellow SEM Resources, Cardiology/Preparticipation physical exam (PPE): 8 Records
- Resident/Fellow SEM Resources, Dermatology: 2 Records
- Resident/Fellow SEM Resources, Orthopedics: 3 Records
- Resident/Fellow SEM Resources, Para and adaptive sport: 2 Records
- Resident/Fellow SEM Resources, RED-S: 1 Record
- Covid-19 resources, Sport Medicine Advisory Committee, tools to assist in the decision making: 6 Records
- Covid-19 resources, other resources: 4 Records

Sports Medicine Australia (https://sma.org.au/): Search date: 10-08-2022, In total: 14 Records thereof two were duplicates and therefore not screened

- Resources & Advice, Policies and Guidelines: 10 Records
- Resources & Advice, SMA Position Statements: 4 Records

Eurpoean Federation of Sports Medicine Associations (https://www.efsma.org/): Search date: 25-10-2022, In total: 61 Records

- News & Events: Publications (61 Records)

Canadian Medical Association Infobase of Clinical Practice Guidelines (https://joulecma.ca/cpg/homepage): Search date: 10-08-2022, Records: 21

- Suchstring: athlet* OR sport* OR "physically active" OR "physical activity" OR "pre participation" OR preparticipation

Australian National Health and Medical Research Council (https://www.nhmrc.gov.au/), Search date: 10-08-2022, Records: 45 thereof 17 were published before 2012 and therefore not screened

- Suchstring: athlet* OR sport* OR "physically active" OR "physical activity" OR "pre participation" OR preparticipation + Filter: Guideline

National Institute for Health and Care Excellence (https://www.nice.org.uk/): Search date: 10-08-2022, In total: Records: 8

- Suchstring: athlete OR athletes OR sport OR sports OR "physically active" OR "physical activity" OR "pre participation" OR preparticipation, Filter: Guidance, Last updated date between01/1/2012-10/8/2022

New Zealand Guidelines Group via Ministry of Health New Zealand (https://www.health.govt.nz/) : Search date: 10-08-2022, In total: 25 Records thereof 4 were published before 2012 and therefore not screened

- Athlete/athletes: 0 Records
- Sport/sports, Filter: Guides and standards: 2 Records
- Physically active/physical activity, Filter: Guides and standards, 21 Records
- Preparticipation: 0 Records
- Pre participation: 2 Records

Scottish Intercollegiate Guidelines Network (https://www.sign.ac.uk/): Search date: 23-08-2022, Records: 42 thereof 1 was published before 2012 and therefore not screened

- Our Guidelines, current guidelines

VA/DoD Clinical Practice Guidelines (https://www.healthquality.va.gov/): Search date: 23.08.2022, Records: 22

- VA/DoD Clinical Practice Guidelines

Backward citation screening

Scopus Advanced Search: Search date: 13-12-2022

- DOI (10.1016/j.acvd.2018.05.005) OR DOI (10.1016/j.acvd.2018.07.001) OR DOI (10.1016/j.cjca.2018.10.016) OR DOI (10.1016/j.cjca.2020.11.007) OR DOI (10.1016/j.echo.2020.02.009) OR DOI (10.1016/j.jsams.2020.05.004) OR DOI (10.1017/s1047951117001305) OR DOI (10.1017/s1047951117001986) OR DOI (10.1017/s1047951117002700) OR DOI (10.1093/ehjci/jeu323) OR DOI (10.1093/eurheartj/ehac262) OR DOI (10.1093/eurheartj/ehv316) OR DOI (10.1093/eurheartj/ehw631) OR DOI (10.1093/eurheartj/ehx532) OR DOI (10.1093/eurheartj/ehy408) OR DOI (10.1093/eurheartj/ehy730) OR DOI (10.1097/jsm.0000000000000664) OR DOI (10.1097/jsm.0000000000000892) OR DOI (10.1097/jsm.0000000000000946) OR DOI (10.1097/jsm.0000000000000948) OR DOI (10.1097/jsm.0000000000000981) OR DOI (10.1111/edt.12593) OR DOI (10.1123/ijsnem.2018-0136) OR DOI (10.1136/bjsports-2013-092966) OR DOI (10.1136/bjsports-2013-093218) OR DOI (10.1136/bjsports-2014-093502) OR DOI (10.1136/bjsports-2016-096781) OR DOI (10.1136/bjsports-2016-097331) OR DOI (10.1136/bjsports-2018-099193) OR DOI (10.1136/bjsports-2018-099351) OR DOI (10.1136/bjsports-2019-101583) OR DOI (10.1136/bjsports-2019-101813) OR DOI (10.1136/bmjsem-2021-001178) OR DOI (10.1161/cir.0000000000000236) OR DOI (10.1161/cir.0000000000000238) OR DOI (10.1161/cir.0000000000000242) OR DOI (10.1177/2047487316676042) OR DOI (10.1177/19417381221077138) OR DOI (10.1249/jsr.0000000000000231) OR DOI (10.1249/mss.0b013e318279a10a) OR DOI (10.1249/mss.0000000000000664) OR DOI (10.1249/mss.0000000000002116) OR DOI (10.1530/erp-17-0075) OR DOI (10.2459/jcm.0b013e32835f6a21) OR DOI (10.2459/jcm.0b013e32835fcb8a) OR DOI (10.4085/1062-6050-47.1.96) OR DOI (10.4085/1062-6050-48.4.12) OR DOI (10.4085/1062-6050-48.6.05) OR DOI (10.4085/1062-6050-50.3.03) OR DOI (10.4085/j.jacc.2016.03.527)
- 54 document results
  → View references (all 54 selected)
- 3817 references cited by 54 selected documents
- Filter: 2012-2022
  → 1396 references cited by 54 selected documents
- Duplicate removal according to previously screened records (73 records)
  → 1323 references
