# Supplementary material for: Pre-Participation Evaluation of Recreational and Competitive Athletes – A Systematic Review of Guidelines and Consensus Statements
Source: Sports Med Open. 2025 Apr 5;11:33. doi: 10.1186/s40798-025-00837-6 (PMC11972279; doi:10.1186/s40798-025-00837-6)
Supplement: Supplementary file 4 — Supplementary Material 4 [file 40798_2025_837_MOESM4_ESM.pdf]

Cover page

Supplement IV: Recommendations

Article title: Pre-participation evaluation of recreational and competitive athletes – A systematic review of guidelines and consensus statements

Journal: Sports Medicine - Open

Authors: Alina Weise, Nadja Könsgen, Christine Joisten, Fabian Schlumberger, Anja Hirschmüller, Jessica Breuing, Käthe Gooßen

Corresponding author: Dr Alina Weise, Witten/Herdecke University, Institute for Research in Operative Medicine (IFOM), Cologne, Germany. ORCID: https://orcid.org/0000-0003-4563-5782, alina.weise@uni-wh.de

Supplement IV –

Overview of extracted recommendations

Abbreviations

AAFP	American Academy of Family Physicians
AAP	American Academy of Pediatrics
AAOS	American Academy of Orthopaedic Surgeons
AAPMR	American Academy of Physical Medicine and Rehabilitation
AASP	Association of Applied Sport Psychiatry
ACC	American College of Cardiology
ACC SECLC	American College of Cardiology Sports and Exercise Cardiology Leadership Council
ACEP	American College of Emergency Physicians
ACL	anterior cruciate ligament
ACOG	American College of Obstreticians and Gynecologists
ACS	American Cancer Society
ACLM	American College of Lifestyle Medicine
ACRM	American Congress of Rehabilitation Medicine
ACSM	American College of Sports Medicine
ACSEP	Australasian College of Sports and Exercise Physicians
AEPC	AEPC
AGREE	Appraisal of Guidelines for Research and Evaluation
AH	athlete's heart
AHA	American Heart Association
AIS	Australian Institute of Sport
AMSSM	American Medical Society for Sports Medicine
AOASM	American Osteopathic Academy of Sports Medicine
AOSSM	American Orthopaedic Society for Sports Medicine
APA	American Psychological Association (Division 47: Exercise and Sport Psychology)
APHRS	Asia Pacific Heart Rhythm Society
APTA	American Physical Therapy Association
ASCA	American School Counselor Association
ASD	Academy for Sports Dentistry
ASE	American Society of Echocardiography
ASSMP	Austrian Society of Sports Medicine and Prevention
BASEM	British Association for Sports and Exercise Medicine
BSE CRY	British Society of Echocardiography and Cardiac Risk in the Young
BMI	body mass index
BP	blood pressure
CACHN	Community and Athletic Cardiovascular Health Network
CARF	Commission on Accreditation of Rehabilitation Facilities
CASEM	Canadian Academy of Sport and Exercise Medicine
CATA	Canadian Athletic Therapists Association
CATS	College Athletic Trainers' Society
CCS	Canadian Cardiovascular Society
CDC	Centers for Disease Control
CHRS	Canadian Heart Rhythm Society
CIHR	Canadian Institute of Health Research
CMR	cardiovascular magnetic resonance
COC	Canadian Olympic Committee
COPSI	Canadian Olympic and Paralympic Sport Institute Network
CPC	Canadian Paralympic Committee
CSCCA	Collegiate Strength and Conditioning Coaches Association
CSEP	Canadian Society for Exercise Physiology
CSI	Canadian Sport Institute
CVD	cardiovascular disease
DVGS	Deutscher Verband für Gesundheitssport und Sporttherapie
EA4SD	European Association for Sports Dentistry
EACPR	European Association for Cardiovascular Prevention and Rehabilitation
EACVI	European Association of Cardiovascular Imaging
ECG	electrocardiography
ECSEP	European College of Sports and Exercise Physicians
EFSMA	European Federation of Sports Medicine Associations
EHRA	European Heart Rhythm Association
EIB	Exercise-induced bronchoconstriction
EAPC	European Association of Preventive Cardiology
ESC	European Society of Cardiology
ESSA	Exercise and Sports Science Australia
FABER test	Flexion, Abduction and External Rotation test
FADIR test	Flexion, Adduction and Internal Rotation test
FATC	Female Athlete Triad Coalition
FMATC	Female and Male Athlete Triad Coalition
FIFA	Fédération Internationale de Football Association
HCM	hypertrophic cardiomyopathy
HR	heart rate
HRS	Heart Rhythm Society
HSF	Heart and Stroke Foundation
IAEHC	Italian Association of Extra-hospital Cardiologists
IAIHC	Italian Association of In-hospital Cardiologists
ICISF	International Critical Incident Stress Foundation
IOC	International Olympic Committee
ISC	Italian Society of Cardiology
ISSP	International Society of Sports Psychiatry
LASECS	LASECS
LoE	Level of Evidence
MCS	MacMillan Cancer Support
MET	metabolic equivalent(s)

MTSS	medial tibial stress syndrome
NAIAAA	National Interscholastic Athletic Administrators Association
NASMPA	Norwegian Association of Sports Medicine and Physical Activity
NATA	National Athletic Trainers' Association
NCAA	National Collegiate Athletics Association
NCCN	National Comprehensive Cancer Network
NCI	National Cancer Institute
NCSF	National Council on Strength and Fitness
NFHS	National Federation of State High School Associations
NSCA	National Strength and Conditioning Association
PAR-Q+	Physical Activity Readiness Questionnaire Plus
PPE	pre-participation physical examination
RDSPT	Royal Dutch Society for Physical Therapy
SASMA	South African Sports Medicine Association
SBC-DERC	Brazilian Society of Cardiology – Department of Exercise and Rehabilitation
SBM	Society for Behavioral Medicine
SCE	sudden cardiac death
SCCT	Society of Cardiovascular Computed Tomography
SCG	Sports Cardiology Group
SCMR	Society for Cardiovascular Magnetic Resonance
SCT	sickle cell trait
SDA	Sports Doctors Australia
SGS	Schweizerische Gesellschaft für Sportmedizin
SET	shin edema test
SORT	Strength of Recommendation Taxonomy
SPT	shin palpation test
SSC	Spanish Society of Cardiology
SSESM	Swedish Society of Exercise and Sports Medicine
TTE	trans-thoracic echocardiography

Tips for using this document

The strength of recommendations is distinguished according to this scheme, which extends the „Strength of Recommendation Taxonomy" (SORT).

SORT	Basis for strength of recommendation
A	Consistent, good-quality patient-oriented evidence
B	Inconsistent or limited-quality patient-oriented evidence
C	Consensus, disease-oriented evidence, usual practice, expert opinion, or case series for studies of diagnosis, treat-ment, prevention, or screening
–	Statement in the text

In addition, some authors assigned a class of recommendation, that reflects the consistence of the basis for decision making:

Class	Basis for class of recommendation
I	Evidence and/or general agreement that a given procedure or treatment is useful and effective
II	Conflicting evidence and/or divergence of opinion about the usefulness/efficacy of a procedure or treatment
III	Evidence and/or general agreement that the procedure/treatment is not useful/effective, and in some cases may be harmful

Definition of populations

Abbreviation	Definition
RA	Recreational atheltes (marked violet)
OS	Participants in organized sports (club sport)
CA	Competitive athletes (regular competitions)
EA	Elite athletes (national cadres, professional sport)

Administration

ID, Ref., Population[a]	Recommendation	LoE[b], Ref.	SORT[c]
	Privacy, data protection		
NATA 2014 [1], OS	Privacy must be respected at all times when the findings of the PPE are communicated. Written authorization must be provided by the athlete, or the legal guardian if the athlete is a minor, before any private health information is released.		**C**
AAP 2019 [2], OS-CA	Defined sections of the PPE are personal (or protected) health information and require confidential, secure handling and permission of the athlete, or parent, or guardian, to be shared beyond the athlete's health care personnel.		C
	Standardization, documentation		
AHA ACC 2015 [3-5], CA	It is recommended that standardization of the questionnaire forms used as guides for examiners of high school and college athletes in the United States be pursued.		**I-C**
NATA 2014 [1], OS	A standardized PPE is most desirable, and as research dictates specific recommendations for what is to be evaluated, a more standardized process should emerge. However, considerable variability still exists. The American Academy of Pediatrics has developed a thorough document that should serve as the minimum template for a standardized PPE instrument.		**C**
AAP 2019 [2], OS-CA	The standardized PPE History Form is the preferred format for documentation in either paper format or electronic format.		C
EFSMA 2015 [6], CA	Recommendations for PPE in Europe: Standardised history and clinical examination with „e-documentation"		C
EFSMA 2021 [7], EA	Standardised history and clinical examination with digital or 'e-documentation' are recommended, storing or paper documentation should be implicit avoided.		–
AMSSM 2017 [8], CA	A standardised questionnaire should be considered during the PPE and during well childcare visits that serve as the PPE to guide a comprehensive cardiac symptom and familiy history evaluation.		–
	Timing		
NATA 2014 [1], OS	The PPE may be conducted 4 to 6 weeks before preseason training begins to allow time for proper follow-up of any findings requiring additional evaluation. However, it is also practical and acceptable to conduct the PPE on the day preseason training begins or the day before because athletes usually report 1 to 2 days earlier. Because of this short timeline, clearance for some athletes who require additional evaluation may be delayed.		**C**
NATA 2014 [1], OS	A complete PPE should be performed at each new level of participation. When warranted during interim years, a review of the medical history and subsequent evaluation should be conducted.		**C**
AAP 2019 [2], OS-CA	The pre-participation physical evaluation (PPE) should be performed as part of the periodic health supervision examination every 2 to 3 years, with updated interval histories at intervening health supervision checks yearly.		C
AAP 2019 [2], OS-CA	Athletes and their parents or guardians are responsible for timely scheduling of PPEs, as problems that require additional testing may delay the completion of the examination in time for the start of season practices.		C
AEPC 2017 [9], CA	The screening should be performed before the start of competitive sports and should be repeated every second year to detect progressive diseases.		–
AMSSM 2017 [8], CA	Ideally, pre-participation cardiovascular screening should take place with adequate time prior to the start of a sports season to perform secondary testing of screening abnormalities.		–
	Setting		
NATA 2014 [1], OS	Both individual and multiple-station PPE screening methods can be effective and beneficial provided the appropriate personnel are available and a systematic approach is used to compile and record findings.		**C**
AAP 2019 [2], OS-CA	The pre-participation physical evaluation should be integrated into the periodic health evaluations of an athlete along with preventative care strategies by a provider from the medical home health care team who has current and past medical records.		**C**
AAP 2019 [2], OS-CA	The PPE is best performed in the setting of the primary medical home, by a provider who knows the athlete well or who has a comprehensive medical and injury history of the athlete.		C
AAP 2019 [2], OS-CA	The writing group does not recommend that these examinations be performed in a station-based or group setting or by providers who do not have access to the athlete's comprehensive medical and injury history. It is clear from the literature that these examinations are not equivalent to the examination performed by the primary medical home.		C
AAP 2019 [2], OS-CA	If a station-based or group examination is used, it is important that review of the history and physical examination be performed by the provider assessing medical eligibility and participation status.		C

ID, Ref., Population[a]	Recommendation	LoE[b], Ref.	SORT[c]
AAP 2019 [2], OS-CA	Adolescent athletes should be seen apart from their parents or guardians for at least part of the examination so that the provider can inquire about risk-taking behaviors.		C
	Infrastructure for cardiovascular screening		
ASE 2020 [10], CA	Pre-participation cardiovascular screening programs should ensure timely access to clinical centers with sports cardiology and clinical imaging expertise to facilitate the comprehensive multimodality imaging required to evaluate findings detected during pre-participation cardiovascular screening.		C
AMSSM 2017 [8], CA	Any ECG screening programme if implemented, however, should have a strong infrastructure, high quality control, and consider informed consent that outlines the potential benefits and risks with the athlete (and/or parent/guardian).		–
	Termination and further evaluations		
AAP 2019 [2], OS-CA	The final responsibility for a PPE lies with the provider who signs the Medical Eligibility Form and assumes the medical liability.		C
AAP 2019 [2], OS-CA	Athletes with problems discovered during the PPE that are beyond the scope and expertise of the examining provider should be referred to an appropriate specialist for consultation regarding medical eligibility.		C

[a] RA: recreational athletes, OS=participants in organized sports, A=competitive athletes, EA=elite athletes; [b] own assessment, only filled if literature could be clearly assigned;[c] bold if assigned by authors, otherwise own assessment.

Indication

ID, Ref., Population[a]	Recommendation	LoE[b], Ref.	SORT[c]
ACSM 2021 [11], RA	Preparticipation health screening before initiating a moderate-to-vigorous exercise program is a two-stage process:<br>- The need for medical clearance before initiating or progressing exercise programming is determined using the ACSM screening algorithm and the help of a qualified exercise or health care professional. In the absence of professional assistance, interested individuals may use the Physical Activity Readiness Questionnaire Plus (PAR-Q+).<br>- If indicated during screening, medical clearance from a physician or other qualified health care provider should be recommended. The manner of clearance, however, should be determined by the clinical judgment and discretion of said health care provider.		–
CASEM 2020 [12], RA	Participation in light to moderate exercise confers very little risk and can be 'self-administered', akin to an over-the-counter medication.		–
	pregnancy		
ACOG 2020 **[13]**, RA	A thorough clinical evaluation should be conducted before recommending an exercise program to ensure that a [pregnant] patient does not have a medical reason to avoid exercise.		C
ACOG 2020 **[13]**, RA	Obstetrician-gynecologists and other obstetric care providers should evaluate women with medical or obstetric complications carefully before making recommendations on physical activity participation during pregnancy.		C
ACSM 2021 [11], RA	The Canadian Society for Exercise Physiologists Physical Activity Readiness Medical Examination for Pregnancy (PARmed-X for Pregnancy) or the electronic Physical Activity Readiness Medical Examination (ePARmed-X+) can be used for the health screening of pregnant women before their participation in exercise programs.		–
IOC 2017 [14], RA-EA	For high level exercisers and elite athletes, a thorough clinical evaluation should be conducted to ascertain that there are no medical or obstetrical reasons to either avoid exercise completely, or to modify exercise routines (box 1, 2).		–
	Cancer survivors		
ACSM 2021 [11], RA	A preexercise medical assessment is suggested (Table 10.1).		–
ACSM 2021 [11], RA	Specific cancer survivor populations for whom medical evaluation and/or exercise testing should be considered include those with metastatic disease, those with persistent and significant cancer treatment-related side effects, or those with significant comorbidities.	3 [15]	–
ACSM 2021 [11], RA	Exercise testing is not required for preparticipation assessment for most cancer survivors.	3 [15]	–
ACSM 2021 [11], RA	The ACSM preparticipation screening algorithm can be used to determine whether exercise testing is needed for cancer survivors prior to participation in moderate-to-vigorous intensity exercise.		–

ID, Ref., Population[a]	Recommendation	LoE[b], Ref.	SORT[c]
ACSM 2019 [16], RA	Pre-exercise medical evaluation (per NCCN guidelines for specific symptoms and side effects) for patients with lung or abdominal surgery, ostomy, cardiopulmonary disease, ataxia, extreme fatigue, severe nutritional deficiencies, worsening/changing physical condition (i.e., lymphedema exacerbation), bone metastases.<br>Consider referral to trained personnel (rehabilitation specialists (i.e., physical the rapists, occupational therapists, physiatrists) and certified exercise physiologists).		–
ACSM 2019 [16], RA	Recommend pre-exercise medical evaluation (per NCCN guidelines for specific symptoms and side effects) for patients with peripheral neuropathy, arthritis/musculoskeletal issues, poor bone health (e.g., osteopenia or osteoporosis), lymphedema.<br>Consider referral to trained personnel (rehabilitation specialists (i.e., physical therapists, occupational therapists, physiatrists) and certified exercise physiologists).		–
ACSM 2019 [16], RA	No further pre-exercise medical evaluation (per NCCN guidelines for specific symptoms and side effects) for patients without comorbidities.		–

[a] RA: recreational athletes, OS=participants in organized sports, A=competitive athletes, EA=elite athletes; [b] own assessment, only filled if literature could be clearly assigned;[c] bold if assigned by authors, otherwise own assessment.

Scope, test selection

ID, Ref., Population[a]	Recommendation	LoE[b], Ref.	SORT[c]
ACSM 2021 [11], RA	Recommended laboratory tests, depending on individual risk factors, signs, and symptoms, could include fasting serum total cholesterol, fasting plasma glucose, 12-lead ECG, Holter monitoring, cardiac echocardiography, chest radiography, pulmonary function, and oximetry.		C
EFSMA 2021 [7], EA	Establishing the objective health status by physical examination includes the evaluation of dermatological conditions, lymph nodes, musculoskeletal system, respiratory system, cardiovascular system and resting ECG with 12 leads digital recording and software supported. Abdominal, neurological, ophthalmological and ENT examinations, blood and biochemical tests, urine samples should also be assessed.		–
EFSMA 2021 [7], EA	component of PPE is evaluating functional and exercise capacity according to the predominant type of effort of the sports discipline investigated. This includes neuromuscular evaluation (determination of muscle fibre strength and driving speed, EMG, EEG and Sport Concussion Assessment Tool in contact sports), psychological examination (testing aptitude, adaptation to training and personality traits). Mental health status is vital to discover any obstacles to achieving performance.		–
AAP 2019 [2], OS-CA	PPE working group concurs that no routine [laboratory, cardiac, and pulmonary] screening tests are required during the PPE for determining medical eligibility of asymptomatic athletes.		–
AAP 2019 [2], OS-CA	Findings from the PPE medical history or physical examination may indicate a need to arrange specific case finding [laboratory, cardiac, and pulmonary] diagnostic tests. Additional specific recommendations to test for targeted conditions are not part of the standardized screening examination.		–
EFSMA 2021 [7], EA	The PPE should entail the following diagnostic components:<br>► Health status.<br>► Anthropometry.<br>► Functional and exercise capacity.		–
	Athletes with disabilities		
AAP 2019 [2], OS-A	The preparticipation physical evaluation (PPE) for an athlete with a disability should be similar to an athlete with no disabilities along with addressing the unique needs of the specific disability. The health care professional should be aware of common problems associated with different disabilities and be able to identify and help manage conditions that may compromise athlete safety. Just as important, the health care professional should encourage physical activity and provide support as needed.		–

[a] RA: recreational athletes, OS=participants in organized sports, A=competitive athletes, EA=elite athletes; [b] own assessment, only filled if literature could be clearly assigned;[c] bold if assigned by authors, otherwise own assessment.

Medical and family history

ID, Ref., Population[a]	Recommendation	LoE[b], Ref.	SORT[c]
NATA 2014 [1], OS	A comprehensive medical and family history should be obtained from each participant. This is the cornerstone of the PPE and should take into account the areas of greatest concern for sport participation: specifically, the American Heart Association recommendations for preparticipation cardiovascular screening of competitive athletes (Table 2).		**B**
NATA 2014 [1], OS	The medical and family history provided by the athlete and the parents or guardians should always be reviewed carefully. Both parties should be questioned and specific answers confirmed because the source that provides the most accurate history is unclear.	3 [17]<br>4 [18]	**C**
NATA 2014 [1], OS	All medications and supplements currently used by the athlete should be reviewed by the examiner during the PPE.		**C**
AAP 2019 [2], OS-CA	For athletes younger than 18 years, both the athlete and the parent or guardian are asked to respond to medical history questions.	4 [18]	–
ACSM 2021 [11], RA	Appropriate components of the medical history may include the following:<br>• Medical diagnoses and history of medical procedures: cardiovascular disease risk factors including hypertension, obesity, dyslipidemia, and diabetes; cardiovascular disease including heart failure, valvular dysfunction (e.g., aortic stenosis/mitral valve disease), myocardial infarction, and other acute coronary syndromes; percutaneous coronary interventions including angioplasty and coronary stent(s), coronary artery bypass surgery, and other cardiac surgeries such as valvular surgeries; cardiac transplantation; pacemaker and/or implantable cardioverter defibrillator; ablation procedures for dysrhythmias; peripheral vascular disease; pulmonary disease including asthma, emphysema, and bronchitis; cerebrovascular disease including stroke and transient ischemic attacks; anemia and other blood dyscrasias (e.g., lupus erythematosus); phlebitis, deep vein thrombosis, or emboli; cancer; pregnancy; osteoporosis; musculoskeletal disorders; emotional disorders; and eating disorders<br>• Previous physical examination findings: murmurs, clicks, gallop rhythms, other abnormal heart sounds, and other unusual cardiac and vascular findings; abnormal pulmonary findings (e.g., wheezes, rales, crackles), high blood pressure, and edema<br>• Laboratory findings (i.e., plasma glucose, HbA1C, hs-CRP, serum lipids and lipoproteins)<br>• History of symptoms: discomfort (e.g., pressure, tingling sensation, pain, heaviness, burning, tightness, squeezing, numbness) in the chest, jaw, neck, back, or arms; light-headedness, dizziness, or fainting; temporary loss of visual acuity or speech; transient unilateral numbness or weakness; shortness of breath; rapid heartbeat or palpitations, especially if associated with physical activity, eating a large meal, emotional upset, or exposure to cold (or any combination of these activities)<br>• Recent illness, hospitalization, new medical diagnoses, or surgical procedures<br>• Orthopedic problems including arthritis, joint swelling, and any condition that would make ambulation or use of certain test modalities difficult<br>• Medication use (including dietary/nutritional supplements) and drug allergies<br>• Other habits including caffeine, alcohol, tobacco, or recreational (illicit) drug use<br>• Exercise history: information on readiness for change and habitual level of activity: frequency, duration or time, type, and intensity or FITT of exercise<br>• Work history with emphasis on current or expected physical demands, noting upper and lower extremity requirements<br>• Family history of cardiac, pulmonary, or metabolic disease, stroke, or sudden death		–
EFSMA 2021 [7], EA	Health assessment is based on family medical history, personal medical history, training history, training information and medical complaints: symptoms and signs.		–
	History taking after COVID-19		
AMSSM 2022 [19, 20], CA	Use the PPE as an opportunity to identify and record the following information:<br>- Prior SARS-CoV-2 infection? If so, when did the infection occur, what symptoms were experienced and for how long. Were diagnostic tests performed (eg, echocardiogram and chest x-ray)? Was there a need for any physician-directed treatment or hospitalization? Presence of postinfection sequelae (eg, myocarditis)? Did the athlete achieve full recovery and return to play? Is the athlete experiencing persistent symptoms? If so, what symptoms? Is the athlete experiencing new symptoms with exercise (especially chest pain or excessive shortness of breath)?<br>- COVID vaccination status? Which vaccine was received? Dates of administration? Reaction(s) to vaccination?		C
	Questions on sexual violence and sexual abuse		

ID, Ref., Population[a]	Recommendation	LoE[b], Ref.	SORT[c]
AAP 2019 [2], OS-CA	During a PPE, sexual violence and sexual abuse will not be discovered if the question is not asked. If suspected child or adult sexual abuse is documented or suspected during the PPE, immediate notification of the Department of Children's Services or a law enforcement agency of the suspected abuse is mandatory.<br>There is not a legal requirement for the reporting chain to go through any athletic department or school supervisor or administrator.		–
	Athletes with disabilities		
AAP 2019 [2], OS-CA	The history should include a detailed summary of previous injuries and illnesses, risk factors for injuries and illnesses, and current medications.		–
AAP 2019 [2], OS-CA	In addition to the questions asked of an athlete who does not have a cognitive or physical disability (see Chapter 5 and the History Form on pages 217 and 218), additional questions should specifically address the particular impairment. The questions that follow emphasize areas of greatest concern for sports participation.<br>1. Does the athlete have a history of seizures? Are the seizures controlled?<br>2. Does the athlete have a history of hearing loss or impairment?<br>3. Does the athlete have a history of vision loss or impairment?<br>4. Does the athlete have a history of cardiopulmonary disease?<br>5. Does the athlete have a history of renal disease or unilateral kidney?<br>6. Does the athlete have a history of symptomatic atlantoaxial instability (AAI)?<br>7. Has the athlete had heat stroke or heat exhaustion?<br>8. Has the athlete had any fractures or dislocations?<br>9. What prosthetic devices or other assistive equipment does the athlete use during sports participation?<br>10. Does the athlete use an indwelling urinary catheter or require intermittent catheterization of the bladder?<br>11. Does the athlete have a history of pressure sores or ulcers?<br>12. At what levels of competition has the athlete previously participated?<br>13. What is the athlete's level of independence for mobility and self-care?<br>14. What medications is the athlete taking?<br>15. Does the athlete have any dietary restrictions?<br>16. Does the athlete have a history of autonomic dysreflexia?		–
AAP 2019 [2], OS-CA	The PPE physical examination for an athlete who is disabled should include all parts of the examination for an athlete with no disability (see Chapter 5 and the Physical Examination Form on page 221). Particular attention should be given to the ocular, cardiovascular, musculoskeletal, neurological, and dermatologic systems.<br>In addition to examining the athlete, a qualified health care professional or prosthetist should thoroughly inspect all prosthetic devices, orthoses, and assistive or adaptive devices to ensure all fit properly during exercise and are within the rules of competition.		–
	Cancer survivors		
ACSM 2019 [16], RA	Be aware of a survivor's health history, comorbid chronic diseases, and health conditions, and any general exercise contraindications before commencing health-related fitness assessments or designing the exercise prescription.		C
ACSM 2019 [16], RA	Be familiar with the most common toxicities associated with cancer treatments including increased risk for fractures and cardiovascular events, along with neuropathies or musculoskeletal morbidities related to specific types of treatment.		C

[a] RA: recreational athletes, OS=participants in organized sports, A=competitive athletes, EA=elite athletes; [b] own assessment, only filled if literature could be clearly assigned;[c] bold if assigned by authors, otherwise own assessment.

Physical examination

ID, Ref., Population[a]	Recommendation	LoE[b], Ref.	SORT[c]
NATA 2014 [1], OS	For the PPE, a limited general physical examination is recommended. The screening physical should include vital signs (eg, height, weight, and blood pressure); visual acuity testing; cardiovascular, neurologic, and general medical (eg, pulmonary, abdominal, skin, genitalia [for males]) examination; and musculoskeletal examination. Further examination should be based on issues uncovered during the history.	3 [21]	**C**

ID, Ref., Population[a]	Recommendation	LoE[b], Ref.	SORT[c]
EFSMA 2021 [7], EA	The physical examination of PPE is very important and it should be performed with a particular focus on cardiovascular, pulmonary, musculoskeletal, neurological, ophthalmological and otolaryngology screening.		–
ACSM 2021 [11], RA	Appropriate components of the physical examination may include the following:<br>• Body weight; in many instances, determination of body mass index, waist girth, and/or body composition (body fat percentage) is desirable.<br>• Apical pulse rate and rhythm<br>• Resting blood pressure: seated, supine, and standing<br>• Auscultation of the lungs with specific attention to uniformity of breath sounds in all areas (absence of rales, wheezes, and other breathing sounds)<br>• Palpation of the cardiac apical impulse and point of maximal impulse<br>• Auscultation of the heart with specific attention to murmurs, gallops, clicks, and rubs<br>• Palpation and auscultation of carotid, abdominal, and femoral arteries<br>• Evaluation of the abdomen for bowel sounds, masses, visceromegaly, and tenderness<br>• Palpation and inspection of lower extremities for edema and presence of arterial pulses<br>• Absence or presence of tendon xanthoma and skin xanthelasma<br>• Follow-up examination related to orthopedic or other medical conditions that would limit exercise testing<br>• Tests of neurologic function including reflexes and cognition (as indicated)Inspection of the skin, especially of the lower extremities in known patients with diabetes mellitus		–
AAP 2019 [2], OS-CA	The structured physical examination begins by measuring vital signs and visual acuity.	4 [22]	–
	Ophthalmology		
AAP 2019 [2], OS-CA	All athletes should have their visual acuity checked at the PPE.		–
	Urogenital		
AAP 2019 [2], OS-CA	Palpate the abdomen for masses or enlarged organs (enlarged spleen or liver, enlarged kidney, or gravid uterus). The abdominal examination should be performed with the athlete supine.		–
AAP 2019 [2], OS-CA	In addition to the general abdominal assessment, if there is a high index of suspicion for an eating disorder, the gastrointestinal tract can be more closely evaluated.		–
AAP 2019 [2], OS-CA	A GU examination of female athletes is not part of the PPE.		–
	Dermatology		
AAFP 2016 [23], CA	Illness Reduction and Modification Evaluation (skin infections): Athletes should have an evaluation prior to participation including:<br>o History of previous communicable skin lesions and treatment<br>o Comprehensive assessment for skin breaks and wounds<br>o Identification of type and location of current communicable skin lesions and treatment		–
AAP 2019 [2], OS-CA	A thorough skin survey should be performed with particular attention to exposed areas and any areas that might potentially come into contact with another competitor or equipment (eg, wrestling mats, batting helmets). The principle objective in the dermatologic examination is to identify skin infections: bacterial, viral, fungal, and infestations.		–
AAP 2019 [2], OS-CA	The examiner should also look for signs of trauma, acne, sun damage, and dermatitis (contact dermatitis, eczema, psoriasis, or urticarial dermatitis), as well as marks of illicit drug use or cutting.	4 [24]<br>2 [25]	–
AAP 2019 [2], OS-CA	Prosthetic devices can cause skin trauma; the prosthesis contact site should be inspected for abrasions, blisters, rashes, or pressure ulcers. The prosthesis should be evaluated for proper fit and reconditioned to decrease the risk of future problems.		–
	Denttistry		
EA4SD 2020 [26], CA	The integration of dentistry into sports medicine should focus on the most common diseases found in athletes, including dental caries, dental erosion, periodontal disease, malocclusion, temporomandibular disorders (TMD), orofacial injuries, and their prevention. This integration should lead to a multidisciplinary follow up of athletes including oral screening and relevant oral health treatments.		–
	Pregnancy		

ID, Ref., Population[a]	Recommendation	LoE[b], Ref.	SORT[c]
AAFP 2017 [27], CA	An evaluation includes facilitating or performing a medical examination, nutritional assessment, and ongoing assessment of absolute and relative contraindications to exercise throughout pregnancy and the postpartum period.		–
	Athletes with disabilities		
AAP 2019 [2], OS-CA	The upper extremities should be examined for abrasions and blisters caused by friction, shear, or irritation from repeated contact with the wheelchair push rim.<br>The skin over the sacrum and ischial tuberosities should be inspected for pressure ulcers.<br>The seat cushion should also be evaluated and modified to decrease skin pressures, improve the skin healing, and reduce the risk of further skin trauma.		–
AAP 2019 [2], OS-CA	[Urogenital] examination should involve the same evaluation as for athletes who are nondisabled.<br>It is also important to examine any external devices used for bladder drainage.		–

[a] RA: recreational athletes, OS=participants in organized sports, A=competitive athletes, EA=elite athletes; [b] own assessment, only filled if literature could be clearly assigned;[c] bold if assigned by authors, otherwise own assessment.

Anthropometry

ID, Ref., Population[a]	Recommendation	LoE[b], Ref.	SORT[c]
EFSMA 2021 [7], EA	Part of the PPE is the anthropometric examination that includes the somatoscopy, the classic method of assessing posture and body development by visual observation of the athlete in the anthropometric frame work and biometrics (weight, height, sitting height, body composition, girth measurements, diameters, mobility and strength measurements) to assess body shape, posture, nutrition, body symmetry, harmony in development, compared with the somatic biotype of the sport.		–
AAP 2019 [2], OS-CA	Athletes who have conditions such as underweight, overweight, or obesity have long-term health issues that may affect medical eligibility and risk for injury or illness during sport participation. During the PPE, values for height, weight, and body mass index (BMI) should be plotted on age-appropriate growth charts and compared, if possible, with past measurements.		–
AAP 2019 [2], OS-CA	Review of growth curve measurements can identify atypical weight loss or gain that may suggest disordered eating or insufficient energy availability, unhealthy weight-cutting behaviors in sports with weight classes, or performance-enhancing supplement use that may not be otherwise identified.<br>Decreased height velocity or delayed puberty may also indicate other medical problems such as endocrine or nutritional problems and deserves further evaluation.<br>Body mass index curves should also be similarly reviewed but may be falsely skewed toward overweight status by muscle development in some athletes.		–

[a] RA: recreational athletes, OS=participants in organized sports, A=competitive athletes, EA=elite athletes; [b] own assessment, only filled if literature could be clearly assigned;[c] bold if assigned by authors, otherwise own assessment.

Nutrition

ID, Ref., Population[a]	Recommendation	LoE[b], Ref.	SORT[c]
NATA 2014 [1], OS	Athletic trainers and those who participate in athletic health care should be familiar with the current NATA position statement that outlines the prevention, detection, and management of disordered eating in athletes.		**B**
AMSSM 2020 [28], CA	Annual preparticipation screening for eating disorders in athletes should be routine.		**C**
IOC 2018 [29, 30], CA	HealthCare professionals can decrease the health implications of RED-S through (…) Implementation of the RED-S Risk Assessment Model in the periodic health examination and the RED-S return to play Model.		C
EFSMA 2021 [7], EA	it would be important for the healthcare professional to perform a comprehensive nutritional assessment and recommend using supplements only when necessary.		–
IOC 2018 [29, 30], CA	Screening for RED-S should be undertaken as part of an annual Periodic Health Examination (PHE) and when an athlete presents with DE/ED, weight loss, lack of normal growth and development, menstrual dysfunction, recurrent injuries and illnesses, decreased performance or mood changes.		–

ID, Ref., Population[a]	Recommendation	LoE[b], Ref.	SORT[c]
IOC 2013 [31], CA	Optimum screening times [for disordered eating/eating disorders] occur at the PPE and annual health check-ups.		–
IOC 2013 [31], CA	we suggest that when healthcare providers suspect DE or EDs, an Anthropometric, Biochemical, Clinical, Dietary and Environmental (ABCDE) Assessment be used to evaluate athletes (table 4).		–
AAFP 2017 [27], CA	It is desirable the team physician (…) identify and address risk factors [for DE/ED] during the PPE and subsequent clinical encounters, including specific survey tools (e.g., SCOFF, EDI, and LEAF-Q).		–

[a] RA: recreational athletes, OS=participants in organized sports, A=competitive athletes, EA=elite athletes; [b] own assessment, only filled if literature could be clearly assigned;[c] bold if assigned by authors, otherwise own assessment.

(Fe)male athlete triad

ID, Ref., Population[a]	Recommendation	LoE[b], Ref.	SORT[c]
NATA 2014 [1], OS	For females who have abnormal menstrual cycles or a personal history of anemia or who are taking iron or other medications, a more detailed laboratory follow-up is warranted.		**C**
FMATC 2021 [32, 33], CA	It is recommended that clinicians screen the at-risk male athlete with targeted screening questions and risk assessment tools. Further research is needed to validate a best practice screening questionnaire for the Male Athlete Triad.		**C**
FATC 2014 **[34]**, CA	The Consensus Panel recommended that female athletes undergo annual screening with the Triad specific self-report questionnaire displayed in box 1, followed by a more in-depth evaluation if the athlete has or is at risk for any Triad component.		–
FATC 2014 **[34]**, CA	Screening for the Triad should be undertaken as part of the Pre-Participation Physical Evaluation (PPE).		–
AAFP 2017 [27], CA	All female athletes should be screened for LEA, DE/ED, menstrual dysfunction, and low bone mass/osteoporosis. Female athletes with amenorrhea, prolonged oligomenorrhea, or a history of BSI are at high risk. [...] Coordinate a screening evaluation process, during the PPE and routine visits, to identify at-risk female athletes		–
FATC 2014 **[34]**, CA	While such screening is most typically completed at the collegiate level, the Panel recommended screening for younger athletes (high school age) as well.	2 [35]<br>3 [36]	–
FATC 2014 **[34]**, CA	A medication history should be obtained, including medications which may affect menstruation and/or BMD, such as oral contraceptive pills or other contraceptive agents, such as depot medroxyprogesterone acetate.	3 [37]	–
FATC 2014 [34], CA	The Panel stated that the risk factors that should be assessed for the Triad include: (1) history of menstrual irregularities and amenorrhoea; (2) history of stress fractures; (3) history of critical comments about eating or weight from parent, coach or teammate; (4) a history of depression; (5) a history of dieting; (6) personality factors (such as perfectionism and obsessiveness); (7) pressure to lose weight and/or frequent weight cycling; (8) early start of sport-specific training; (9) overtraining; (10) recurrent and non-healing injuries and (11) inappropriate coaching behaviour.	2 [38]<br>2 [39]<br>3 [40]<br>3 [41]<br>3 [42]<br>3 [43]<br>4 [44]	–
AAP 2019 [2], OS-CA	A history of acute fractures or previous bone stress injuries should lead to further inquiry regarding menstrual history, nutritional status, bone health, eating patterns, and body image concerns, in addition to assessing for training errors, as a cause. Evaluation of nutrition status should include total calorie, calcium, and vitamin D intakes.		–
FATC 2014 **[34]**, CA	Existence of any one Triad component should prompt more thorough investigation for the others.		–
AAP 2019 [2], OS-CA	Underweight athletes should undergo a thorough medical evaluation, must be asked about emotions related to food and body image, and must be counseled about proper weight and nutrition. Similar concern should be raised for younger athletes who drop one percentage line on their growth chart and even more so for those who drop 2 lines.		–
FATC 2014 **[34]**, CA	Physical examination signs such as low body mass index (BMI), weight loss, orthostatic hypotension, lanugo, hypercarotenaemia, or other signs of an ED, such as parotid gland swelling and callus on the proximal interphalangeal joints (also known as Russell's sign), should also prompt further evaluation.		–
AAFP 2017 [27], CA	It is desirable the team physician (…) identify multifactorial risk factors [for menstrual dysfunction] during the PPE and subsequent clinical encounter.		–
FATC 2014 [34], CA	Obtaining an accurate menstrual history is important, starting from age of menarche to the current and the past menstrual patterns, noting months of consecutive missed menses and the number of menses per year since menarche.		–

ID, Ref., Population[a]	Recommendation	LoE[b], Ref.	SORT[c]
AAP 2019 [2], OS-CA	Amenorrhea, both primary and secondary, should be evaluated with additional history.		–
FATC 2014 [34], CA	A history of physician diagnosed bone stress injuries and other fracture history should be noted, as well as a family history of ED, osteoporosis and/or fractures.		–
AAP 2019 [2], OS-CA	Any athlete (female or male) with a history of stress fractures should be queried about dietary restriction behaviors that result in low energy availability, and inadequate calories, macronutrient imbalances, calcium intake, and vitamin D intake.		–
AAFP 2017 [27], CA	Multiple risk factors exist for BSI [bone stress injuries] and should be identified and addressed during the PPE.		–

[a] RA: recreational athletes, OS=participants in organized sports, A=competitive athletes, EA=elite athletes; [b] own assessment, only filled if literature could be clearly assigned;[c] bold if assigned by authors, otherwise own assessment.

Heat and hydration

ID, Ref., Population[a]	Recommendation	LoE[b], Ref.	SORT[c]
NATA 2014 [1], OS	Current consensus guidelines for heat acclimatization in secondary school athletes should be reviewed. Questions related to previous problems associated with heat acclimatization should be included in the medical history form.		**B**
NATA 2012 [45], OS	In conjunction with preseason screening, athletes should be questioned about risk factors for heat illness or a history of heat illness.		**C**
AAP 2019 [2], OS-CA	The PPE should include specific questions regarding possible risk factors, including prior heat illness, the associated environment, acclimatization status, equipment and uniforms, fluid intake, weight changes during activity, and medication and supplement use.		–
AAFP 2016 [23], CA	Injury Reduction and Modification Evaluation (heat illness): Athletes should have a thorough preseason evaluation, including:<br>o History of risk factors<br>o Evaluation of fluid intake<br>o Evaluation of present and anticipated volume/ intensity of training and participation<br>o Evaluation of athlete's state of acclimatization<br>o Screening for sickle cell trait		–

[a] RA: recreational athletes, OS=participants in organized sports, A=competitive athletes, EA=elite athletes; [b] own assessment, only filled if literature could be clearly assigned;[c] bold if assigned by authors, otherwise own assessment.

Cardiology

ID, Ref., Population[a]	Recommendation	LoE[b], Ref.	SORT[c]
	Test selection		
AHA ACC 2015 [3-5], RA-CA	Screening with 12-lead ECGs (or echocardiograms) in association with comprehensive history-taking and physical examination to identify or raise suspicion of genetic/congenital and other cardiovascular abnormalities may be considered in relatively small cohorts of young healthy people 12 to 25 years of age, not necessarily limited to competitive athletes (eg, in high schools, colleges/universities or local communities). Close physician involvement and sufficient quality control is mandatory. If undertaken, such initiatives should recognize the known and anticipated limitations of the 12-lead ECG as a population screening test, including the expected frequency of false-positive and false-negative test results, as well as the cost required to support these initiatives over time.		**II-C**
AHA ACC 2015 [3-5], RA-CA	Mandatory and universal mass screening with 12-lead ECGs in large general populations of young healthy people 12 to 25 years of age (including on a national basis in the United States) to identify genetic/congenital and other cardiovascular abnormalities is not recommended for athletes and non athletes alike.		**III-C**
AHA ACC 2015 [3-5], RA-CA	Consideration for large-scale, general population, and universal cardiovascular screening in the age group 12 to 25 years with history taking and physical examination alone is not recommended (including on a national basis in the United States).		**III-C**
EHRA EACPR 2017 [46], RA-CA	The protocol of PPE including clinical history, physical examination, and 12-lead ECG demonstrates to have superior diagnostic capability than just clinical history and physical examination.		–

ID, Ref., Population[a]	Recommendation	LoE[b], Ref.	SORT[c]
	Further evalations		
EHRA EACPR 2017 [46], RA-CA	Available data suggests that routine echocardiography or other imaging modalities do not add substantial diagnostic power to the PPE as a mass screening technique and do not appear to be cost/effective. Therefore, at the moment the ECG-based PPE represents the most effective protocol to evaluate athletes (i.e. best clinical practice), although several limitations should be acknowledged		–
	Age >35 years		
ESC 2021 [47], RA	In selected individuals without known CAD who have very high CVD risk (e.g. SCORE>10%, strong family history, or familial hypercholesterolaemia) [age > 35] and want to engage in high- or very high-intensity exercise, risk assessment with a functional imaging test, coronary CCTA, or carotid or femoral artery ultrasound imaging may be considered.		**IIb-B**
ESC 2021 [47], RA	Among individuals [age > 35] with low to moderate CVD risk, the participation in all recreational sports should be considered without further CV evaluation.		**IIa-C**
ESC 2021 [47], RA	Clinical evaluation, including maximal exercise testing, should be considered for prognostic purposes in sedentary people and individuals with high or very high CV risk [age > 35] who intend to engage in intensive exercise programmes or competitive sports.		**IIa-C**
	Older adults / age >65 years		
ESC 2021 [47], RA	A full clinical assessment including a maximal exercise test should be considered in sedentary adults aged 65 years or older who wish to participate in high-intensity activity.		**IIa-C**
ESC 2022 [48], RA-CA	The cardiovascular risk of middle-aged and elderly individuals should be evaluated before engaging in strenuous sports through established scores such as the SCORE2 risk chart.	1 [49]<br>2 [50]<br>2 [51]	**IIa-C**
EFSMA 2015 [6], RA	Recommendations for PPE in Europe: In the elderly, ECG is mandatory in all leisure time athletes (female and male).		–
ACSM 2021 [11], RA	Most older adults do not require an exercise test prior to initiating a moderate intensity PA program (see Chapter 2). However, if exercise testing is recommended, it should be noted that the associated electrocardiogram (ECG) has higher sensitivity (i.e., ~84%) and lower specificity (i.e., ~70%) than in younger age groups (i.e., <50% sensitivity and >80% specificity), producing a higher proportion of false positive outcomes.		–
ACSM 2021 [11], RA	Special considerations when testing older adults include the following<br>• Initial workload should be light (i.e., <3 METs) and workload increments should be small (i.e., 0.5–1.0 MET) for those with low work capacities. The modified Naughton treadmill protocol is a good example of such a protocol (see Figure 4.1) (97).<br>• A cycle ergometer may be preferable to a treadmill for those with poor balance, poor neuromotor coordination, impaired vision, impaired gait patterns, weight-bearing limitations, and/or orthopedic problems. However, local muscle fatigue may be a factor for premature test termination when using a cycle ergometer (97).<br>• Adding a treadmill handrail support may be required because of reduced balance, decreased muscular strength, poor neuromotor coordination, and fear. However, handrail support for gait abnormalities will reduce the accuracy of estimating peak MET capacity based on the exercise duration or peak workload achieved (97).<br>• Treadmill workload may need to be adapted according to walking ability by increasing grade rather than speed (97).<br>• Many older adults exceed the age-predicted HRmax during a maximal exercise test. The frequently used (220 − age) HRmax equation tends to underpredict HRmax in older adults (100); therefore, it is best to use other HRmax equations (see Table 5.3).<br>• The influence of prescribed medications on the ECG and hemodynamic responses to exercise may differ from usual expectations (see Appendix A).	3 [52]<br>3 [53]	–
	Pregnancy		
ACSM 2021 [11], RA	Maximal exercise testing should not be performed on women during any stage of pregnancy (142,143). If a submaximal exercise test is warranted, the test should be performed with physician supervision after the woman has been medically evaluated for contraindications to exercise.	4 [54]	–
	Cancer survivors		
ESC 2021 [47], RA	Among individuals treated with cardiotoxic medications, echocardiography before participation in high-intensity exercise is recommended.		**I-A**
ACSM 2019 [16], RA	CVD has become a competing cause of morbidity and mortality for survivors of cancer with a favorable prognosis. Given the potential for underlying CVD, cancer survivors should be screened	3 [55]	C

ID, Ref., Population[a]	Recommendation	LoE[b], Ref.	SORT[c]
	for evident or underlying CVD using the ACSM pre-participation guidelines and if implicated have a cardiopulmonary exercise test prior to beginning an exercise program.		

[a] RA: recreational athletes, OS=participants in organized sports, A=competitive athletes, EA=elite athletes; [b] own assessment, only filled if literature could be clearly assigned;[c] bold if assigned by authors, otherwise own assessment.

ID, Ref., Population[a]	Recommendation	LoE[b], Ref.	SORT[c]
ESC 2022 [48], CA	Pre-participation cardiovascular evaluation of competitive athletes should be considered.	1 [49]<br>4 [56]	**IIa-C**
NATA 2013 [57], CA	Athletes should undergo cardiovascular screening before participation in competitive athletics.		C
ESC 2022 [48], CA	It should be considered that cardiovascular evaluation of young (<35 years) competitive athletes includes history, physical examination, and 12-lead ECG.	3 [58]<br>4 [59]<br>4 [60]<br>4 [61]	**IIa-C**
CCS CHRS 2019 [62], CA	We recommend an incremental (tiered) approach to CV screening of competitive athletes as part of a broad, organization/athlete-centred CSCAP. Such screening should occur in the context of a consistent, systematic approach to CV screening and care that provides assessment, appropriate investigations, interpretation, management, counselling, follow-up.		I-C
AMSSM 2017 [8], CA	Considerations for implementing a cardiovascular screening strategy in a target athlete population should include the risk of SCA/D, the available infrastructure and cardiology resources, and the physician assessment that screening for early detection of cardiac disorders has a favorable risk-benefit ratio that will improve athlete outcomes with limited harm.		–
BSE CRY 2018 [63], CA	Type of athletic activity should be known.		–
BSE CRY 2018 [63], CA	Amount of athletic activity should be known.		–
	Recommendations focussing on medical/family history		
NATA 2014 [1], OS	Specific questions regarding risk factors and symptoms of cardiovascular disease should be asked during the history portion of the PPE (Table 3). A positive response to any question should be confirmed and further evaluation conducted if necessary.		**C**
NATA 2012 [45], OS	The preparticipation physical examination should include the completion of a standardized history form and attention to episodes of exertional syncope or presyncope, chest pain, a personal or family history of sudden cardiac arrest or a family history of sudden death, and exercise intolerance.		**C**
CCS CHRS 2019 [62], CA	We recommend that a history/questionnaire should constitute the initial CV screening (tier 1), provided it is:<br>i. Standardized according to at least 1 of the American Heart Association, European Society of Cardiology, fourth-edition Preparticipation Physical Evaluation, or SportsCardiologyBC tools or equivalent;<br>ii. Accurately interpreted by an appropriately qualified professional experienced in the care of athletes; and<br>iii. Is followed with appropriate investigations as “clinically warranted.” “Clinically warranted” findings are those deemed by the interpreter as requiring further assessment, for example using: physical examination; investigations such as ECG testing, imaging, or stress testing; and/or consultation with a specialist.		I-C
AAP 2019 [2], OS-CA	A history of previously performed cardiac testing such as ECG, echocardiography, or exercise treadmill testing may indicate a previously suspected cardiac disorder, and a careful review of past medical records may establish the outcomes of the evaluation. This should preclude repeating the studies.		–
AAP 2019 [2], OS-CA	• A detailed personal and family history may help identify athletes at risk for SCD.<br>• Sudden cardiac death may be the first manifestation of underlying cardiac disease; warning symptoms that require cardiac workup include syncope and/or chest pain during exercise, palpitations during exercise, unexplained breathlessness during exercise, and unexplained seizures.<br>• A family history of sudden unexpected or unexplained death, sudden death before the age of 50 years (especially younger than age 35) caused by cardiac problems, sudden infant death, unexplained drowning, unexplained near drowning, car crashes caused by unexplained driver loss of consciousness, or unexplained seizures may indicate the presence of a genetic cardiovascular disorder placing the athlete at increased risk for SCD.		–

ID, Ref., Population[a]	Recommendation	LoE[b], Ref.	SORT[c]
	Recommendations focussing on physical examination		
CCS CHRS 2019 [62], CA	We recommend that a physical examination should be considered as an adjunct component of CV screening of competitive athletes (tier 2) provided it is:<br>i. Performed by an appropriately qualified professional involved in the care of athletes; and<br>ii. Followed-up as clinically warranted.		I-C
AHA ACC 2015 [3-5], CA	Before people begin training for competitive athletics, it is reasonable that they undergo careful assessment of BP, and those with initially high levels (>140 mm Hg systolic or >90 mm Hg diastolic) should have comprehensive out-of-office measurements to exclude errors in diagnosis. Ambulatory BP monitoring with proper cuff and bladder size would be the most precise means of measurement.		**I-B**
AHA ACC 2015 [3-5], CA	It is recommended that the AHA's 14-point screening guidelines and those of other societies, such as the American Academy of Pediatrics' Preparticipation Physical Evaluation, be used by examiners as part of a comprehensive history taking and physical examination to detect or raise suspicion of genetic/congenital cardiovascular abnormalities.		**I-C**
ASE 2020 [10], CA	Routine pre-participation cardiovascular screening of young competitive athletes should include a focused personal and medical history and physical examination.		C
NATA 2013 [57], CA	The task force supports recommendations from the American Academy of Family Physicians et al as the minimum standard for screening using a comprehensive personal history, family history, and physical examination.		C
NATA 2014 [1], OS	Auscultation of the heart should be performed initially with the patient in both the standing and supine positions. Auscultation should also occur during various maneuvers (eg, squat to stand, deep inspiration, Valsalva), because these maneuvers can clarify the type of murmur.		**C**
NCAA 2016 [64], CA	Although all models of cardiac screening require more research and education to improve and validate both performance and feasibility, the NCAA supports, in concept, pre-participation cardiovascular screening using a comprehensive personal and family history and physical examination, such as the American Heart Association (AHA) 14-point recommendations and/or the Pre-Participation Physical Evaluation Monograph, Fourth Edition (PPE-4).		C
ACPM 2013 [65], CA	The American College of Preventive Medicine (ACPM) supports an evaluation prior to participating in high school and collegiate sports using a standardized history and physical (H&P) (i.e., using standardized items as developed by the American Heart Association [AHA] to ensure uniformity and consistency in risk factor assessment		–
AAFP 2016 [23], CA	Illness reduction and modification evaluation of HCM, arrhythmias, coronary artery anomalies, arrhythmogenic right ventricular cardiomyopathy, and ruptured aortic aneurysm (Marfan's syndrome):<br>- Family history of premature sudden death, especially first degree relatives, and heart disease in surviving relatives<br>- Personal history of heart murmur, hypertension, excessive fatigue, syncope or near syncope with exertion, exertional chest pain, and excessive exertional shortness of breath<br>- Physical examination of pulses, heart murmurs, blood pressure, heart rhythm, and stigmata of Marfan's syndrome		–
AAP 2019 [2], OS-CA	• Physical examination should focus on detecting hypertension, pathological heart murmurs, and any physical findings suggestive of Marfan syndrome.<br>• Athletes with suspected or identified risk for SCD should be evaluated by a cardiologist, preferably a cardiologist who has experience taking care of athletes.		–
	Recommendations focussing on ECG		
EFSMA 2015 [6], CA	Recommendations for PPE in Europe: ECG at rest with 12 leads, computerized evaluation with athlete's ECG definitions if possible.		C
EFSMA 2015 [6], CA	Recommendations for PPE in Europe: ECG at rest once from 12 ys. on, before starting intensive sports or competitive sports.		–
COCIS 2021 [66-68], A	The Italian screening protocol consists of complete personal and family history, physical examination, and 12-lead ECG (Fig. 2).		–
AEPC 2017 [9], CA	The cardiovascular pre-participation screening of young athletes should include personal and family history, physical examination, and a 12-lead electrocardiogram.		–
NATA 2013 [57], CA	A resting 12-lead electrocardiogram (ECG) may be used in many preparticipation screening programs.		C
CCS CHRS 2019 [62], CA	We recommend against the "routine" performance of a 12-lead ECG for the initial CV screening of competitive athletes. "Routine" in this context is defined as "first-line" or blanket mass performance of ECG not occurring in context of an integrated program as described in Recommendation 5.		I-C

ID, Ref., Population[a]	Recommendation	LoE[b], Ref.	SORT[c]
CCS CHRS 2019 [62], CA	We recommend that a 12-lead ECG should be performed for screening of competitive athletes only when indicated according to history/questionnaire and/or physical examination (targeted screening, tier 3) and provided it is:<br>i. Of "adequate" quality. In this context "adequate" is defined as technically sufficient and of the highest possible quality;<br>ii. Interpreted by those with "appropriate expertise" ("appropriate expertise" is defined as training and skills in ECG interpretation specific to athletes and persons with disorders associated with arrhythmias) and with consideration of sport history, sex, ethnicity, age, family history, relevant clinical findings, "regional" ("regional" refers to geographic areas with a relatively high incidence of certain relevant cardiac conditions) occurrence of disease; and<br>iii. Accompanied by appropriate investigations and expert referral if clinically warranted.		I-C
ASE 2020 [10], CA	The addition of a 12-lead ECG may be considered in situations with adequate financial resources and clinical expertise.		C
ACPM 2013 [65], CA	ACPM recommends against routine screening for potential sudden cardiac death (SCD) with electrocardiogram (ECG), echocardiography, and genetic testing in individuals without personal risk factors.		–
AMSSM 2017 [8], CA	Optimally, the decision to incorporate or exclude an ECG from the preparticipation evaluation is one of shared decision-making between a patient and a provider.		–
AAP 2019 [2], OS-CA	If ECG is added to the PPE as a screening test, the most current athlete-specific criteria for ECG interpretation should be used to decrease the number of false-positive test results and minimize the additional testing, cost to the health care system, and undue stress for the individual athlete and family.		–
BSE CRY 2018 [63], CA	The 12-lead electrocardiogram should be the first investigation. (…) The ECG should be interpreted in accordance with International Consensus guidelines.		–
NCAA 2016 [64], CA	ECG screening can increase the sensitivity to detect potentially lethal cardiac conditions if physician training is improved and cardiology expertise is available. If ECG screening is used ECGs should be interpreted with modern standards that distinguish physiological changes from findings associated with pathological cardiac disorders.		C
EFSMA 2021 [7], EA	While interpreting the ECG, physicians should look out for any rhythm or conduction abnormalities, QRS morphology, abnormal axis deviation, alteration of the repolarisation and atrial enlargement.		–
	Weiterführende, nicht-invasive kardiologische Untersuchungen		
NATA 2014 [1], OS	Noninvasive cardiac testing (eg, electrocardiography [ECG], echocardiography, exercise stress testing) is not a routine aspect of the screening PPE unless warranted by findings from the personal and family history.	4 [69]	**B**
FSC 2019 [70, 71], CA	An exercise test is indicated in symptomatic athletes who plan to continue vigorous physical activity (>6 METs or sport competition).		**I-B**
FSC 2019 [70, 71], CA	An exercise test may be considered in asymptomatic athletes with a high or very high cardiovascular risk, who plan to continue vigorous physical activity (> 6 METs or competitive sports).		**IIa-C**
FSC 2019 [70, 71], CA	An exercise test is not recommended in asymptomatic athletes with a low cardiovascular risk (score <1%).		**III-C**
ESC 2022 [48], CA	In athletes with positive medical history, abnormal physical examination, or ECG alterations, further investigations including echocardiography and/or CMR to confirm (or exclude) an underlying disease are recommended.	2 [72]<br>4 [73]	**I-C**
ASE 2020 [10], CA	The use of noninvasive imaging including comprehensive and limited transthoracic echocardiography/echocardiogram, computed tomography angiography, and cardiac magnetic resonance imaging is not recommended as a first-line strategy during pre-participation cardiovascular screening.		C
EFSMA 2021 [7], EA	In competitive athletes, especially top-athletes, PPE without cardio-pulmonary exercise test (CPET) is incomplete.		–
EFSMA 2015 [6], EA	The very top elite athletes may be required to undergo a more detailed examination (IOC, FIFA, FISO) including exercise testing, echocardiography and more as indicated according to cardiology guidelines.		–
BSE CRY 2018 [63], CA	Those with 2 or more 'Borderline ECG Findings' or ANY 'Abnormal ECG Findings' require further investigation. A full standard echocardiographic assessment should be performed.		–
EFSMA 2021 [7], EA	Echocardiography is not mandatory for screening. It could be recommended for the first PPE for elite athletes when there is a justified clinical suspicion (eg, suspect ECG findings) with regard to the sports discipline with higher cardiac risk active (high static, high dynamic components).		–

ID, Ref., Population[a]	Recommendation	LoE[b], Ref.	SORT[c]
EAPC EACVI 2018 [74, 75], CA	Standard echocardiography is the first-line exam for differentiating athlete's heart from pathologic LVH.		–
EAPC EACVI 2018 [74, 75], CA	Table 1 shows the most important clinical indications to perform cardiovascular imaging in athletes.		–
EAPC EACVI 2018 [74, 75], CA	Further diagnostic work-up, instead, should be reserved to the limited subset of athletes with ECG changes potentially reflecting underlying heart disease.		–
EAPC EACVI 2018 [74, 75], CA	Exercise ECG should be the initial step in the diagnostic evaluation of athletes with suspected CAD. Individuals with inconclusive exercise ECG results could be referred for nuclear imaging.		–
EFSMA 2021 [7], EA	Indication for performing a TTE examination are only after information obtained from clinical history, physical examination and ECG at rest and during exercise test.		–
BSE CRY 2018 [63], CA	In the case of an ECG-only screening, TTE is recommended as a second-line investigation in those athletes with an abnormal ECG, cardiovascular symptoms, abnormal physical examination findings or a family history of sudden death under the age of 40 years.		–
BSE CRY 2018 [63], CA	The extent and nature of physiological cardiac adaptation in the AH is based on several factors and an attempt should be made to obtain information on each of these before the TTE is performed. This should include the list of information presented in Fig. 3 (sex, age, ethnicity, body surface area, ECG changes, symptoms, training volume, type of sport and level, family history of unexplained cardiac death <40 years).		–
BSE CRY 2018 [63], CA	The following protocols should be strictly adhered to so as to exclude pathology:<br>• BSE Minimum Dataset.<br>• BSE protocol for the assessment of LV diastolic function.<br>• BSE protocol on the assessment of the right heart with a focus on ARVC.<br>• BSE protocol for HCM.<br>• BSE protocol for DCM.<br>In addition, the following image acquisition should be made (Table 3).		–
	Further evaluations by specialists		
EAPC EACVI 2018 [74, 75], CA	Exercise stress echocardiography can be considered a very reliable and non-invasive methodology to provide information on cardiac function, contractile reserve, exercise capabilities, and arrhythmias which can be combined with clinical and ECG data and contribute to detect cardiac abnormalities.		–
EAPC EACVI 2018 [74, 75], CA	Cardiac CT should be reserved for individuals with suspected CAD (symptoms of angina, positive exercise test, arrhythmias, or syncope during exercise), aortic diseases, or pericardial pathology.		–
EAPC EACVI 2018 [74, 75], CA	In case of suboptimal echocardiographic images and contraindications to CMR, CT scan may represent the alternative imaging modality.		–
EAPC EACVI 2018 [74, 75], CA	Both photon emission computed tomography (SPECT) or positron emission tomography (PET) can be used in athletes for the assessment of myocardial ischaemia when coronary artery anomalies or disease are suspected, however ischaemia can also be related to dysbalanced myocardial perfusion (e.g. in severe LV hypertrophy) or microvascular disease		–
	Age > 35		
ESC 2021 [47], CA	Cardiac screening with family history, symptoms, physical examination, and 12-lead resting ECG should be considered for competitive athletes [age > 35].		**IIa-C**
EFSMA 2015 [6], CA	Beyond the age of 35 ys., physicians should follow the European recommendations, for pragmatic self assessment and further screening of risk patients, with stress testing. In case of abnormal findings, such as symptoms and signs and abnormal ECG, further examinations are recommended.		–
EFSMA 2015 [6], CA	Exercise testing (incl. ECG) is recommended in asymptomatic subjects before vigorous sports (males > 45 ys., females > 55 ys.). At the same time, physical capacity should be measured by exercise testing for risk estimation and evaluation of future risk.		–

[a] RA: recreational athletes, OS=participants in organized sports, A=competitive athletes, EA=elite athletes; [b] own assessment, only filled if literature could be clearly assigned;[c] bold if assigned by authors, otherwise own assessment.

Electrocardiography and echocardiography interpretation criteria

ID, Ref., Population[a]	Recommendation	LoE[b], Ref.	SORT[c]
AMSSM 2017 [8], CA	Physicians incorporating ECG in the cardiovascular screening process should optimize strategies to assure accurate ECG interpretation and adequate cardiology resources to conduct the secondary evaluation of ECG abnormalities.		–
	ECG interpretation		
COCIS 2021 [66-68], CA	Common ECG changes (Group 1) should not cause alarm and should allow eligibility to competitive sports without additional evaluation. Hence, further diagnostic investigation is needed only for the subset of athletes with uncommon (Group 2) ECG changes.		–
AEPC 2017 [9], CA	Trained physicians using the Electrocardiogram Criteria should perform the screening programme.		–
AMSSM 2017 (ECG) [76], CA	The isolated presence of high QRS voltages that fulfil voltage criteria for LVH in the absence of other ECG or clinical markers suggestive of pathology are considered part of the normal and training-related ECG changes in athletes related to physiological increases in cardiac chamber size and/or wall thickness and does not in itself require further evaluation. However, the additional presence of TWI, ST segment depression or pathological Q waves should raise the possibility of pathological LVH and should prompt further evaluation.		–
AMSSM 2017 (ECG) [76], CA	Isolated QRS voltage for RVH is part of the normal spectrum of ECG findings in athletes and in the absence of other ECG or clinical markers of pathology does not require further evaluation.		–
AMSSM 2017 (ECG) [76], CA	Incomplete RBBB represents a phenotype of cardiac adaptation to exercise and in the absence of other features suggestive of disease does not require further evaluation.		–
AMSSM 2017 (ECG) [76], CA	All patterns of early repolarisation, when present in isolation and without clinical markers of pathology, should be considered benign variants in athletes.	3 [77]	–
AMSSM 2017 (ECG) [76], CA	TWI in leads V1-V4 when preceded by J-point elevation and convex ST segment elevation should be considered part of the 'black athlete's heart' and should not result in further investigation, in the absence of other clinical or ECG features of cardiomyopathy.		–
AMSSM 2017 (ECG) [76], CA	TWI in the anterior leads (V1-V3) in adolescent athletes <16 years of age (or prepubertal athletes) should not prompt further evaluation in the absence of symptoms, signs or a family history of cardiac disease.		–
AMSSM 2017 (ECG) [76], CA	In the absence of symptoms such as fatigue, dizziness, or syncope, heart rates ≥30 bpm are considered normal in highly trained athletes.		–
AMSSM 2017 (ECG) [76], CA	Sinus arrhythmia, the physiological fluctuation in heart rate with breathing, is considered a normal finding and should not be confused with sinus node dysfunction or sick sinus syndrome. Differentiating features that suggest sinus node dysfunction include:<br>► lack of rhythmic changes in the heart rate,<br>► abrupt sustained rate increases and decreases,<br>► prolonged pauses or periods of sinus arrest,<br>► inappropriate rate response to exercise (including slowed acceleration and an inappropriately rapid deceleration),<br>► any association with clinical symptoms such as exercise intolerance, pre-syncope and syncope.		–
AMSSM 2017 (ECG) [76], CA	Left axis deviation, left atrial enlargement, right axis deviation, right atrial enlargement and complete RBBB are considered borderline variants in athletes. The presence of any one of these findings in isolation or with other recognised physiological electrical patterns of athletic training does not warrant further assessment in asymptomatic athletes without a family history of premature cardiac disease or SCD. Conversely, the presence of more than one of these borderline findings in combination places the athlete in the abnormal category warranting additional investigation		–
AMSSM 2017 (ECG) [76], CA	Abnormal TWI in asymptomatic athletes warrants a comprehensive clinical assessment to exclude underlying cardiomyopathy.		–
AMSSM 2017 (ECG) [76], CA	TWI affecting the lateral leads (V5-V6, I and aVL) is considered abnormal and should prompt comprehensive investigation irrespective of ethnicity, including cardiac MRI, when echocardiography is non-diagnostic.		–
AMSSM 2017 (ECG) [76], CA	Cardiac MRI should be a standard component of the assessment for markedly abnormal ECGs suggestive of apical HCM, specifically ECGs with deep (>–0.2 mV) TWI and ST segment depression in the lateral or inferolateral leads.		–

ID, Ref., Population[a]	Recommendation	LoE[b], Ref.	SORT[c]
AMSSM 2017 (ECG) [76], CA	Anterior TWI is a normal variant in asymptomatic adolescent athletes age <16 years and in black athletes when preceded by J-point elevation and convex ST segment elevation.		–
AMSSM 2017 (ECG) [76], CA	In most non-black athletes age ≥16 years, anterior TWI beyond lead V2 should prompt further evaluation given the potential overlap with ARVC.	4 [78]	–
AMSSM 2017 (ECG) [76], CA	In athletes age ≥16 years with TWI beyond V2, concurrent findings of J-point elevation, ST segment elevation or biphasic T waves more likely represent athlete's heart, while the absence of J-point elevation or a coexistent depressed ST segment is more concerning for ARVC.84Other ECG findings suggestive of ARVC in the presence of anterior TWI include low limb lead voltages, prolonged S wave upstroke, ventricular ectopy with LBBB morphology and epsilon waves.	4 [79]	–
AMSSM 2017 (ECG) [76], CA	ST segment depression (relative to the isoelectric PR segment) in excess of 0.05 mV (0.5 mm) in two or more leads should be considered an abnormal finding requiring definitive evaluation for underlying structural heart disease		–
AMSSM 2017 (ECG) [76], CA	The consensus of this panel based on existing scientific data is to modify the definition for pathological Q waves in athletes as a Q/R ratio ≥0.25 or ≥40 ms in duration in two or more contiguous leads (except III and aVR).		–
AMSSM 2017 (ECG) [76], CA	Athletes with complete LBBB require a thorough investigation for myocardial disease including echocardiography and a cardiac MRI with perfusion study.		–
AMSSM 2017 (ECG) [76], CA	In asymptomatic athletes with profound non-specific intraventricular conduction delay, an echocardiogram is recommended to evaluate for myocardial disease.		–
AMSSM 2017 (ECG) [76], CA	Evaluation of epsilon waves, especially in combination with right precordial TWI or delayed S wave upstroke, requires the exclusion of possible ARVC through a combination of tests including echocardiography, cardiac MRI, Holter monitoring, exercise ECG test and signal averaged ECG.		–
AMSSM 2017 (ECG) [76], CA	Asymptomatic athletes with WPW pattern should be investigated for the presence of a low-risk or high-risk accessory pathway. Non-invasive risk stratification begins with an exercise stress test in which abrupt, complete loss of pre-excitation at higher heart rates suggests a low-risk accessory pathway. If non-invasive testing cannot confirm a low-risk pathway or is inconclusive, electrophysiology testing should be considered	3 [80]<br>4 [81]	–
AMSSM 2017 (ECG) [76], CA	This consensus group also recommends QTc values of >470 ms in males and >480 ms in females to define the threshold of QT prolongation that warrants further assessment in asymptomatic athletes. It is critical that an athlete with a single QTc reading above these threshold values not be obligated a diagnosis of LQTS, but rather that these cut-off values have triggered the need for additional evaluation.		–
AMSSM 2017 (ECG) [76], CA	Genetic testing for LQTS is recommended for any athlete where a cardiologist has an index of suspicion for LQTS (intermediate or high probability score), or for an asymptomatic patient with no family history but an incidental ECG finding with a QTc >480 ms pre-puberty and >500 ms post-puberty that is confirmed on repeat ECG testing.		–
AMSSM 2017 (ECG) [76], CA	The coved ST segment elevation in type 1 Brugada pattern results in a broad r' and should be distinguishable from the upsloping ST segment elevation of early repolarisation in an athlete.		–
AMSSM 2017 (ECG) [76], CA	Patients with a [Brugada] type 1 ECG pattern should be referred to a cardiac electrophysiologist for further evaluation, regardless of symptoms.		–
AMSSM 2017 (ECG) [76], CA	A resting heart rate ≤30 bpm or a sinus pause ≥3 s may be normal in a well-trained athlete but nevertheless should prompt further evaluation.		–
AMSSM 2017 (ECG) [76], CA	Mobitz type II second-degree AV block and third-degree (complete) AV block are pathological disruptions<br>in AV conduction and abnormal findings in athletes.		–
AMSSM 2017 (ECG) [76], CA	In athletes with ≥2000 PVCs per 24 hours or with episodes of non-sustained ventricular tachycardia, or with an increasing burden of ectopy during an incremental exercise test, additional evaluation may include contrast-enhanced cardiac MRI and more invasive EP study.	4 [82]	–
AMSSM 2017 (ECG) [76], CA	SVT, atrial fibrillation and atrial flutter are rarely seen on a resting ECG in athletes and require investigation.		–

ID, Ref., Population[a]	Recommendation	LoE[b], Ref.	SORT [c]
AMSSM 2017 (ECG) [76], CA	Ventricular couplets, triplets and non-sustained ventricular tachycardia always require investigation.		–
AMSSM 2017 (ECG) [76], CA	[in athletes ≥30 years of age] Additional evaluation for underlying coronary artery disease should be considered in asymptomatic older athletes with TWI, pathological Q waves, ST segment depression, left or right bundle branch block, abnormal R wave progression, left anterior hemiblock and atrial fibrillation.		–
AMSSM 2017 (ECG) [76], CA	Several common heritable cardiomyopathies including HCM, ARVC and familial DCM may present with ECG abnormalities prior to the onset of overt heart muscle pathology. Therefore, athletes with abnormal ECGs suggestive of cardiomyopathy and initially normal clinical evaluations should be followed with serial evaluation during and after their competitive athletic careers.		–
EAPC EACVI 2018 [74, 75], CA	The ECG should be evaluated in relation with the athlete's gender, age and race, family history of cardiovascular disease and/or SCD, clinical symptoms, physical examination, and intensity/duration of physical exercise.		–
EAPC EACVI 2018 [74, 75], CA	These [athlete's heart] ECG abnormalities should be clearly separated from training unrelated ECG patterns (present in <5%), such as ST-segment depression and T-wave inversion, pathologic Q waves, major intraventricular conduction defects, ventricular pre-excitation, long or short QT interval, and ventricular arrhythmias, which may be an expression of cardiovascular disorders, notably cardiomyopathies and cardiac ion channel diseases, with potential risk of SCD during sports.		–
EAPC EACVI 2018 [74, 75], CA	Some borderline ECG variants (left and right atrial enlargement, left and right axis deviation, and right ventricular hypertrophy) are considered of uncertain significance in athletes and, in the setting of cardiac evaluation, should not require additional investigation if not associated with positive family history and present in isolation.	2 [83]	–
EAPC EACVI 2018 [74, 75], CA	In asymptomatic athletes with a negative family history, ECG changes due to cardiac adaptation to physical exertion should not cause alarm and do not represent indication for additional evaluation.		–
EHRA EACPR 2017 [46], OS-CA	The updated recommendations for interpretation of the athlete's ECG (Seattle criteria) represent a useful document to the scope.		–
	Echocardiogram interpretation		
EAPC EACVI 2018 [74, 75], EA	In elite athletes the LV end-diastolic diameter is not frequently increased >60 mm. A LV end-diastolic diameter >60 mm— when combined with reduced EF and abnormal diastolic function—should raise suspicion of IDCM.		–
EAPC EACVI 2018 [74, 75], EA	In elite athletes LVH involves typically all myocardial segments and the maximal septal thickness is usually ≤12 mm. Septal wall thickness is lower in female athletes and more pronounced in African than in Caucasian athletes.		–
EAPC EACVI 2018 [74, 75], CA	In HCM increased wall thickness (>15 mm) involves mainly the basal septum and in 20% of HCM cases is associated with additional features, such as SAM or aortic valve mid-systolic closure.		–
EAPC EACVI 2018 [74, 75], CA	After physical deconditioning of three months, a reduction of LV wall thickness can be observed in athletes but not in HCM.		–
EAPC EACVI 2018 [74, 75], CA	In athletes LVH is combined with normal EF, normal, or even increased SV and s' velocity >9 cm/s, whereas s' is <9 cm/s in HCM. EF is normal or high in early stages and possibly reduced in advanced stages of HCM.		–
EAPC EACVI 2018 [74, 75], CA	LV diastolic function is often supranormal in athletes (E/A ratio >2, increased e' velocity, low E/e' ratio). In the HCM E/A ratio is <1, E velocity deceleration time is prolonged, e' velocity and e'/a' ratio are low. However, normal LV diastolic filling pattern does not exclude pathological LVH.		–
BSE CRY 2018 [63], CA	Regardless of whether TTE is a first- or second-line investigation, it should be performed according to the BSE Minimum Dataset for a Standard Transthoracic Echocardiogram in an Adult and should also consider recommendations made in the Supplementary Protocols for (i) Comprehensive Assessment of the Right Heart and (ii) the Assessment of Diastolic Function.		–
BSE CRY 2018 [63], CA	LV geometry should be determined using a combination of LV mass indexed to BSA (LVMI) and relative wall thickness (RWT). LVMI is calculated as per BSE guidelines and RWT is calculated by summating septal and posterior wall thickness in diastole and dividing into the LV diastolic cavity dimension. LV geometry can be reported as 'normal' (normal RWT and normal LVMI), 'concentric remodeling'	4 [84]	–

ID, Ref., Population[a]	Recommendation	LoE[b], Ref.	SORT[c]
	(increased RWT with normal LVMI), 'concentric hypertrophy' (increased RWT and increased LVMI) or 'eccentric hypertrophy' (normal RWT with increased LVMI) according to published criteria (Fig. 4)		

[a] RA: recreational athletes, OS=participants in organized sports, A=competitive athletes, EA=elite athletes; [b] own assessment, only filled if literature could be clearly assigned;[c] bold if assigned by authors, otherwise own assessment.

Pneumology

ID, Ref., Population[a]	Recommendation	LoE[b], Ref.	SORT[c]
NATA 2012 [45], OS	Athletes who may have or are suspected of having asthma should undergo a thorough medical history and physical examination.		**B**
AAP 2019 [2], OS-CA	Spirometry at rest followed by a challenge test to elicit bronchoconstriction is the preferred method to diagnose EIB with or without asthma.	2 [85]<br>4 [86]	**A**
AAP 2019 [2], OS-CA	Pulmonary evaluation of the anterior and posterior chest should be done in a quiet room with the patient in the seated position using breaths that are deeper than those of normal breathing.		–

[a] RA: recreational athletes, OS=participants in organized sports, A=competitive athletes, EA=elite athletes; [b] own assessment, only filled if literature could be clearly assigned;[c] bold if assigned by authors, otherwise own assessment.

Internal medicine (further topics)

ID, Ref., Population[a]	Recommendation	LoE[b], Ref.	SORT[c]
NATA 2014 [1], OS	The use of routine laboratory or other screening tests such as urinalysis, complete blood count, chemistry profile, lipid profile, ferritin level, or spirometry during the PPE is not supported by current studies.	4 [87]<br>4 [88]	**B**
NATA 2014 [1], OS	If the athlete has a history of anemia, then hemoglobin and ferritin levels should be measured.	4 [89]<br>4 [90]	**C**
NATA 2014 [1], OS	Lipid profiles should be reserved for those who have a personal history of elevated cholesterol or dyslipidemia and those athletes in whom other cardiovascular risk factors have been identified (by history or examination) that require further investigation as part of a thorough medical evaluation rather than as part of the PPE.	2 [91]	**C**
NATA 2014 [1], OS	For athletes with a history of elevated cholesterol or lipid levels, longitudinal care by the team physician includes review of previous laboratory results and appropriate management.		**C**
EFSMA 2021 [7], EA	additional blood (haematological and biochemical tests depending on the athlete's age and level of training) and urine samples are collected.		–
EFSMA 2021 [7], EA	The athlete blood test panel should include the complete blood cell count and a comprehensive metabolic panel (glucose blood level, liver panel, kidney profile, lipid profile, electrolytes, vitamin D). When the three diagnostic components of the PPE are completed, the SEM doctor can ask for more biomarkers to be tested to make the right diagnosis (if there is a suspicion of nutritional deficiencies, overtraining, relative energy deficiency in sport (RED-S), hormonal imbalances, inefficient recovery after injury, etc).		–
	Chronic infections		
AAP 2019 [2], OS-CA	While mandatory testing of athletes for HIV or hepatitis is not recommended, voluntary testing should be encouraged for athletes at high risk, that is, who have exposure to blood products, symptoms suggestive of disease, or significant risk factors detected during the PPE.		–
	Sickle cell trait		
NATA 2012 [45], OS	Screening for sickle cell trait (SCT), by self-report, is a standard component of the preparticipation physical evaluation (PPE) monograph. Testing for SCT, when included in the PPE or conducted previously, confirms SCT status.		**A**
NATA 2014 [1], OS	Confirmatory testing is recommended for those athletes who report a history of sickle cell trait and those whose family heritage suggests higher risk. Athletes found to have sickle cell trait should be educated by the medical staff and be monitored carefully for heat- and dehydration- related concerns during training and competition.		**C**
NATA 2013 [57], CA	Efforts to obtain newborn screening results of sickle cell trait (SCT) status during the preparticipation physical evaluation are recommended.		C

ID, Ref., Population[a]	Recommendation	LoE[b], Ref.	SORT[c]
NATA 2013 [57], CA	In the absence of newborn screening results, SCT screening during the preparticipation physical evaluation should be considered for all athletes, especially if they are performing intense physical activity.		C
AAP 2019 [2], OS-CA	Universal [sickle cell trait] screening is not widely recommended for athletes, except for NCAA athletes who are required as students to show proof of screening or decline screening.		–

[a] RA: recreational athletes, OS=participants in organized sports, A=competitive athletes, EA=elite athletes; [b] own assessment, only filled if literature could be clearly assigned;[c] bold if assigned by authors, otherwise own assessment.

Orthopaedics

ID, Ref., Population[a]	Recommendation	LoE[b], Ref.	SORT[c]
NATA 2014 [1], OS	The musculoskeletal history screening and examination can be combined for asymptomatic athletes with no previous injuries (Table 4). With an accurate history, the clinician can detect more than 90% of significant musculoskeletal injuries; the screening physical examination is 51% sensitive and 97% specific. If the player has either a previous injury or other signs or symptoms (eg, pain or tenderness; asymmetric muscle bulk, strength, or range of motion; or any obvious deformity) detected during the general screening examination or history, the relevant elements of a site-specific examination should be performed.	2 [92]	**A**
AAP 2019 [2], OS-CA	Key findings during the musculoskeletal history will direct the focus of the musculoskeletal physical examination, as there are currently limited screening examinations that have been validated to reduce injury risk.		**B**
EFSMA 2021 [7], EA	The musculoskeletal manual screening combines both history and physical examination, to be informed about previous injuries in asymptomatic athletes. [...] This can be detected during the examination or history, the relevant elements of a site-specific examination should be performed.		–
EFSMA 2021 [7], EA	Physicians can also focus on movement quality. This involves identification and rating functional compensations, asymmetries, impairments or efficiency of movement control through transitional or dynamic movement.		–
AAP 2019 [2], OS-CA	A general [musculoskeletal] screening examination is reasonable for athletes with no symptoms and no previous injury.		–
AAP 2019 [2], OS-CA	Examiners need to determine which method best suits a given situation, depending on history of injury, musculoskeletal signs or symptoms, resources and time available, and type of sport or activity in which the athlete will participate.		–
	Medical history		
NATA 2014 [1], OS	Musculoskeletal injury is a common cause for restriction or disqualification of an athlete, so the medical history should attempt to detect any underlying condition that might predispose an athlete to injury. Special attention in the examination should be given to any areas that have been injured or undergone surgery.	4 [93]	**B**
	Spine		
AAP 2019 [2], OS-CA	The cervical spine should be inspected for posture and alignment. The ear canal should line up with the middle of the shoulder. Forward flexion of the neck should allow the chin to touch the manubrium, extension should allow a nearly vertical gaze, rotation should let the chin almost touch the clavicle in both directions, and the ears should approach the shoulders with lateral flexion. Any asymmetrical or deficient motion should be noted. Examination of the thoracolumbar spine and back focuses on posture,<br>range of motion, and potential deformities. The scapulae should be level, symmetrical, and flat against the thoracic cage. The presence of scoliosis, kyphosis at the thoracic level, or lordosis at the lumbar level should be documented. Scoliosis causes a rotatory deformity as the athlete bends forward at the waist (Figure 6G-2). Pain or restriction of forward flexion may indicate lumbar disk disease. Pain from compression fractures is most often midline, is most often worse with flexion of the spine, and may be related to neurological findings. Back extension may increase pain from a facet injury, spondylolysis, spondylolisthesis, or a sprain or strain. As part of the thoracolumbar examination, the athlete bends forward at the waist. The rotary deformities of scoliosis, such as asymmetrical, prominent ribs; curvature of the spine; or an asymmetrical waist, are accentuated in this position.		–
	Shoulder		
AAFP 2016 [23], CA	Injury Reduction and Modification Evaluation (The ‘‘Disabled Throwing Shoulder’’ (DTS)):<br>- History of any previous injury in the shoulder and relevant parts of the kinetic chain.		–

ID, Ref., Population[a]	Recommendation	LoE[b], Ref.	SORT[c]
	- Volume and intensity of training<br>- Examination of shoulder, including the scapula and kinetic chain<br>- When possible, coordinate the evaluation of the throwing, hitting, or serving mechanics, including the kinetic chain.		
AAP 2019 [2], OS-CA	The shoulder examination begins with inspection for symmetry with the athlete standing. It is important to visualize the posterior and anterior aspects of the shoulder boney and muscular anatomies (for bruising, scapular symmetry, winging, atrophy, acromioclavicular joint prominence, and sternoclavicular joint prominence). Palpate the bilateral sternoclavicular joint, acromioclavicular joint, and proximal biceps tendon and bicipital groove. Range of motion in abduction (Figure 6G-3A), flexion (Figure 6G-3B), and internal rotation (Figure 6G-3C) and external rotation (Figure 6G-3D) are then assessed.	–	
AAP 2019 [2], OS-CA	Shoulder impingement signs should be tested with Neer impingement test and Hawkins test.	–	
AAP 2019 [2], OS-CA	A screening for multidirectional instability includes subluxation tests in the anterior and posterior planes of a supine athlete (Figures 6G-6A–6G-6C) and in the inferior plane of a seated athlete.	–	
	Elbow, wrist		
AAFP 2016 [23], CA	Injury Reduction and Modification Evaluation (Elbow):<br>- Review of playing and throwing history to determine risk factors<br>- History of elbow, shoulder, and kinetic chain injury and rehabilitation status<br>- Examination of the elbow, including the shoulder and kinetic chain<br>- When possible, coordinate the evaluation of the throwing, hitting or serving mechanics, including the kinetic chain.	–	
AAP 2019 [2], OS-CA	The elbow is observed for swelling, discoloration, and carrying angle (cubital valgus). The elbow should extend fully and then flex to allow the athlete to touch the ipsilateral shoulder with the hand.	–	
AAP 2019 [2], OS-CA	Forearm motion is assessed by having the athlete pronate and supinate the forearms with the elbows bent 90 degrees at his or her sides. The athlete should be able to turn the hand completely palm up and completely palm down.	–	
AAP 2019 [2], OS-CA	In a throwing athlete, medial stability can be assessed by applying a valgus force to the elbow (Figure 6G-7B). The modified milking maneuver (Figure 6G-7C) also assesses medial instability. The test result is considered positive if pain is noted over the ulnar collateral ligament or if the joint opens medially. Direct comparison with the opposite arm is critical.	–	
AAP 2019 [2], OS-CA	The hand and wrist should be evaluated for symmetry. Wrists should palmar flex equally to about 80 degrees and dorsiflex to 70 degrees or more. There should be more ulnar deviation than radial deviation. The fingers should be able to close into a full fist, and each fingernail should point at the scaphoid bone with the fingers flexed across the palm.	–	
	Hip		
AAP 2019 [2], OS-CA	The hip examination begins with observation of the standing posture. The iliac crest and posterior superior iliac spine heights should be level with the floor when the torso is aligned symmetrically, and the athlete should be able to stand on each foot without any translation or tilting of the pelvis. The hip joint should be palpated for tenderness in the greater tuberosities, hip flexor, adductor, and external rotator tendons (behind the greater trochanter).<br>Hip range of motion can be assessed with the athlete lying supine. Landmarks should be reviewed at this point. With the hip and the knee fully extended, the hip joint is rotated internally and externally (a “log roll” movement), and any asymmetry is noted. Symmetry of abduction and adduction should also be observed. Hip flexion should be beyond 90 degrees, and the knees should come straight toward the chest; any external rotation indicates an intrinsic hip deformity. With the hip and knee flexed to 90 degrees, the hip joint should have 40 degrees of internal rotation and 45 degrees of external rotation.<br>Keeping the athlete’s hip flexed 90 degrees and extending the knee checks hamstring flexibility. The popliteal angle should be 0 degrees to 10 degrees in young children and can vary depending on patient age and sex.	–	
AAP 2019 [2], OS-CA	Passive assessment of the hip in flexion, adduction and internal rotation (FADIR) is conducted as part of the supine assessment of the hip.	–	
AAP 2019 [2], OS-CA	The FABER test involves combining the motion of hip flexion, abduction, and external rotation. Evaluation documents pain provocationand range of motion. Posterior hip pain may be indicative of sacroiliac joint involvement, while anterior hip pain or groin pain indicates intra-articular hip pathology.	–	

ID, Ref., Population[a]	Recommendation	LoE[b], Ref.	SORT[c]
	With the athlete standing, inspection should reveal a normal leg-thigh valgus angulation of 12 degrees or less in males and 18 degrees or less in females. The patella should be observed for abnormal lateral subluxation or tilt or an excessively high position (patella alta) with the athlete seated. A patella apprehension test evaluates for patella instability and a positive test result is apprehension with lateral translation of the patella and the knee in extension.		
	Knee		
AAFP 2016 [23], CA	Injury Reduction and Modification Evaluation (Knee Injuries: ACL): Athletes in running, landing, and cutting sports should have an evaluation (10), including:<br>o History of previous personal or family ACL injury<br>o Lower extremity alignment (e.g., knee valgus)<br>o Motor control (including core and lower extremity strength, balance and flexibility)<br>Other evaluation techniques may include:<br>o Review of training surface and shoe type<br>o Screening biomechanical analysis of jumping and landing		–
AAFP 2016 [23], CA	Injury Reduction and Modification Evaluation (Other knee injuries): Athletes should have an evaluation (10), including:<br>o History of previous lower extremity injury and rehabilitation<br>o Present and anticipated volume of training and participation<br>o Patellar and peri-patellar examination<br>o Lower extremity alignment (e.g., femoral anteversion, knee valgus, foot pronation)<br>o Hip abduction, quadriceps, and hamstring strength<br>o Quadriceps, hamstring, and iliotibial band flexibility Other evaluation techniques may include the following:<br>o Review of training surface and shoe type<br>o Screening biomechanical analysis of jumping and landing		–
AAP 2019 [2], OS-CA	The remainder of the knee examination should be done with the athlete supine. Each patella should be evaluated for hypermobility by translating the patella medially and laterally with the knee in approximately 20 degrees of flexion; comparison with the opposite side should be made. Joint-line tenderness may indicate a meniscal tear. Any amount of knee effusion should be noted. Knee range of motion should be from full extension or hyperextension to approximately 140 degrees of flexion. Knee ligament stability tests include the Lachman test for ACL deficiency (Figure 6G-12A), posterior drawer test for posterior cruciate ligament insufficiency (Figures 6G-12B and 6G-12C), and varus and valgus stress tests for collateral ligament laxities (Figures 6G-12D and 6G-12E). It is important to remember that a negative anterior drawer test result does not rule out an ACL tear and the Lachman test is the better test for ACL integrity. Palpation of the tibial tubercle is important to assess for Osgood-Schlatter disease (tibial tubercle apophysitis).		–
	Lower leg, ankle, foot		
AAFP 2016 [23], CA	Injury Risk Reduction and Modification Evaluation (Inversion Ankle Sprains): Athletes in running, landing, and cutting sports should have an evaluation, including the following:<br>o History of previous ankle injury<br>o Ankle ligament evaluation<br>o Heel alignment<br>o Ankle muscle strength and flexibility testing<br>o Balance and core muscle control<br>o Body mass index (BMI)		–
AAP 2019 [2], OS-CA	The lower leg and tibia examination should include the shin edema test (SET) and shin palpation test (SPT) to test for medial tibial stress syndrome (MTSS). SET is sustained palpitation of the distal two-thirds of the medial surface of the tibiae bilaterally for at least 5 seconds to evaluate for signs of pitting edema. SPT is palpation of the distal two-thirds of the posteromedial border of the tibiae for focal bony tenderness that may indicate bone stress injuries.	3 [94]	–
AAP 2019 [2], OS-CA	The ankles are evaluated with the athlete standing and sitting for normal appearance. In the seated position, active dorsiflexion to 20 degrees and plantar flexion to 40 degrees should be present. With the knee extended, tightness in the Achilles tendon can be assessed by passively dorsiflexing the seated athlete's ankle while observing the lateral aspect of the leg and ankle. The ankle should dorsiflex 15 degrees to 20 degrees past neutral. Stress testing for ligament laxity includes the anterior drawer test for anterior subluxation and the talar tilt test for lateral ligament stability.		–
AAP 2019 [2], OS-CA	At inspection of the foot, pes cavus or rigid flatfoot deformities should be noted. A supple flatfoot does not affect an athlete's performance, but it may be a risk factor for upstream problems in the kinetic chain, such as MTSS or patellofemoral joint pain.		–

ID, Ref., Population[a]	Recommendation	LoE[b], Ref.	SORT[c]
AAP 2019 [2], OS-CA	A simple screening for potential lower extremity injury risk is the single-leg squat (SLS) test. Barefoot athletes are asked to place their hands onto their hips and stand on one limb and flex the opposing limb to 90 degrees, followed by an SLS to 30 degrees of knee flexion with return to a fully extended knee position. Abnormal responses, which include arms flailing, Trendelenburg sign, or collapse of the supporting knee into valgus, should be noted (Figure 6G-14).	3 [95]	–
AAP 2019 [2], OS-CA	The data supporting functional movement testing and improved outcomes are not strong. Testing should be considered in players of lateral, pivoting, cutting sports.<br>Athletes in sports requiring lateral, pivoting, and cutting motions might be evaluated in greater detail with the box drop test and core strength evaluation to determine deficits in neuromuscular control that increase risk of ACL rupture.	2 [96]<br>3 [97]	–
	Athletes		
AAP 2019 [2], OS-CA	Screening for biomechanical risk factors associated with higher rates of ACL injury can be performed as part of the PPE, especially for female athletes participating in pivoting and cutting sports such as soccer, basketball, and team handball.		–
AAFP 2017 [27], CA	It is desirable the team physician identify risk factors [for ACL injuries] during the PPE.		–
AAFP 2017 [27], CA	It is desirable the team physician utilize the PPE to identify and address known risk factors for the development of patellofemoral pain.		–
AAFP 2017 [27], CA	It is desirable the team physician (…) identify musculoskeletal deficits as the basis for a conditioning program		–
AAFP 2017 [27], CA	It is desirable the team physician (…) perform a comprehensive musculoskeletal kinetic chain evaluation of the athlete [with regard to shoulder injuries]		–
	Athletes with disabilities		
AAP 2019 [2], OS-CA	The musculoskeletal examination of an athlete who uses a wheelchair should include evaluation of the stability, flexibility, and strength of commonly injured sites (eg, shoulder, hand, and wrist) and the trunk.		–
AAP 2019 [2], OS-CA	Athletes with lower-limb amputation and prostheses require a full assessment of the lower back, pelvis, and lower extremities, and those with upper-limb amputation and prosthetic devices require a full assessment of the upper back, shoulder girdle, and upper extremities.		–
AAP 2019 [2], OS-CA	Athletes with cerebral palsy have decreased strength, decreased musculotendinous flexibility (often with contractures), and muscle strength imbalances, especially of the lower extremities. These conditions vary in severity from mild and nearly imperceptible to very severe and requiring wheelchair use.<br>Overuse injuries, strains, and sprains are common, especially at the hips, knees, ankles, and feet. The PPE should include a thorough examination of these regions.		–

[a] RA: recreational athletes, OS=participants in organized sports, A=competitive athletes, EA=elite athletes; [b] own assessment, only filled if literature could be clearly assigned;[c] bold if assigned by authors, otherwise own assessment.

Neurology

ID, Ref., Population[a]	Recommendation	LoE[b], Ref.	SORT[c]
NATA 2014 [1], OS	If the athlete has a history of concussion, seizure disorder, cervical spine stenosis, or spinal cord injury, a thorough neurologic assessment is necessary.		**C**
AAFP 2016 [23], CA	Injury Reduction and Modification Evaluation (Cervical spine injury): Athletes should have an evaluation including:<br>o History of c-spine injury or abnormality<br>o C-spine and neurological examination<br>o Consideration of additional testing and/or consultation		–
EFSMA 2021 [7], EA	A thorough neurological assessment is necessary if the athlete has a history of concussion, seizure disorder, cervical spine stenosis or spinal cord injury. This is of special significance for soccer players and rugby or American football to evaluate the risk of cerebral harms.		–
	Athletes with disabilities		
AAP 2019 [2], OS-CA	Since many athletes with physical impairments may also have some form of neurological deficit, a complete neurological evaluation should be performed.		–
	Cancer survivors		

ID, Ref., Population[a]	Recommendation	LoE[b], Ref.	SORT[c]
ACSM 2019 [16], RA	Older survivors and/or survivors treated with neurotoxic chemotherapy (typical for breast, colon, lung, ovarian cancers) may especially benefit from a standard assessment of balance and mobility to assess fall risk.	1 [98]	C

[a] RA: recreational athletes, OS=participants in organized sports, A=competitive athletes, EA=elite athletes; [b] own assessment, only filled if literature could be clearly assigned;[c] bold if assigned by authors, otherwise own assessment.

Psychiatry

ID, Ref., Population[a]	Recommendation	LoE[b], Ref.	SORT[c]
NATA 2015 [99], CA	The Plan [for Recognition and Referral of Student-Athletes With Psychological Concerns][d] offers questions regarding a student-athlete's history of a mental health concern or present psychological status at the preparticipation physical examination, with follow-up questionnaires if the student-athlete's answers indicate the need for further evaluation.		**B, C**
NATA 2015 [99], CA	The Plan [for Recognition and Referral of Student-Athletes With Psychological Concerns][d] provides questions to consider asking when approaching a student-athlete with a potential psychological concern.		**B, C**
NATA 2014 [1], OS	As part of the health history portion of the PPE, including questions to determine the mental health status of the athlete should be considered, along with a plan for referral and follow-up where appropriate.	4 [100]	**C**
AAP 2019 [2], OS-CA	As part of the health history portion of the PPE, questions addressing the mental health status of the athlete should be considered, along with a plan for referral and follow-up.		–
AAP 2019 [2], OS-CA	While screening tools are not validated as stand-alone assessments for mental disorders, they may be incorporated into the PPE as indicated.		–

[a] RA: recreational athletes, OS=participants in organized sports, A=competitive athletes, EA=elite athletes; [b] own assessment, only filled if literature could be clearly assigned;[c] bold if assigned by authors, otherwise own assessment.

Advice for athletes

ID, Ref., Population[a]	Recommendation	LoE[b], Ref.	SORT[c]
	Cardiovascular risks		
AMSSM 2017 [8], CA	Part of the PPE process should include athlete and family education on cardiovascular signs and symptoms that may develop after the examination and warrant re-evaluation.		–
	Acute infection		
AEPC 2017 [9], CA	The screening programme should include instructing athletes to suspend exercise during infections and recognise red flag signs.		–
	Heat and hdydration		
AAP 2019 [2], OS-CA	Educating athletes, parents or guardians, and coaches about risk factors and preventive strategies is key to decreasing the incidence of heat illness.		–
AAP 2019 [2], OS-CA	Athletes should be informed of the increased heat intolerance caused by some medications and supplements. The use of diuretics, caffeine, antihistamines, or stimulants increases the risk of heat illness.<br>Banned substances such as ephedra or methamphetamines also increase the risk of heat injury.		–
	Pregnancy		
ACSM 2021 [11], RA	All pregnant women should be educated on the warning signs for when to stop exercise.		–

[a] RA: recreational athletes, OS=participants in organized sports, A=competitive athletes, EA=elite athletes; [b] own assessment, only filled if literature could be clearly assigned;[c] bold if assigned by authors, otherwise own assessment.

Clearance

ID, Ref., Population[a]	Recommendation	LoE[b], Ref.	SORT[c]
OS-CA	When considering any abnormality or condition found during the PPE that may influence participation, the practitioner should consider the following questions:<br>— Does participation put the athlete at risk for illness or injury above the inherent hazards of the		–

ID, Ref., Population[a]	Recommendation	LoE[b], Ref.	SORT[c]
	activity?<br>— Does participation increase the risk of injury or illness for other participants?<br>— Will treatment of the underlying condition allow safe participation (medication, rehabilitation, bracing, and padding)?<br>— Can limited participation be allowed while treatment or evaluation is completed?<br>— If medical eligibility is denied for certain sports because of medical or safety concerns, can the athlete safely participate in other activities or sports?		
	RED-S		
IOC 2018 [29, 30], CA	It is recommended that athletes in the 'High Risk—Red Light' risk category should not be cleared to participate in sport.		–
IOC 2018 [29, 30], CA	Athletes in the 'Moderate Risk— Yellow Light' risk category should be cleared for sport participation only with supervised participation and a medical treatment plan.		–
	Fe(male) athlete trias		
FMATC 2021 [32, 33], CA	Risk assessment tools can be helpful in guiding clearance and return-to-play decisions in the male athlete with one or more components of the Male Athlete Triad.		**B**
	cardio metabolic		
CCS CHRS 2019 [62], CA	We recommend that sport restriction be considered and discussed in the following conditions: ARVC, exercise-induced significant ventricular arrhythmias (if arrhythmogenic risk cannot be mitigated), catecholaminergic polymorphic ventricular tachycardia, exercise-induced heart block, hypertrophic cardiomyopathy with sustained ventricular tachycardia or multiple risk factors, dilated cardiomyopathy unrelated to an athlete's heart, left ventricular non-compaction with left ventricular dysfunction and or ventricular arrhythmia, Marfan syndrome with aortic dilatation, significant aortic dilatation, coronary artery aneurysm with ischemia, oral anticoagulation treatment in an athlete competing in sports with a high risk of injury causing bleeding, pulmonary hypertension, and cyanotic congenital heart disease.		I-C
AAP 2019 [2], OS-CA	Athletes identified with cardiovascular symptoms or signs such as exertional syncope or near syncope, chest pain, palpitations, or excessive exertional dyspnea require a thorough cardiovascular evaluation to exclude underlying heart disease before they are allowed to participate in vigorous physical activity.		–
	Athletes with disabilities		
AAP 2019 [2], OS-CA	Medical eligibility for sports participation should follow the same principles used for athletes with no disability (see Chapter 5). The emphasis in this population of athletes is on inclusion and safe participation.		–

[a] RA: recreational athletes, OS=participants in organized sports, A=competitive athletes, EA=elite athletes; [b] own assessment, only filled if literature could be clearly assigned;[c] bold if assigned by authors, otherwise own assessment.

References

1. Conley KM, Bolin DJ, Carek PJ, Konin JG, Neal TL, Violette D. National Athletic Trainers' Association position statement: Preparticipation physical examinations and disqualifying conditions. J Athl Train. 2014;49(1):102-20. doi: 10.4085/1062-6050-48.6.05. PubMed PMID: rayyan-344410877.

2. American Academy of Pediatrics, American Academy of Family Physicians, American College of Sports Medicine, American Medical Society for Sports Medicine, American Orthopaedic Society for Sports Medicine, American Osteopathic Academy of Sports Medicine. Preparticipation Physical Evaluation, 5th Edition: American Academy of Pediatrics; 2019. 240 p.

3. Black HR, Sica D, Ferdin, K, White WB. Eligibility and Disqualification Recommendations for Competitive Athletes With Cardiovascular Abnormalities: Task Force 6: Hypertension: A Scientific Statement from the American Heart Association and the American College of Cardiology. Circulation. 2015;132(22):e298-302. doi: 10.1161/cir.0000000000000242. PubMed PMID: rayyan-344410682.

4. Maron BJ, Levine BD, Washington RL, Baggish AL, Kovacs RJ, Maron MS. Eligibility and Disqualification Recommendations for Competitive Athletes With Cardiovascular Abnormalities: Task Force 2: Preparticipation Screening for Cardiovascular Disease in Competitive Athletes: A Scientific Statement From the American Heart Association and American College of Cardiology. Circulation. 2015;132(22):e267-72. doi: 10.1161/cir.0000000000000238. PubMed PMID: rayyan-344411718.

5. Maron BJ, Zipes DP, Kovacs RJ. Eligibility and Disqualification Recommendations for Competitive Athletes With Cardiovascular Abnormalities: Preamble, Principles, and General Considerations: A Scientific Statement From the American Heart Association and American College of Cardiology. Circulation. 2015;132(22):e256-61. doi: 10.1161/cir.0000000000000236. PubMed PMID: rayyan-344411721.

6. Löllgen H, Börjesson M, Cummiskey J, Bachl N, Debruyne A. The Pre-Participation Examination in Sports: EFSMA Statement on ECG for Pre-Participation Examination. Deutsche Zeitschrift für Sportmedizin. 2015;66(6):151-5 doi.

7. Ionescu AM, Pitsiladis YP, Rozenstoka S, Bigard X, Löllgen H, Bachl N, et al. Preparticipation medical evaluation for elite athletes: EFSMA recommendations on standardised preparticipation evaluation form in European countries. BMJ Open Sport Exerc Med. 2021;7(4):e001178. doi: 10.1136/bmjsem-2021-001178. PubMed PMID: rayyan-344411374.

8. Drezner JA, O'Connor FG, Harmon KG, Fields KB, Asplund CA, Asif IM, et al. AMSSM Position Statement on Cardiovascular Preparticipation Screening in Athletes: Current evidence, knowledge gaps, recommendations and future directions. Br J Sports Med. 2017;51(3):153-67. doi: 10.1136/bjsports-2016-096781. PubMed PMID: rayyan-344411013.

9. Fritsch P, Ehringer-Schetitska D, Dalla Pozza R, Jokinen E, Herceg-Cavrak V, Hidvegi E, et al. Cardiovascular pre-participation screening in young athletes: Recommendations of the Association of European Paediatric Cardiology. Cardiol Young. 2017;27(9):1655-60. doi: 10.1017/s1047951117001305. PubMed PMID: rayyan-344411124.

10. Baggish AL, Battle RW, Beaver TA, Border WL, Douglas PS, Kramer CM, et al. Recommendations on the Use of Multimodality Cardiovascular Imaging in Young Adult Competitive Athletes: A Report from the American Society of Echocardiography in Collaboration with the Society of Cardiovascular Computed Tomography and the Society for Cardiovascular Magnetic Resonance. J Am Soc Echocardiogr. 2020;33(5):523-49. doi: 10.1016/j.echo.2020.02.009. PubMed PMID: rayyan-344410584.

11. American College of Sports Medicine. ACSM's Guidelines for Exercise Testing and Prescription, 11th Edition. 11th ed2021.

12. Thornton JS, Frémont P, Khan K, Poirier P, Fowles J, Wells GD, et al. Physical Activity Prescription: A Critical Opportunity to Address a Modifiable Risk Factor for the Prevention and Management of Chronic Disease: A Position Statement by the Canadian Academy of Sport and Exercise Medicine: Erratum. Clin J Sport Med. 2020;30(6):616. doi: 10.1097/jsm.0000000000000664. PubMed PMID: rayyan-344410479.

13. Physical Activity and Exercise During Pregnancy and the Postpartum Period: ACOG Committee Opinion, Number 804. Obstet Gynecol. 2020;135(4):e178-e88. doi: 10.1097/aog.0000000000003772. PubMed PMID: 32217980.

14. Bø K, Artal R, Barakat R, Brown WJ, Davies GAL, Dooley M, et al. Exercise and pregnancy in recreational and elite athletes: 2016/2017 evidence summary from the IOC expert group meeting, Lausanne. Part 5. Recommendations for health professionals and active women. Br J Sports Med. 2018;52(17):1080-5. doi: 10.1136/bjsports-2018-099351. PubMed PMID: rayyan-344410690.

15. Kenjale AA, Hornsby WE, Crowgey T, Thomas S, Herndon JE, Khouri MG, et al. Pre-exercise participation cardiovascular screening in a heterogeneous cohort of adult cancer patients. The oncologist. 2014;19(9):999-1005.

16. Campbell KL, Winters-Stone KM, Wiskemann J, May AM, Schwartz AL, Courneya KS, et al. Exercise Guidelines for Cancer Survivors: Consensus Statement from International Multidisciplinary Roundtable. Med Sci Sports Exerc. 2019;51(11):2375-90. doi: 10.1249/mss.0000000000002116. PubMed PMID: 31626055; PubMed Central PMCID: PMCPMC8576825.

17. Wingfield K, Matheson GO, Meeuwisse WH. Preparticipation evaluation: an evidence-based review. Clinical Journal of Sport Medicine. 2004;14(3):109-22.

18. Carek PJ, Futrell M, Hueston WJ. The preparticipation physical examination history: who has the correct answers? Clin J Sport Med. 1999;9(3):124-8. Epub 1999/10/08. doi: 10.1097/00042752-199907000-00002. PubMed PMID: 10512339.

19. Narducci DM, Diamond AB, Bernhardt DT, Roberts WO. COVID Vaccination in Athletes and Updated Interim Guidance on the Preparticipation Physical Examination During the SARS-Cov-2 Pandemic. Clin J Sport Med. 2022;32(1):e1-e6. doi: 10.1097/jsm.0000000000000981. PubMed PMID: rayyan-344411896.

20. Diamond AB, Narducci DM, Roberts WO, Bernhardt DT, LaBella CR, Moffatt KA, et al. Interim Guidance on the Preparticipation Physical Examination for Athletes During the SARS-CoV-2 Pandemic. Clin J Sport Med. 2021;31(1):1-6. doi: 10.1097/jsm.0000000000000892. PubMed PMID: rayyan-344410973.

21. Carek PJ, Mainous A. The preparticipation physical examination for athletics: a systematic review of current recommendations. BMJ. 2003;327(7418):E170-E3.

22. Dixit S, DiFiori J. Prevalence of hypertension and prehypertension in collegiate student athletes. Clinical Journal of Sport Medicine. 2006;16(5):440.

23. Selected Issues in Injury and Illness Prevention and the Team Physician: A Consensus Statement. Curr Sports Med Rep. 2016;15(1):48-59. doi: 10.1249/jsr.0000000000000231. PubMed PMID: rayyan-344410454.

24. Brooks CD, Kujawska A, Patel D. Cutaneous allergic reactions induced by sporting activities. Sports Medicine. 2003;33:699-708.

25. Metelitsa A, Barankin B, Lin AN. Diagnosis of sports-related dermatoses. International journal of dermatology. 2004;43(2):113-9.

26. Stamos A, Mills S, Malliaropoulos N, Cantamessa S, Dartevelle JL, Gündüz E, et al. The European Association for Sports Dentistry, Academy for Sports Dentistry, European College of Sports and Exercise Physicians consensus statement on sports dentistry integration in sports medicine. Dent Traumatol. 2020;36(6):680-4. doi: 10.1111/edt.12593. PubMed PMID: rayyan-344412383.

27. Female Athlete Issues for the Team Physician: A Consensus Statement - 2017 Update. Medicine and Science in Sports and Exercise. 2018;50(5):1113-22. doi: doi:10.1249/MSS.0000000000001603. PubMed PMID: rayyan-399200722; PubMed Central PMCID: PMC 29652732.

28. Chang C, Putukian M, Aerni G, Diamond A, Hong G, Ingram Y, et al. Mental health issues and psychological factors in athletes: detection, management, effect on performance and prevention: American Medical Society for Sports Medicine Position Statement-Executive Summary. Br J Sports Med. 2020;54(4):216-20. doi: 10.1136/bjsports-2019-101583. PubMed PMID: rayyan-344410807.

29. Mountjoy M, Sundgot-Borgen J, Burke L, Carter S, Constantini N, Lebrun C, et al. The IOC consensus statement: beyond the Female Athlete Triad--Relative Energy Deficiency in Sport (RED-S). Br J Sports Med. 2014;48(7):491-7. doi: 10.1136/bjsports-2014-093502. PubMed PMID: rayyan-344411868.

30. Mountjoy M, Sundgot-Borgen J, Burke L, Ackerman KE, Blauwet C, Constantini N, et al. International Olympic Committee (IOC) Consensus Statement on Relative Energy Deficiency in Sport (RED-S): 2018 Update. Int J Sport Nutr Exerc Metab. 2018;28(4):316-31. Epub 2018/05/18. doi: 10.1123/ijsnem.2018-0136. PubMed PMID: 29771168.

31. Sundgot-Borgen J, Meyer NL, Lohman TG, Ackl, TR, Maughan RJ, et al. How to minimise the health risks to athletes who compete in weight-sensitive sports review and position statement on behalf of the Ad Hoc Research Working Group on Body Composition, Health and Performance, under the auspices of the IOC Medical Commission. Br J Sports Med. 2013;47(16):1012-22. doi: 10.1136/bjsports-2013-092966. PubMed PMID: rayyan-344412404.

32. Fredericson M, Kussman A, Misra M, Barrack MT, De Souza MJ, Kraus E, et al. The Male Athlete Triad-A Consensus Statement From the Female and Male Athlete Triad Coalition Part II: Diagnosis, Treatment, and Return-To-Play. Clin J Sport Med. 2021;31(4):349-66. doi: 10.1097/jsm.0000000000000948. PubMed PMID: rayyan-344411119.

33. Nattiv A, De Souza MJ, Koltun KJ, Misra M, Kussman A, Williams NI, et al. The Male Athlete Triad-A Consensus Statement From the Female and Male Athlete Triad Coalition Part 1: Definition and Scientific Basis. Clin J Sport Med. 2021;31(4):335–48. doi: 10.1097/JSM.0000000000000946. PubMed PMID: 34091537.

34. De Souza MJ, Nattiv A, Joy E, Misra M, Williams NI, Mallinson RJ, et al. 2014 Female Athlete Triad Coalition Consensus Statement on Treatment and Return to Play of the Female Athlete Triad: 1st International Conference held in San Francisco, California, May 2012 and 2nd International Conference held in Indianapolis, Indiana, May 2013. Br J Sports Med. 2014;48(4):289. doi: 10.1136/bjsports-2013-093218. PubMed PMID: rayyan-344410943.

35. Rauh MJ, Nichols JF, Barrack MT. Relationships among injury and disordered eating, menstrual dysfunction, and low bone mineral density in high school athletes: a prospective study. Journal of athletic training. 2010;45(3):243-52.

36. Thein-Nissenbaum JM, Rauh MJ, Carr KE, Loud KJ, McGuine TA. Menstrual irregularity and musculoskeletal injury in female high school athletes. Journal of athletic training. 2012;47(1):74-82.

37. Scholes D, LaCroix AZ, Ichikawa LE, Barlow WE, Ott SM. Change in bone mineral density among adolescent women using and discontinuing depot medroxyprogesterone acetate contraception. Archives of pediatrics & adolescent medicine. 2005;159(2):139-44.

38. Jacobi C, Fittig E, Bryson S, Wilfley D, Kraemer H, Taylor CB. Who is really at risk? Identifying risk factors for subthreshold and full syndrome eating disorders in a high-risk sample. Psychological medicine. 2011;41(9):1939-49.

39. Liechty JM, Lee MJ. Longitudinal predictors of dieting and disordered eating among young adults in the US. International Journal of Eating Disorders. 2013;46(8):790-800.

40. Francisco R, Narciso I, Alarcao M. Individual and relational risk factors for the development of eating disorders in adolescent aesthetic athletes and general adolescents. Eating and Weight Disorders-Studies on Anorexia, Bulimia and Obesity. 2013;18:403-11.

41. Gibbs JC, Nattiv A, Barrack MT, Williams NI, Rauh MJ, Nichols JF, et al. Low bone density risk is higher in exercising women with multiple triad risk factors. Med Sci Sports Exerc. 2014;46(1):167-76.

42. Gomes AR, Martins C, Silva L. Eating disordered behaviours in Portuguese athletes: The influence of personal, sport, and psychological variables. European Eating Disorders Review. 2011;19(3):190-200.

43. Thein-Nissenbaum JM, Rauh MJ, Carr KE, Loud KJ, McGuine TA. Associations between disordered eating, menstrual dysfunction, and musculoskeletal injury among high school athletes. journal of orthopaedic & sports physical therapy. 2011;41(2):60-9.

44. Rosen LW, Hough DO. Pathogenic weight-control behaviors of female college gymnasts. The Physician and Sportsmedicine. 1988;16(9):140-4.

45. Casa DJ, Guskiewicz KM, Anderson SA, Courson RW, Heck JF, Jimenez CC, et al. National athletic trainers' association position statement: preventing sudden death in sports. J Athl Train. 2012;47(1):96-118. doi: 10.4085/1062-6050-47.1.96. PubMed PMID: rayyan-344410781.

46. Mont L, Pelliccia A, Sharma S, Biffi A, Borjesson M, Brugada Terradellas J, et al. Pre-participation cardiovascular evaluation for athletic participants to prevent sudden death: Position paper from the EHRA and the EACPR, branches of the ESC. Endorsed by APHRS, HRS, and SOLAECE. Eur J Prev Cardiol. 2017;24(1):41-69. doi: 10.1177/2047487316676042. PubMed PMID: rayyan-344411831.

47. Pelliccia A, Sharma S, Gati S, Bäck M, Börjesson M, Caselli S, et al. 2020 ESC Guidelines on sports cardiology and exercise in patients with cardiovascular disease. European Heart Journal. 2021;42(1):17-96. doi: doi:10.1093/eurheartj/ehaa605. PubMed PMID: rayyan-399201647.

48. Zeppenfeld K, Tfelt-Hansen J, de Riva M, Winkel BG, Behr ER, Blom NA, et al. 2022 ESC Guidelines for the management of patients with ventricular arrhythmias and the prevention of sudden cardiac death. Eur Heart J. 2022;43(40):3997-4126. doi: 10.1093/eurheartj/ehac262. PubMed PMID: 36017572.

49. Risgaard B, Winkel BG, Jabbari R, Glinge C, Ingemann-Hansen O, Thomsen JL, et al. Sports-related sudden cardiac death in a competitive and a noncompetitive athlete population aged 12 to 49 years: data from an unselected nationwide study in Denmark. Heart rhythm. 2014;11(10):1673-81.

50. Erbel R, Möhlenkamp S, Moebus S, Schmermund A, Lehmann N, Stang A, et al. Coronary risk stratification, discrimination, and reclassification improvement based on quantification of subclinical coronary atherosclerosis: the Heinz Nixdorf Recall study. Journal of the American College of Cardiology. 2010;56(17):1397-406.

51. Yeboah J, McClelland RL, Polonsky TS, Burke GL, Sibley CT, O'Leary D, et al. Comparison of novel risk markers for improvement in cardiovascular risk assessment in intermediate-risk individuals. Jama. 2012;308(8):788-95.

52. Gellish RL, Goslin BR, Olson RE, McDONALD A, Russi GD, Moudgil VK. Longitudinal modeling of the relationship between age and maximal heart rate. Medicine and science in sports and exercise. 2007;39(5):822-9.

53. Orton S-M, Herrera BM, Yee IM, Valdar W, Ramagopalan SV, Sadovnick AD, et al. Sex ratio of multiple sclerosis in Canada: a longitudinal study. The Lancet Neurology. 2006;5(11):932-6.

54. Hesse CM, Tinius RA, Pitts BC, Olenick AA, Blankenship MM, Hoover DL, et al. Assessment of endpoint criteria and perceived barriers during maximal cardiorespiratory fitness testing among pregnant women. The Journal of Sports Medicine and Physical Fitness. 2017;58(12):1844-51.

55. Patnaik JL, Byers T, DiGuiseppi C, Dabelea D, Denberg TD. Cardiovascular disease competes with breast cancer as the leading cause of death for older females diagnosed with breast cancer: a retrospective cohort study. Breast Cancer Res. 2011;13(3):R64. Epub 2011/06/22. doi: 10.1186/bcr2901. PubMed PMID: 21689398; PubMed Central PMCID: PMCPMC3218953.

56. Corrado D, Basso C, Pavei A, Michieli P, Schiavon M, Thiene G. Trends in sudden cardiovascular death in young competitive athletes after implementation of a preparticipation screening program. Jama. 2006;296(13):1593-601.

57. Casa DJ, Almquist J, Anderson SA, Baker L, Bergeron MF, Biagioli B, et al. The inter-association task force for preventing sudden death in secondary school athletics programs: best-practices recommendations. J Athl Train. 2013;48(4):546-53. doi: 10.4085/1062-6050-48.4.12. PubMed PMID: rayyan-344410778.

58. Corrado D, Basso C, Schiavon M, Thiene G. Screening for hypertrophic cardiomyopathy in young athletes. New England Journal of Medicine. 1998;339(6):364-9.

59. Baggish AL, Hutter Jr AM, Wang F, Yared K, Weiner RB, Kupperman E, et al. Cardiovascular screening in college athletes with and without electrocardiography: a cross-sectional study. Annals of internal medicine. 2010;152(5):269-75.

60. Drezner JA, Owens DS, Prutkin JM, Salerno JC, Harmon KG, Prosise S, et al. Electrocardiographic screening in national collegiate athletic association athletes. The American Journal of Cardiology. 2016;118(5):754-9.

61. Steinvil A, Chundadze T, Zeltser D, Rogowski O, Halkin A, Galily Y, et al. Mandatory electrocardiographic screening of athletes to reduce their risk for sudden death: proven fact or wishful thinking? Journal of the American College of Cardiology. 2011;57(11):1291-6.

62. Johri AM, Poirier P, Dorian P, Fournier A, Goodman JM, McKinney J, et al. Canadian Cardiovascular Society/Canadian Heart Rhythm Society Joint Position Statement on the Cardiovascular Screening of Competitive Athletes. Can J Cardiol. 2019;35(1):1-11. doi: 10.1016/j.cjca.2018.10.016. PubMed PMID: rayyan-344411415.

63. Oxborough D, Augustine D, Gati S, George K, Harkness A, Mathew T, et al. A guideline update for the practice of echocardiography in the cardiac screening of sports participants: a joint policy statement from the British Society of Echocardiography and Cardiac Risk in the Young. Echo Res Pract. 2018;5(1):G1-g10. doi: 10.1530/erp-17-0075. PubMed PMID: rayyan-344411976.

64. Hainline B, Drezner J, Baggish A, Harmon KG, Emery MS, Myerburg RJ, et al. Interassociation Consensus Statement on Cardiovascular Care of College Student-Athletes. J Athl Train. 2016;51(4):344-57. doi: 10.4085/j.jacc.2016.03.527. PubMed PMID: rayyan-344411243.

65. Mahmood S, Lim L, Akram Y, Alford-Morales S, Sherin K. Screening for sudden cardiac death before participation in high school and collegiate sports: American College of Preventive Medicine position statement on preventive practice. Am J Prev Med. 2013;45(1):130-3. doi: doi:10.1016/j.amepre.2013.04.002. PubMed PMID: rayyan-344411692.

66. Biffi A, Delise P, Zeppilli P, Giada F, Pelliccia A, Penco M, et al. Italian cardiological guidelines for sports eligibility in athletes with heart disease: part 1. J Cardiovasc Med (Hagerstown). 2013;14(7):477-99. doi: 10.2459/JCM.0b013e32835f6a21. PubMed PMID: rayyan-344410667.

67. Biffi A, Delise P, Zeppilli P, Giada F, Pelliccia A, Penco M, et al. Italian cardiological guidelines for sports eligibility in athletes with heart disease: part 2. J Cardiovasc Med (Hagerstown). 2013;14(7):500-15. doi: 10.2459/JCM.0b013e32835fcb8a. PubMed PMID: rayyan-344410668.

68. Delise P, Mos L, Sciarra L, Basso C, Biffi A, Cecchi F, et al. Italian Cardiological Guidelines (COCIS) for Competitive Sport Eligibility in athletes with heart disease: update 2020. J Cardiovasc Med (Hagerstown). 2021;22(11):874-91. doi: 10.2459/jcm.0000000000001186. PubMed PMID: 33882535.

69. Maron BJ, Doerer JJ, Haas TS, Tierney DM, Mueller FO. Sudden deaths in young competitive athletes: analysis of 1866 deaths in the United States, 1980–2006. Circulation. 2009;119(8):1085-92.

70. Marcadet DM, Pavy B, Bosser G, Claudot F, Corone S, Douard H, et al. French Society of Cardiology guidelines on exercise tests (part 1): Methods and interpretation. Arch Cardiovasc Dis. 2018;111(12):782-90. Epub 20180806. doi: 10.1016/j.acvd.2018.05.005. PubMed PMID: 30093254.

71. Marcadet DM, Pavy B, Bosser G, Claudot F, Corone S, Douard H, et al. French Society of Cardiology guidelines on exercise tests (part 2): Indications for exercise tests in cardiac diseases. Arch Cardiovasc Dis. 2019;112(1):56-66. Epub 20180806. doi: 10.1016/j.acvd.2018.07.001. PubMed PMID: 30093255.

72. Crescenzi C, Zorzi A, Vessella T, Martino A, Panattoni G, Cipriani A, et al. Predictors of left ventricular scar using cardiac magnetic resonance in athletes with apparently idiopathic ventricular arrhythmias. Journal of the American Heart Association. 2021;10(1):e018206.

73. Rizzo M, Spataro A, Cecchetelli C, Quaranta F, Livrieri S, Sperandii F, et al. Structural cardiac disease diagnosed by echocardiography in asymptomatic young male soccer players: implications for pre-participation screening. British journal of sports medicine. 2012;46(5):371-3.

74. Galderisi M, Cardim N, D'Andrea A, Bruder O, Cosyns B, Davin L, et al. The multi-modality cardiac imaging approach to the Athlete's heart: an expert consensus of the European Association of Cardiovascular Imaging. Eur Heart J Cardiovasc Imaging. 2015;16(4):353. doi: 10.1093/ehjci/jeu323. PubMed PMID: rayyan-344411141.

75. Pelliccia A, Caselli S, Sharma S, Basso C, Bax JJ, Corrado D, et al. European Association of Preventive Cardiology (EAPC) and European Association of Cardiovascular Imaging (EACVI) joint position statement: recommendations for the indication and interpretation of cardiovascular imaging in the evaluation of the athlete's heart. Eur Heart J. 2018;39(21):1949-69. doi: 10.1093/eurheartj/ehx532. PubMed PMID: rayyan-344412022.

76. Drezner JA, Sharma S, Baggish A, Papadakis M, Wilson MG, Prutkin JM, et al. International criteria for electrocardiographic interpretation in athletes: Consensus statement. Br J Sports Med. 2017;51(9):704-31. doi: 10.1136/bjsports-2016-097331. PubMed PMID: rayyan-344411016.

77. Quattrini FM, Pelliccia A, Assorgi R, DiPaolo FM, Squeo MR, Culasso F, et al. Benign clinical significance of J-wave pattern (early repolarization) in highly trained athletes. Heart Rhythm. 2014;11(11):1974-82.

78. Calore C, Zorzi A, Sheikh N, Nese A, Facci M, Malhotra A, et al. Electrocardiographic anterior T-wave inversion in athletes of different ethnicities: differential diagnosis between athlete's heart and cardiomyopathy. European heart journal. 2016;37(32):2515-27.

79. Marcus FI, McKenna WJ, Sherrill D, Basso C, Bauce B, Bluemke DA, et al. Diagnosis of arrhythmogenic right ventricular cardiomyopathy/dysplasia: proposed modification of the Task Force Criteria. Eur Heart J. 2010;31(7):806-14. Epub 2010/02/23. doi: 10.1093/eurheartj/ehq025. PubMed PMID: 20172912; PubMed Central PMCID: PMCPMC2848326.

80. Cohen M, Triedman J, Cannon B, Davis A, Drago F, Janousek J, et al. Pediatric and Congenital Electrophysiology Society (PACES). Heart Rhythm Society (HRS). 2012;9(6):1006-24.

81. DAUBERT C, OLLITRAULT J, DESCAVES C, MABO P, RITTER P, GOUFFAULT J. Failure of the exercise test to predict the anterograde refractory period of the accessory pathway in Wolff Parkinson White syndrome. Pacing and Clinical Electrophysiology. 1988;11(8):1130-8.

82. Corrado D, Basso C, Leoni L, Tokajuk B, Turrini P, Bauce B, et al. Three-dimensional electroanatomical voltage mapping and histologic evaluation of myocardial substrate in right ventricular outflow tract tachycardia. Journal of the American College of Cardiology. 2008;51(7):731-9.

83. Sheikh N, Papadakis M, Ghani S, Zaidi A, Gati S, Adami PE, et al. Comparison of electrocardiographic criteria for the detection of cardiac abnormalities in elite black and white athletes. Circulation. 2014;129(16):1637-49.

84. Harmon KG, Drezner JA, Maleszewski JJ, Lopez-Anderson M, Owens D, Prutkin JM, et al. Pathogeneses of sudden cardiac death in national collegiate athletic association athletes. Circulation: Arrhythmia and Electrophysiology. 2014;7(2):198-204.

85. Weiler JM, Brannan JD, Randolph CC, Hallstrand TS, Parsons J, Silvers W, et al. Exercise-induced bronchoconstriction update—2016. Journal of Allergy and Clinical Immunology. 2016;138(5):1292-5. e36.

86. Anderson SD, Pearlman DS, Rundell KW, Perry CP, Boushey H, Sorkness CA, et al. Reproducibility of the airway response to an exercise protocol standardized for intensity, duration, and inspired air conditions, in subjects with symptoms suggestive of asthma. Respiratory research. 2010;11:1-12.

87. Dodge WF, West EF, Smith EH, Bunce III H. Proteinuria and hematuria in schoolchildren: epidemiology and early natural history. The Journal of pediatrics. 1976;88(2):327-47.

88. Feinstein R, LaRussa J, Wang-Dohlman A, Bartolucci A. Screening adolescent athletes for exercise-induced asthma. Clinical journal of sport medicine: official journal of the Canadian Academy of Sport Medicine. 1996;6(2):119-23.

89. Eliakim A, Nemet D, Constantini N. Screening blood tests in members of the Israeli National Olympic team. Journal of sports medicine and physical fitness. 2002;42(2):250.

90. Fallon KE. Screening for haematological and iron-related abnormalities in elite athletes—analysis of 576 cases. Journal of Science and Medicine in Sport. 2008;11(3):329-36.

91. Eisenmann JC. Blood lipids and lipoproteins in child and adolescent athletes. Sports medicine. 2002;32:297-307.

92. Gomez JE, Landry GL, Bernhardt DT. Critical evaluation of the 2-minute orthopedic screening examination. American Journal of Diseases of Children. 1993;147(10):1109-13.

93. Smith J, Laskowski ER, editors. The preparticipation physical examination: Mayo Clinic experience with 2,739 examinations. Mayo Clinic Proceedings; 1998: Elsevier.

94. Newman P, Adams R, Waddington G. Two simple clinical tests for predicting onset of medial tibial stress syndrome: shin palpation test and shin oedema test. British Journal of Sports Medicine. 2012;46(12):861-4.

95. Ugalde V, Brockman C, Bailowitz Z, Pollard CD. Single leg squat test and its relationship to dynamic knee valgus and injury risk screening. Pm&r. 2015;7(3):229-35.

96. Hewett TE, Myer GD, Ford KR, Heidt Jr RS, Colosimo AJ, McLean SG, et al. Biomechanical measures of neuromuscular control and valgus loading of the knee predict anterior cruciate ligament injury risk in female athletes: a prospective study. The American journal of sports medicine. 2005;33(4):492-501.

97. Hewett TE, Myer GD, Ford KR, Slauterbeck JR. Preparticipation physical examination using a box drop vertical jump test in young athletes: the effects of puberty and sex. Clinical Journal of Sport Medicine. 2006;16(4):298-304.

98. Panel on Prevention of Falls in Older Persons AGSaBGS. Summary of the Updated American Geriatrics Society/British Geriatrics Society clinical practice guideline for prevention of falls in older persons. J Am Geriatr Soc. 2011;59(1):148-57. Epub 2011/01/14. doi: 10.1111/j.1532-5415.2010.03234.x. PubMed PMID: 21226685.

99. Neal TL, Diamond AB, Goldman S, Liedtka KD, Mathis K, Morse ED, et al. Interassociation recommendations for developing a plan to recognize and refer student-athletes with psychological concerns at the secondary school level: a consensus statement. J Athl Train. 2015;50(3):231-49. doi: 10.4085/1062-6050-50.3.03. PubMed PMID: rayyan-344411903.

100. Carroll JF, McGinley JJ. A screening form for identifying mental health problems in alcohol/other drug dependent persons. Alcoholism Treatment Quarterly. 2001;19(4):33-47.
